# Metabolomics biomarkers and related pathways in endometrial cancer: A systematic review and meta‐analysis

**DOI:** 10.1111/jcmm.18401

**Published:** 2024-05-28

**Authors:** Xingxu Yan, Shan Zhao, Junjie He, Guijiang Sun, Fang Wang, Haoran Ding, Wenqing Zhang, Wenxiu Qian, Xiaomeng Li, Yuyan Wang, Yubo Li

**Affiliations:** ^1^ State Key Laboratory of Component‐based Chinese Medicine Tianjin University of Traditional Chinese Medicine Tianjin China; ^2^ Department of Kidney Disease and Blood Purification The Second Hospital of Tianjin Medical University Tianjin China; ^3^ The Key Laboratory of Carcinogenesis and Translational Research (Ministry of Education), Department of Thoracic Medical Oncology Beijing Institute of Cancer Research Beijing China

**Keywords:** endometrial cancer, meta‐analysis, metabolic pathways, metabolomics, systematic review

## Abstract

Endometrial cancer (EC) is the fourth most common malignant tumour afflicting postmenopausal women in the world. Although advances have been made in the metabolomics of EC, the controversial findings limited the clinical applicability of the research results. Therefore, we aimed to reveal metabolic characteristics disorders in EC. We included studies comparing metabolite levels between EC and controls by searching PubMed, EMBASE, Cochrane Library, Web of Science and other source databases. Biomarkers and metabolic pathways were subjected to meta‐analysis by calculating the standardized mean difference (SMD) with 95% confidence intervals (CI), and summarizing the effect size using the inverse variance random effect model. Thirty studies identified the differential metabolites of EC. A total of 8 studies involved 4,006 participants with good quality were included. In terms of biomarkers, estrone and proline increased significantly in EC, while glutamine and phosphatidylcholine diacyl C32:2 decreased significantly in EC, with low heterogeneity. In terms of metabolic pathways, the levels of branched‐chain amino acid metabolism and sphingolipid metabolism increased significantly in EC with low heterogeneity. This review highlights the significance of above four biomarkers and two metabolic pathways in EC, which will help to realize the accurate screening and diagnosis of EC.

## 1 INTRODUCTION

Endometrial cancer (EC) is the fourth common malignant tumour in the global female population, and its morbidity and mortality have continued to increase in the past decade.^1^, ^2^, ^3^ According to the latest estimates of the American Cancer Society (ACS), there will be 66,200 new cases and 13,030 deaths of EC in 2023. It is estimated that the incidence of EC worldwide will increase by more than 50% by 2040, seriously affecting the health of women.^4^, ^5^ EC primarily affects postmenopausal women, so the ACS recommends that women in menopause should be informed about the risks and symptoms of EC and that they seek screening and evaluation when symptoms develop.^1^

Clinically, due to the subjective heterogeneity of transvaginal ultrasound (which largely depends on the subjective judgement of clinicians) and the invasiveness of hysteroscopy‐guided biopsy (which may lead to discomfort, bleeding and infection), biomarkers can be used as molecular markers for EC early screening and diagnosis, given its advantages of non‐invasiveness, convenience, objectivity and accuracy.^6^, ^7^, ^8^ At present, there are potential biomarkers such as cancer antigen 125 (CA125) and human epididymis protein 4 (HE4) that can be used as indicators for the screening and evaluation of EC. However, their low sensitivity and/or low specificity limit their clinical diagnosis performance (CA125 has 35% detection sensitivity and 83% specificity; HE4 has 71% detection sensitivity and 87% specificity), resulting in the absence of robust and reliable biomarkers for EC early screening and diagnosis.^9^

Considering this situation, metabolomics is an advantageous technique for identifying potential biomarkers of diseases, which can provide a reference for finding potential biomarkers for EC screening and diagnosis.^10^, ^11^ In recent years, metabolomics technology has been widely used in the study of EC, revealing the metabolic level disorder in human EC. For example, in our previous study, we used two independent cohorts based on two centres to screen and verify potential biomarkers in the serum of EC, highlighting the importance of lipid metabolites as diagnostic biomarkers of EC.^12^ In addition, other studies also reported that metabolic pathways such as carbohydrate metabolism and amino acid metabolism are significantly disturbed.^13^, ^14^, ^15^ As can be seen that these results suggest that metabolomics can help to promote the early screening and diagnosis of EC.

Although the current EC metabolomics research has made progress in disease detection, the statistical power of individual biomarker and metabolic pathway results is greatly reduced due to the small sample size of most metabolomics studies (<1,000 participants). Furthermore, although studies have reported changes in many biomarkers and metabolic pathways in EC, the clinical applicability of the findings is limited due to inconsistent results in some studies (e.g., phosphatidylcholine diacyl [PC aa] C42:5 and estrone‐sulfate have been reported to have conflicting change trends or significance in different studies).^16^, ^17^, ^18^, ^19^

In view of this, meta‐analysis is a statistical technique that combines the results of different studies on the same topic.^20^ It selects the appropriate statistical model, weights the data according to the different amount of information in each study and finally merges the effect sizes of the included studies. By integrating the previously published EC metabolomics research results, meta‐analysis can increase the sample size and obtain the analysis results with the best statistical power, thus overcoming the problems of small sample size and inadequate statistical strength.^21^ Moreover, meta‐analysis can also obtain accurate estimates of biomarkers and metabolic pathways related to EC, thus explaining conflicting evidence or inconsistency in the research results.^22^ However, to date, no study has quantitatively evaluated the EC metabolomic characteristics changes, although study has systematically reviewed the diagnostic and prognostic biomarkers of endometrial diseases.^23^

Thus, the aim of the present study was to reveal unique metabolomics characteristics changes in human EC. We first systematically reviewed the literature to identify eligible EC metabolomics studies, and then performed quantitative meta‐analysis of 135 biomarkers and 16 metabolic pathways associated with EC. In addition, we also performed subgroup analysis, sensitivity analysis and meta‐regression analysis to explore sources of heterogeneity.

## 2 METHODS AND MATERIALS

### 2.1 Data source and search strategy

This review was performed based on the Preferred Reporting Items for Systematic Reviews and Meta‐analysis (PRISMA) guidelines (Appendix A). The protocol has been registered in the PROSPERO registry (CRD42022332697). With the aid and expertise of an information specialist, we searched the PubMed, EMBASE, Cochrane Library and Web of Science databases for studies published from the earliest available online to 28 November 2023. The search terms used included ‘metabolomics’, ‘metabolome’, ‘lipidomics’, ‘endometrial neoplasms’ and its variants. The detailed search strategy is presented in Table S1. We also searched clinical trial register (ClinicalTrials.gov), two prepublication server depositories (bioRxiv and medRxiv) and grey literature (OpenGrey). We manually screened the reference lists of the selected articles and contacted experts in the field to identify any additional references not found during the search of the electronic databases.

### 2.2 Study selection and eligibility criteria

We removed the duplicate records by using Endnote X9 software (Stanford, USA). The titles, abstracts and full texts of articles were screened independently by two researchers (X.Y. and F.W.). Any disagreements regarding the selection or inclusion of studies were discussed and resolved by a third researcher (J.H.).

We included studies that met the following inclusion criteria: (1) Human studies with EC (case–control, cross‐sectional or cohort studies); (2) studies using EC and non‐EC control samples for comparison; (3) studies using high‐throughput metabolomics techniques including nuclear magnetic resonance (NMR), gas chromatography, liquid chromatography, mass spectrometry (MS) or combinations of them, to identify metabolites in biological fluids or tissue samples; and (4) studies including human biological samples taken from patients before surgery.

We excluded studies that met the following exclusion criteria: (1) Studies involving animal models and pregnant women; (2) nonoriginal papers (reviews, commentaries, editorials or letters) and duplicate publications; (3) metabolomic methods were not used to measure metabolomic changes in samples; (4) studies involving metabolomic analysis of metabolites in cells; and (5) studies from other sources such as grey literature lacking sufficient information.

### 2.3 Data extraction and quality assessment

We extracted the information from each study in the data extraction form as follows: (1) general information: first author, year of publication and journal; (2) demographic information: location, age and sample size of participants, type of biological sample and type of control; (3) methodological information: detection techniques and methods; (4) outcome measurements: data on metabolites analysed, including mean and standard deviation (SD) [change trend] or odds ratio (OR) and 95% confidence intervals (CI) [association trend]; (5) study design. Data extraction was done independently by two researchers (X.Y. and W.Z.), and when inconsistent extraction results appeared, the third researcher (H.D.) discussed the decision with the two researchers.

To minimize risk of bias, this review used the 9‐point Newcastle–Ottawa Scale (NOS) to assess the methodological quality of observational studies in three categories, including selection of study populations, comparability between groups and measurement of exposure factors (for case–control studies) or outcomes (for cohort studies). A study with a score of not less than 6 was defined as high quality.^24^ Two researchers (X.Y. and S.Z.) independently participated in the quality assessment. In case of disagreement, a third researcher (W.Q.) confirmed the eligibility of the article.

### 2.4 Data synthesis and meta‐analysis

This review used STATA 12.0 software (StataCorp LP, College Station, TX) to perform meta‐analysis of biomarkers and metabolic pathways containing metabolite data points, respectively. Studies with insufficient data reported or low quality scores were not included in the meta‐analysis. For pathway analysis, we identified the primary metabolic pathways involved for each metabolite based on the Human Metabolome Database (HMDB) and Kyoto Encyclopedia of Genes and Genomes (KEGG) databases. Because of the differences in the methods and concentration units used to assess biomarker levels in the included studies, standardized mean difference (SMD) was used to estimate effect sizes. For studies that provided mean, SD and sample size for each metabolite, SMD was calculated using Cohen's D method. For studies that provided only OR and 95% CI for each metabolite, they were converted to SMD and their standard errors (SE) using the method of Hasselblad and Hedges before inclusion in meta‐analysis. In studies with multiple control groups, we included only the control group that was most age‐matched to the EC group. In studies that analysed biomarkers using multiple assays, we included only measurements using commercial assays (kit), not in‐house assays.

We pooled effect sizes using an inverse variance random effects model with statistical significance set at *p*‐value <0.05. Heterogeneity of included studies was assessed using the *p*‐value and *I* ^2^ statistic of Cochran's Q test. Significant heterogeneity was considered if *I* ^2^ > 50% and *p*‐value ≤0.10. Publication bias was assessed using Egger's test. A *p*‐value <0.10 indicated publication bias, and the trim and fill method was used to reduce the bias among the pooled estimates. To avoid low power of the tests, we assessed publication bias only for metabolic pathways with at least 10 data points.

To explore potential sources of heterogeneity for each metabolic pathway, subgroup analyses were performed by type of biological sample (cervicovaginal fluid/serum/plasma) and type of control group (healthy control/non‐cancer control). Sensitivity analysis was performed on studies without data conversion to determine the effect of this factor on the pooled effect size by excluding studies that used OR values converted to SMD values. In addition, a meta‐regression analysis was performed to investigate the effect of sample size on SMD. To avoid the risk of false analysis results, subgroup analysis, sensitivity analysis and meta‐regression analysis were performed only for metabolic pathways that contained at least 10 data points.

### 2.5 Assessment of evidence certainty

Grading of Recommendations Assessment, Development and Evaluation (GRADE) framework was used to assess the strength and quality of evidence for each outcome. According to GRADE, each outcome was categorized into four levels of evidence quality: very low, low, moderate and high. Evidence from observational studies started as low certainty. Defined criteria were applied to either decrease or increase quality of evidence rating (details are provided in Supplementary Methods [Data S1]).

## 3 RESULTS

### 3.1 Literature search results

**FIGURE 1:**
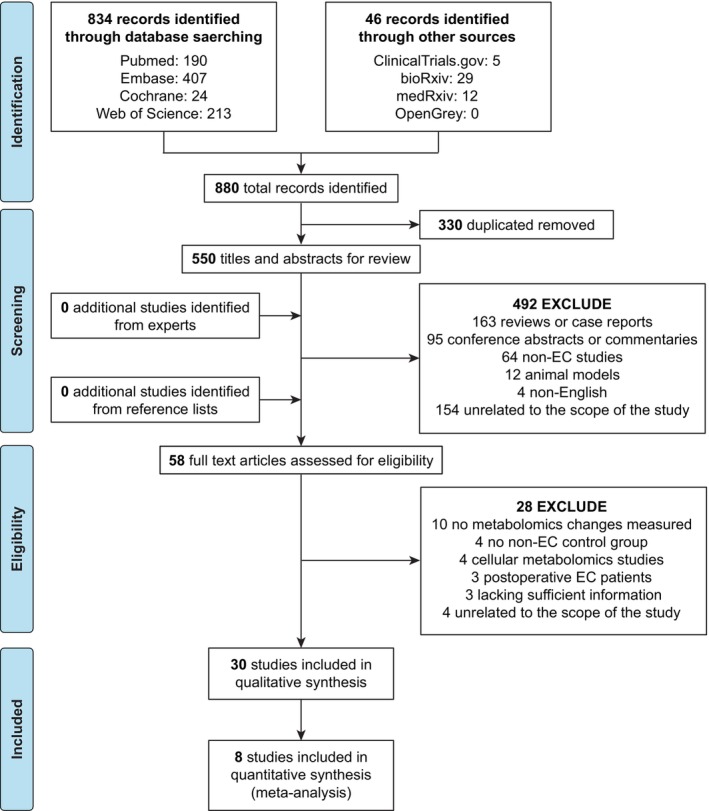
Flow diagram of literature search and study selection for biomarkers of endometrial cancer.

The flow of literature search and study selection is shown in Figure 1. A total of 880 articles were obtained after the initial search of the database. After removing duplicates, the titles and abstracts of the remaining 550 articles were independently screened by two researchers, and 492 articles were excluded. No additional relevant studies were found through systematic manual screening of the reference lists in the selected studies or by contacting experts in the field. We then reviewed the full text of the remaining 58 articles, and 28 articles were excluded. For example, the study of Strand E et al. was excluded because there was no non‐EC control group.^25^ A total of 30 studies met the inclusion and exclusion criteria and were included in the systematic review.

### 3.2 Characteristics of included studies

The characteristics of the 30 studies we included are summarized in Table 1. The included studies were published from 2010 to 2023. The sample size of each study ranged from 12 to 1,706 participants (the median study sample size was 80.5). Of these 30 studies, 28 studies were case–control studies and 2 studies were cohort studies; 26 studies used the MS platform, 3 studies used the NMR platform, and 1 study used both MS and NMR platforms; 17 studies used targeted metabolomics and 13 studies used non‐targeted metabolomics; 14 studies recruited from European, 11 studies recruited from Asian, and 5 studies recruited from North American; 13 studies measured serum samples, 7 studies measured endometrial tissue samples, 6 studies measured plasma samples, 2 studies measured urine samples, 1 study measured cervicovaginal fluid, and 1 study measured tissue and urine samples; 13 studies recruited healthy women as controls, 10 studies recruited women with benign uterine lesions as controls, 4 studies recruited women with unknown disease but normal endometrial pathology as controls, 2 studies recruited women without cancer as controls, and 1 study recruited women with breast cancer, benign uterine lesions and healthy women as controls.

**TABLE 1:** Summary of study characteristics for the included studies.

Study	Study design	Location	Patients/Controls (*n*)	Age (EC [mean ± SD]/non‐EC [mean ± SD])	Biological sample	Type of Controls	Technique and metabolite targets	Significant findings of metabolites
Bufa A, 2010, *Gynecol Endocrinol* ^26^	Case–Control	Hungary	13/10	(67.9 ± 8.5)/(58.7 ± 6.2)	Urine	Healthy female	GC–MS Targeted (23 androgen, corticoid and progesterone metabolites)	**Fold change**: 15 biomarkers (↓)
Audet‐Delage Y, 2018, *J Steroid Biochem Mol Biol* ^17^	Case–Control	Canada	246 (Type I EC: 202; Type II EC: 44)/110	(65.1 ± 8.9)/(NA)	Serum	Healthy female	GC–MS and LC–MS/MS Targeted (4 Adrenal precursors, 6 androgens, 3 parent oestrogens and 14 catechol oestrogen metabolites)	**Fold change:** **Type I EC:** 11 biomarkers (↑); **Type II EC:** 5 biomarkers (↑) **OR values:** **All cases:** 10 biomarkers (↑) and one biomarker (↓); **Type I EC:** 11 biomarkers (↑); **Type II EC:** 8 biomarkers (↑) and one biomarker (↓)
Skorupa A, 2021, *Sci Rep* ^13^	Case–Control	Poland	64 (G1: 14; G2: 33; G3: 17)/10	(68.47 ± 8.21)/(62.00 ± 7.32)	Tissue	Benign disorders female with normal tissue	HR MAS NMR Targeted (27 metabolites)	**Fold change:** **G1:** 10 biomarkers (↑) and 8 biomarkers (↓); **G2:** 14 biomarkers (↑) and 8 biomarkers (↓); **G3:** 11 biomarkers (↑) and 8 biomarkers (↓);
Shi K, 2018, *Cancer Sci* ^27^	Case–Control	China	46/46	(54 ± 8)/(57 ± 10)	Serum	Healthy female	UPLC‐Q‐TOF/MS Untargeted (7,646 and 2,579 variables in ESI (+) and ESI (−) mode, respectively)	**Fold change:** 4 biomarkers (↑)
Audet‐Delage Y, 2018, *Front Endocrinol* ^28^	Case–Control	Canada	36/18	(66.9 ± 8.8)/(58.9 ± 10.4)	Serum	Healthy female	UPLC‐MS Untargeted (1,592 compounds of known identity)	**Fold change:** 5 biomarkers (↑) and 5 biomarkers (↓)
Kozar N, 2021, *Adv Med Sci* ^29^	Case–Control	Slovenia	15/21	(64 ± 14)/(54 ± 19)	Serum	Benign disorders female	HPLC‐TQ/MS Targeted (232 metabolite [lipids, fatty acids, amino acids, nucleotides, ACs and amides])	**Fold change:** 4 biomarkers (↑)
Cummings M, 2019, *J Pathol* ^30^	Case–Control	United Kingdom	108 (Type I EC: 55; Type II EC: 53)/53	(66.19 ± 11.55)/(59.25 ± 13.75)	Tissue	Benign disorders female with normal tissue	LC–MS/MS Targeted (20 metabolites [COX‐derived prostanoids and their metabolites, and LOX‐derived monohydroxy fatty acid derivatives of AA, DGLA and LA])	**Fold change:** 2 biomarkers (↓)
Shafiee MN, 2020, *Int J Mol Sci* ^31^	Case–Control	United Kingdom	34/34	(63.44 ± 10.07)/(43.68 ± 13.12)	Tissue	Benign disorders female with normal tissue	LC–MS Untargeted (>20,000 peaks of lipid compounds)	**Fold change:** 14 biomarkers (↑) and 15 biomarkers (↓)
Altadill T, 2017, *Sci Rep* ^32^	Case–Control	Spain	39/17	(NA)/(NA)	Tissue	Female with normal tissue	UPLC‐ESI‐TOF‐MS Untargeted (8,146 and 7,558 features in ESI (+) and ESI (−) mode, respectively)	**Fold change:** 29 biomarkers (↑) and 13 biomarkers (↓)
Njoku K, 2021, *Cancers* ^33^	Case–Control	United Kingdom	67/69	(62.00 ± 11.11)/(46.00 ± 10.37)	Plasma	Female with normal tissue	UPLC MS/MS Untargeted (733 metabolites [amino acids, fatty acids, biogenic amines, sphingolipids, phospholipids, nucleotides, steroids, hexoses, vitamins and xenobiotics])	**Fold change:** 6 biomarkers (↑) and 2 biomarkers (↓)
Cheng SC, 2019, *Metabolomics* ^14^	Case–Control	China	21/33	(50.25 ± 9.25)/(50.00 ± 10.50)	Cervicovaginal fluid	Female with benign gynaecological condition	NMR Targeted (29 metabolites)	**Fold change:** 5 biomarkers (↑) and 5 biomarkers (↓)
Bahado‐Singh RO, 2017, *Metabolomics* ^34^	Case–Control	USA	56/60	(59.1 ± 12.8)/(59.2 ± 12.7)	Serum	Healthy female	1H NMR and DI‐LC–MS/MSTargeted (181 metabolites, 149 based on DI‐LC–MS/MS and 32 based on NMR)	**Fold change:** **1H NMR**: 3 biomarkers (↑) and one biomarker (↓); **DI‐LC–MS/MS**: 15 biomarkers (↑) and 34 biomarkers (↓) **OR values:** 4 biomarkers (↑) and 2 biomarkers (↓)
Troisi J, 2018, *J Proteome Res* ^15^	Case–Control	Italy	88/80	(66.00 ± 4.44)/(60.00 ± 7.41)	Serum	Healthy female	GC–MS Targeted (259 endogenous metabolites)	**Fold change:** 9 biomarkers (↑)
Jové M, 2016, *Oncotarget* ^35^	Case–Control	Spain	27/15	(NA)/ (NA)	Tissue	Female with normal tissue	LC‐ESI‐QTOF‐MS/MS Untargeted (717 molecular features)	**Fold change:** 3 biomarkers (↑)
Knific T, 2018, *J Steroid Biochem Mol Biol* ^18^	Case–Control	Slovenia	61/65	(65.1 ± 8.7)/(63.2 ± 9.4)	Plasma	Benign disorders female	FIA‐ESI‐MS/MS Targeted (41 ACs, 14 amino acids, hexoses, 15 sphingolipids and 92 GPLs [15 LPCs, 77 PCs])	**Fold change:** 2 biomarkers (↑) and 4 biomarkers (↓) **OR values:** 3 biomarkers (↓)
Schuhn A, 2022, *Arch Gynecol Obstet* ^36^	Case–Control	Germany	20/311 (BC: 140; Benign: 14; Control: 157)	(62 ± 9)/(51.51 ± 14.33)	Serum	BC, Benign disorders and healthy female	ESI‐MS/MS Targeted (18 Amino acids and 30 ACs)	**Fold change:** **EC vs. BC:** 7 biomarkers (↑) and one biomarker (↓); **EC vs. Benign:** one biomarker (↑) and 3 biomarkers (↓); **EC vs. Control:** 4 biomarkers (↑)
Dossus L, 2021, *Gynecol Oncol* ^19^	Cohort, population‐based study	Europe	853/853	(54.7 ± 7.5)/(54.7 ± 7.5)	Plasma	Free of cancer female	LC–MS/MS Targeted (40 ACs, 21 Amino acids, 21 Biogenic amines, 15 Sphingolipids, 90 GPLs and Sugars)	**OR values:** one biomarker (↑) and 2 biomarkers (↓)
Shao X, 2016, *Clin Chim Acta* ^37^	Case–Control	China	25/25	(NA)/(NA)	Urine	Healthy female	UPLC‐Q‐TOF/MS Untargeted	**Fold change:** 3 biomarkers (↑) and 2 biomarkers (↓)
Yan X, 2022, *Int J Cancer* ^12^	Case–Control	China	23/30	(58.65 ± 8.64)/(51.93 ± 7.46)	Serum	Healthy female	UPLC‐Q‐TOF/MS Untargeted (2,927 metabolite features)	**Fold change:** 5 biomarkers (↑) and 18 biomarkers (↓)
Yi R, 2022, *Front Oncol* ^38^	Case–Control	China	44/43	**Tissue** (48.2 ± 13.0)/(39.8 ± 5.3); **Urine** (44.0 ± 11.6)/ (44.3 ± 9.1)	Tissue/urine	Benign disorders female	HPLC‐Q‐TOF‐MS/MS Untargeted (4,410 and 8,066 metabolite features in tissue and urine, respectively)	**Fold change:** **Tissue:** 47 biomarkers (↑) and 27 biomarkers (↓); **Urine:** 25 biomarkers (↓)
Lépine J, 2010, *J Clin Endocrinol Metab* ^16^	Case–Control	Canada	126/110	(64.8 ± 9.1)/(58.3 ± 5.6)	Serum	Healthy female	GC/MS and LC–MS Targeted (GC/MS for E1 and E2, and LC–MS for E1‐S)	**Fold change:** 3 biomarkers (↑)
Zhao SS, 2022, *Microbiol Spectr* ^39^	Case–Control	China	18/18	(54.94 ± 9.7)/(47.47 ± 6.88)	Plasma	Benign disorders female	HPLC‐MS/MS Targeted (20 amino acids and 18 fatty acids)	**Fold change:** 7 biomarkers (↑) and one biomarker (↓)
Hishinuma E, 2023, *Cancer Metab* ^40^	Case–Control	China	142/154	(59.29 ± 11.67)/(59.03 ± 12.31)	Plasma	Healthy female	UPLC‐MS/MS Targeted (628 metabolites)	**Fold change:** 111 biomarkers (↑) and 148 biomarkers (↓)
Boyd AE, 2023, *Cancers* ^41^	Case–Control	USA	22/22	(61.0 ± 12.0)/ (61.0 ± 12.0)	Tissue	Female with disease‐free tissue	LC‐ESI‐MS/MS Targeted (10 ceramides, 4 long‐chain bases, 10 monohexosylceramides, 10 sphingomyelins, 10 lactosylceramides)	**Fold Change:** 18 biomarkers (↑)
Arda Düz S, 2022, *Arch Gynecol Obstet* ^42^	Case–Control	Turkey	17/18	(53.5 ± 7.9)/ (49 ± 7.1)	Tissue	Benign disorders female	HR MAS NMR Untargeted	**Fold Change:** 17 biomarkers (↑)
Troisi J, 2022, *Biomolecules* ^43^	Case–Control	Italy	90 (EC and control)	(NA)/(NA)	Serum	Healthy female	GC–MS Untargeted	**Fold Change:** 3 biomarkers (↑) and 9 biomarkers (↓)
Gu M, 2021, *Neoplasma* ^44^	Case–Control	China	60 (Type I EC: 30; Type II EC: 30)/30	(NA)/ (NA)	Serum	Healthy female	GC–MS Untargeted (950 metabolite features)	**Fold Change:** **Type I EC:** 7 biomarkers (↑) and 20 biomarkers (↓); **Type II EC:** 6 biomarkers (↑) and 28 biomarkers (↓)
Breeur M, 2022, *BMC Med* ^45^	Cohort, population‐based study	Europe	689/689	(54.3 ± 7.84)/(54.3 ± 7.83)	Plasma	Cancer‐free female	LC–MS/MS and FIA‐MS/MS Targeted (117 metabolites [amino acids, biogenic amines and other metabolites])	**OR values:** 3 biomarkers (↑) and 6 biomarkers (↓)
Hao C, 2023, *J Obstet Gynaecol Res* ^46^	Case–Control	China	8 (Type I EC: 4; Type III EC: 4)/4	(Type I EC: [66.5 ± 11.9]; Type III EC: [50.8 ± 4.3])/(50.6 ± 4.8)^a^	Serum	Benign disorders female	LC–MS/MS Untargeted	**Fold change:** **Type I EC:** 214 biomarkers (↑) and 207 biomarkers (↓); **Type III EC:** 160 biomarkers (↑) and 215 biomarkers (↓)
Cheng F, 2023, *Anal Bioanal Chem* ^47^	Case–Control	China	18/20	(46.94 ± 7.48)/(44.30 ± 7.54)	Serum	Healthy female	UPLC‐MS/MS Targeted (439 and 177 lipids in ESI (+) and ESI (−) mode, respectively)	**Fold change:** 31 biomarkers (↑) and 8 biomarkers (↓)

*Note*: ↑, biomarker was up‐regulated in EC patients [fold change] or biomarker was positively correlated with EC risk [OR values]; ↓, biomarker was down‐regulated in EC patients [fold change] or biomarker was negatively correlated with EC risk [OR values].

Abbreviations: AA, arachidonic acid; AC, acylcarnitine; BC, breast cancer; COX, cyclooxygenase; DGLA, dihomo‐γ‐linolenic acid; DI, direct injection; E1, estrone; E2, oestradiol; E1‐S, estrone‐sulfate; EC, endometrial cancer; ESI, electrospray ionization; FIA, flow injection; GC, gas chromatography; GPL, glycerophospholipid; HR MAS, high‐resolution magic angle spinning; HPLC, high‐performance liquid chromatography; LA, linoleic acid; LC, liquid chromatography; LOX, lipoxygenase; LPC, lysophosphatidylcholine; MS, mass spectrometry; MS/MS, tandem mass spectrometry; NA, not available; NMR, nuclear magnetic resonance; OR, odds ratio; PC, Phosphatidylcholine; SD, standard deviation; TQ, triple quadruple; UPLC, ultra‐performance liquid chromatography; UPLC‐Q‐TOF/MS, ultra‐performance liquid chromatography to quadrupole time‐of‐flight mass spectrometry.

^a^ Data are expressed as median ± SD.

### 3.3 Assessment of research quality

The 30 included studies were assessed for quality using the NOS. The results are shown in Table S2, and the quality scores of the included studies ranged from 4 to 8, with a mean score of 6.0 ± 1.1 (mean ± SD). A total of 19 studies had a quality score of ≥6 and were considered to be of high quality.

### 3.4 Meta‐analysis of biomarkers expression in EC

Among the 30 studies, some studies did not report enough data for further analysis. For example, only the fold change of metabolites between EC and the control population were reported, but the concentration measurement of metabolites was not reported. In addition, there was also study of low quality. Therefore, we only included 8 studies with good quality that could extract or convert enough metabolite concentration data for meta‐analysis.

**FIGURE 2:**
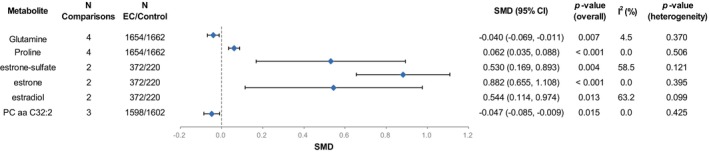
Forest plot of the changes in levels of biomarkers associated with endometrial cancer. PC, phosphatidylcholine; SMD, standardized mean difference.

Overall, we extracted SMD and SE values of 350 metabolites from eight studies. We examined the overlap of EC metabolites identified in eight studies using the upset plot, with a total of 135 biomarkers reported in at least two studies, and therefore performed a meta‐analysis (Figure S1). The results showed that among all 135 metabolites, a total of six metabolites shared significant differences between the EC and the control groups (Table S3, Figure 2). In EC, levels of three estrogen metabolites including estrone‐sulfate (SMD [95% CI] = 0.530 [0.169, 0.893], *p*‐value = 0.004), estrone (SMD [95% CI] = 0.882 [0.655, 1.108], *p*‐value <0.001) and estradiol (SMD [95% CI] = 0.544 [0.114, 0.974], *p*‐value = 0.013), and proline (SMD [95% CI] = 0.062 [0.035, 0.088], *p*‐value <0.001) were higher, and levels of glutamine (SMD [95% CI] = −0.040 [−0.069, −0.011], *p*‐value = 0.007) and PC aa C32:2 (SMD [95% CI] = −0.047 [−0.085, −0.009], *p*‐value = 0.015) were lower (Figure S2). Among them, the heterogeneity of the two estrogen metabolites (estrone‐sulfate [*I* ^2^ = 58.5%, *p*‐value = 0.121] and estradiol [*I* ^2^ = 63.2%, *p*‐value = 0.099]) was slightly higher, while the other metabolites had no significant heterogeneity.

### 3.5 Meta‐analysis of metabolic pathway expression in EC

As shown in the upset plot, although there were a large number of shared biomarkers in the eight studies, the sample size of these overlapping biomarkers was limited and may not yield better statistical power. Therefore, we assigned primary metabolic pathways to each biomarker based on the HMDB and KEGG databases and explored the unique EC pathway characteristics by analysing the metabolic pathways of biomarkers between the EC and the control groups (Table S4).^48^

**FIGURE 3:**
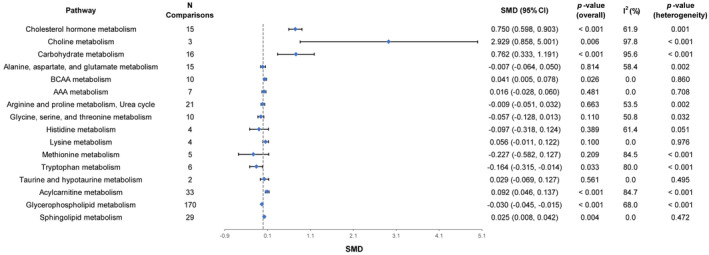
Forest plot of the changes in levels of metabolic pathways associated with endometrial cancer. AAA, aromatic amino acid; BCAA, branched‐chain amino acid; SMD, standardized mean difference.

In the eight included studies, a total of 350 EC biomarkers were enriched in 16 metabolic pathways for the identification of differential metabolic pathways in EC. We found that there were significant differences in eight metabolic pathways between EC and control groups. Compared with the control group, cholesterol hormone metabolism (SMD [95% CI] = 0.750 [0.598, 0.903], *p*‐value <0.001), choline metabolism (SMD [95% CI] = 2.929 [0.858, 5.001], *p*‐value = 0.006), carbohydrate metabolism (SMD [95% CI] = 0.762 [0.333, 1.191], *p*‐value <0.001), branched‐chain amino acid (BCAA) metabolism (SMD [95% CI] = 0.041 [0.005, 0.078], *p*‐value = 0.026), acylcarnitine metabolism (SMD [95% CI] = 0.092 [0.046, 0.137], *p*‐value <0.001), and sphingolipid metabolism (SMD [95% CI] = 0.025 [0.008, 0.042], *p*‐value = 0.004) were significantly up‐regulated in EC, tryptophan metabolism (SMD [95% CI] = −0.164 [−0.315, −0.014], *p*‐value = 0.033) and glycerophospholipid metabolism (SMD [95% CI] = −0.030 [−0.045, −0.015], *p*‐value <0.001) were significantly down‐regulated in EC, and no significant differences were found for the remaining metabolic pathways (Figure 3, Table S5). Forest plots of these metabolic pathways are shown in Figure S3. In these significantly different metabolic pathways, the heterogeneity test showed that, with the exception of BCAA metabolism (*I* ^2^ = 0.0%, *p*‐value = 0.860) and sphingolipid metabolism (*I* ^2^ = 0.0%, *p*‐value = 0.472), the other metabolic pathways were highly heterogeneous.

### 3.6 Publication bias and correction

Assessing publication bias is a critical step in objectively presenting the results of a meta‐analysis. The results of Egger's test are showed in Table S6. Carbohydrate metabolism, BCAA metabolism, arginine and proline metabolism, urea cycle, acylcarnitine metabolism and glycerophospholipid metabolism with more than 10 data points had potential publication bias (*p*‐values <0.10). Therefore, the trim and fill method was used to correct for publication bias in these five metabolic pathways. After applying the trim and fill test, the trend for significant up‐regulation of acylcarnitine metabolism in EC was slightly weakened (SMD [95% CI] = 0.077 [0.029, 0.126]) and it was speculated that there might be a small‐study bias. The pooled SMD for the remaining metabolic pathways was unchanged, indicating robust results.

### 3.7 Subgroup analysis of biological types

Subgroup analysis based on the type of biological sample showed that compared with the control group, level of carbohydrate metabolism in EC maintained a significant up‐regulated trend in both the cervicovaginal fluid and serum subgroups, level of BCAA metabolism and sphingolipid metabolism maintained significant up‐regulated trends in the plasma subgroup, level of acylcarnitine metabolism and glycerophospholipid metabolism maintained significant up‐regulated and down‐regulated trends in the serum subgroup, respectively (Table 2, Figure S4). In addition, levels of carbohydrate metabolism, acylcarnitine metabolism and glycerophospholipid metabolism were significantly different among the different subgroups (*p*‐values <0.05), suggesting that there was an interaction between biological sample type and the combined effect size of these metabolic pathways, which might be the potential source of their heterogeneity.

**TABLE 2:** Subgroup analysis, according to biological sample type.

Pathways	Biological sample type	No. of comparisons	SMD (95% CI)	*p*‐value (overall)	*I* ^2^ (%)	*p*‐value (heterogeneity)	*p*‐value (sub)
Carbohydrate metabolism	Cervicovaginal fluid	4	10.401 (0.855, 19.947)	0.033	99.0	<0.001	<0.01
	Serum	11	0.178 (0.013, 0.343)	0.035	55.2	0.014	
	Plasma	——	——	——	——	——	
Alanine, aspartate and glutamate metabolism	Cervicovaginal fluid	2	−0.342 (−0.732, 0.047)	0.085	0.0	0.876	0.23
	Serum	8	−0.020 (−0.241, 0.201)	0.860	65.8	0.005	
	Plasma	5	−0.001 (−0.046, 0.043)	0.961	62.4	0.031	
BCAA metabolism	Cervicovaginal fluid	——	——	——	——	——	0.11
	Serum	6	−0.048 (−0.196, 0.101)	0.529	0.0	0.999	
	Plasma	3	0.049 (0.012, 0.087)	0.010	0.0	0.96	
Arginine and proline metabolism, Urea cycle	Cervicovaginal fluid	——	——	——	——	——	0.24
	Serum	13	−0.081 (−0.217, 0.055)	0.242	40.2	0.066	
	Plasma	8	0.004 (−0.034, 0.041)	0.843	64.4	0.006	
Glycine, serine and threonine metabolism	Cervicovaginal fluid	——	——	——	——	——	0.42
	Serum	7	−0.122 (−0.321, 0.077)	0.229	51.8	0.053	
	Plasma	3	−0.038 (−0.091, 0.015)	0.165	55.3	0.107	
Acylcarnitine metabolism	Cervicovaginal fluid	——	——	——	——	——	<0.01
	Serum	18	0.437 (0.201, 0.672)	< 0.001	85.3	< 0.001	
	Plasma	15	0.005 (−0.011, 0.021)	0.535	17.9	0.253	
Glycerophospholipid metabolism	Cervicovaginal fluid	——	——	——	——	——	<0.01
	Serum	85	−0.249 (−0.302, −0.196)	< 0.001	43.2	< 0.001	
	Plasma	84	0.003 (−0.007, 0.012)	0.551	40.3	< 0.001	
Sphingolipid metabolism	Cervicovaginal fluid	——	——	——	——	——	0.33
	Serum	15	−0.027 (−0.133, 0.079)	0.616	20.5	0.226	
	Plasma	14	0.027 (0.009, 0.044)	0.003	0.0	0.769	

*Note*: *p*‐value (sub)—*p*‐value for the difference in coefficient between subgroups.

Abbreviations: BCAA, branched‐chain amino acid; CI, confidence intervals; SMD, standardized mean difference.

### 3.8 Subgroup analysis of control group types

Subgroup analysis based on the type of control group showed that compared to the control group, the levels of carbohydrate metabolism in EC maintained a trend of significant up‐regulation in both the healthy control and non‐cancer control subgroups; BCAA metabolism and sphingolipid metabolism were maintained at significantly up‐regulated levels in the non‐cancer control subgroup; acylcarnitine metabolism and glycerophospholipid metabolism were maintained at significantly up‐regulated and down‐regulated levels in the healthy control subgroup, respectively (Table 3, Figure S5). In addition, levels of carbohydrate metabolism, acylcarnitine metabolism and glycerophospholipid metabolism were significantly different among the different subgroups (*p*‐values <0.05), suggesting that the type of control group might be a potential source of heterogeneity in these metabolic pathways.

**TABLE 3:** Subgroup analysis, according to control type.

Pathways	Control type	No. of comparisons	SMD (95% CI)	*p*‐value (overall)	*I* ^2^ (%)	*p*‐value (heterogeneity)	*p*‐value (sub)
Carbohydrate metabolism	Health control	11	0.178 (0.013, 0.343)	0.035	55.2	0.014	<0.01
	Non‐cancer control	5	7.665 (4.254, 11.075)	<0.001	98.7	<0.001	
Alanine, aspartate and glutamate metabolism	Health control	8	−0.020 (−0.241, 0.201)	0.860	65.8	0.005	0.90
	Non‐cancer control	7	−0.005 (−0.050, 0.040)	0.823	54.4	0.041	
BCAA metabolism	Health control	6	−0.048 (−0.196, 0.101)	0.529	0.0	0.999	0.23
	Non‐cancer control	4	0.047 (0.009, 0.085)	0.015	1.7	0.384	
Arginine and proline metabolism, Urea cycle	Health control	12	−0.054 (−0.174, 0.066)	0.375	22.9	0.218	0.41
	Non‐cancer control	9	−0.001 (−0.043, 0.042)	0.968	70.0	0.001	
Glycine, serine and threonine metabolism	Health control	7	−0.122 (−0.321, 0.077)	0.229	51.8	0.053	0.42
	Non‐cancer control	3	−0.038 (−0.091, 0.015)	0.165	55.3	0.107	
Acylcarnitine metabolism	Health control	17	0.421 (0.179, 0.664)	0.001	86.0	<0.001	<0.01
	Non‐cancer control	16	0.006 (−0.011, 0.024)	0.476	31.2	0.113	
Glycerophospholipid metabolism	Health control	85	−0.249 (−0.302, −0.196)	< 0.001	43.2	<0.001	<0.01
	Non‐cancer control	85	0.003 (−0.009, 0.015)	0.602	64.5	<0.001	
Sphingolipid metabolism	Health control	15	−0.027 (−0.133, 0.079)	0.616	20.5	0.226	0.33
	Non‐cancer control	14	0.027 (0.009, 0.044)	0.003	0.0	0.769	

*Note*: *p*‐value (sub)—*p*‐value for the difference in coefficient between subgroups.

Abbreviations: BCAA, branched‐chain amino acid; CI, confidence intervals; SMD, standardized mean difference.

### 3.9 Sensitivity analysis of data conversion

Considering that the SMD values and their SE values in three studies were converted from the OR values and their 95% CIs, we removed this part of the data and performed sensitivity analysis for the studies that were not data‐transformed (Table S7).^17^, ^19^, ^45^ The results showed that after excluding the transformed data, cholesterol hormone metabolism, carbohydrate metabolism and acylcarnitine metabolism maintained significant up‐regulated trends, and glycerophospholipid metabolism maintained a significant down‐regulated trend. Notably, BCAA metabolism and sphingolipid metabolism were changed from significantly up‐regulated to non‐significant. Thus, it is suggested that the heterogeneity was mainly caused by data transformation.

### 3.10 Meta‐regression analysis of sample size

**FIGURE 4:**
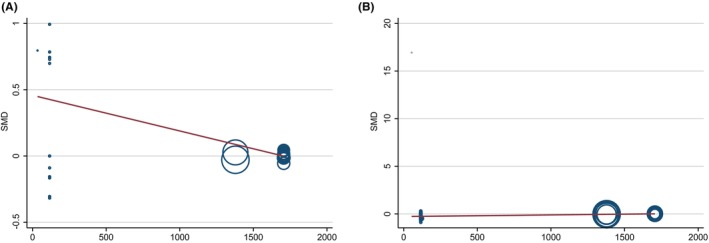
Meta‐regression analysis between sample size and effect of metabolic pathways. (A) Acylcarnitine metabolism; (B) Glycerophospholipid metabolism. SMD, standardized mean difference.

Considering the potential impact of sample size on CIs and heterogeneity, we used meta‐regression analysis to identify whether there was a significant correlation between sample size and effect size. The results showed that there was a significant negative correlation between sample size and the effect size of acylcarnitine metabolism (regression coefficient = −0.00027, *p*‐value = 0.001), but a significant positive correlation with the effect size of glycerophospholipid metabolism (regression coefficient = 0.00017, *p*‐value <0.001). It is suggesting that for studies with a large sample size, the level of acylcarnitine metabolism might be lower, and the level of glycerophospholipid metabolism might be higher (Table S8, Figure 4).

### 3.11 GRADE quality of evidence

Using the GRADE framework, we judged the overall quality of evidence for the outcome of 67 biomarkers to be low and 68 biomarkers to be very low due to the possible presence of inconsistency or imprecision (Table S9). The overall quality of evidence for the outcome of eight metabolic pathways were judged to be low, and eight metabolic pathways were judged to be very low due to the possible presence of inconsistency, imprecision or publication bias (Table S10).

## 4 DISCUSSION

### 4.1 Main findings

In this review, we integrated 350 metabolites from 4,006 EC and control populations from eight studies to reveal EC metabolic characteristics changes. We found that in terms of biomarkers, compared with the control group, the levels of estrone and proline were significantly higher in EC, while the levels of glutamine and PC aa C32:2 were significantly lower in EC, with low heterogeneity. In terms of metabolic pathways, the levels of BCAA metabolism and sphingolipid metabolism were significantly higher in EC, with low heterogeneity.

### 4.2 Discussion on the source of heterogeneity

Our meta‐analysis revealed the heterogeneity of glycerophospholipid metabolism and acylcarnitine metabolism was mainly affected by the biological sample type, control group type and sample size. The heterogeneity of carbohydrate metabolism was mainly caused by the difference of biological sample type and control group type. The heterogeneity of BCAA metabolism and sphingolipid metabolism was mainly affected by data conversion. Therefore, the effects of the above factors should be considered in future studies on biomarkers of EC to validate changes in related metabolic pathways.

However, we speculate that there are other factors that are difficult to determine that may affect the results of the analysis. For example, metabolomics methodological differences, such as analytical platforms and quantification methods for metabolite identification, may also be possible sources of heterogeneity. As common analysis platforms for metabolomics, MS and NMR have been widely used for the characterization of disease metabolomics and the identification of biomarkers.^49^ Among them, MS has become an important analytical tool in metabolomics studies given its higher sensitivity compared to NMR, which allows the determination of polar metabolites in a wider range.^50^, ^51^ Similarly, of the eight studies we included in the meta‐analysis, the vast majority used the MS platform, with only one study choosing the NMR platform. In terms of metabolite annotation and quantification, non‐targeted metabolomics methods have high metabolite coverage in full scan mode, but their low quantitative accuracy and sensitivity limit their application.^52^, ^53^ By contrast, targeted metabolomics methods can separate the target metabolites and eliminate a large number of signals in the background matrix through multi‐reaction monitoring mode, resulting in high sensitivity and selectivity, making targeted metabolomics have significant advantages in the quantification of metabolites.^54^, ^55^ Targeted methods were used in metabolomics studies in the eight studies we included, which makes our findings generalizable and feasible. Notably, we still believe that it is necessary for researchers to follow strict experimental design and procedures in metabolite quantification, so as to obtain replicable results between different studies.^56^, ^57^

In addition, demographic and clinical factors, including race, menopausal status, lifestyle and use of hormone replacement therapy, may have metabolic effects on EC patients.^58^, ^59^, ^60^, ^61^ Since no specific data were presented or measured for the clinical characteristics of individual patients in the included observational studies, potential confounders could not be adjusted for in our analysis. For example, changes in postmenopausal status may cause decreased estradiol and increased androgen levels, and further lead to various metabolic disorders, including lipid metabolism disorders.^62^, ^63^ Since the menopausal status of EC and control population was not defined in some of the included studies, we were unable to explain the differences due to confounding factors such as menopausal status. With regard to the source and collection of samples, we were unable to determine whether EC patients included in the study had and adjusted for potential comorbidities. Clinically, EC patients are often accompanied by other gynaecological benign diseases (such as endometriosis) and malignant diseases (such as ovarian cancer), and their metabolomics characteristics may be different from those of the control population.^64^, ^65^ In our analysis, we were also unable to explain the differences due to our patient's comorbidities because the exclusion of the patient from the comorbidities was not explicitly stated in the included studies. In addition, these factors may also be a source of potential heterogeneity due to different diagnostic criteria or different methods of sample collection, processing and storage in EC patients in the study.

### 4.3 Mechanism of related metabolic pathways in EC

It is well known that the imbalance caused by the increase of estradiol and/or the decrease of progesterone role can lead to abnormal hyperplasia of endometrium, which eventually leads to cancer.^66^ Sex hormones play a key role in the occurrence and development of EC.^67^ Studies have shown that excessive endogenous estrogen may directly stimulate the growth of endometrium by activating estrogen receptors α and play a carcinogenic role by activating insulin‐like growth factor (IGF) receptor 1 and epidermal growth factor receptor, thereby activating the downstream PI3K‐AKT–mTOR pathway.^68^, ^69^, ^70^

Furthermore, in amino acid metabolism, amino acid levels in healthy individuals are usually controlled within a certain range by the homeostatic function of the organism.^71^ Studies have shown that various amino acid levels of patients with different types of gynaecological cancer may be changed.^72^, ^73^ There is increasing evidence that bioactive sphingolipids play a key role in the occurrence and development of cancer. Studies have shown that, compared with the health group, the content of a variety of sphingolipids, including sphinganine and ceramide, in the EC endometrial tissue is significantly increased, which is related to the increased activities of serine palmitoyltransferase and sphingosine kinase 1.^74^ Therefore, increased expression of BCAA metabolism and sphingolipid metabolism is an important metabolic feature in EC.

In addition, in the glycerophospholipid metabolism, phosphatidylcholine (PC) is synthesized mainly through the cytidine diphosphate (CDP)‐choline pathway in mammalian cells and can be catabolized to produce lysophosphatidylcholine (LPC) with pro‐tumorigenic effects.^75^ PC can interconvert with LPC in the presence of two metabolic enzymes, LPC acyltransferase and phospholipase A2.^76^ Studies have shown that metabolic disorders of PC and LPC, as important markers of cancer, are found to be changed in a variety of gynaecological cancers.^77^, ^78^

Acylcarnitine is an ester of carnitine and fatty acids that plays an important role in the transport of fatty acids across the mitochondrial membrane, thereby participating in the regulation of the balance of intracellular glucose and lipid metabolism.^79^ Studies have shown that the increased circulation level of acylcarnitine occurs in different gynaecological cancers, including EC. Some researchers have speculated that the down‐regulation of carnitine palmitoyltransferase 2 is the main reason for the accumulation of acylcarnitine in cancer, which may further lead to impaired β‐oxidation and more energy consumption.^80^

Studies have shown that carbohydrate metabolism disorders, as a risk factor for EC, are more common in women with endometrial lesions.^81^ Studies using oral glucose tolerance test, blood glucose and serum insulin levels as indicators to assess the status of carbohydrate metabolism have found that the degree of carbohydrate intolerance in EC is higher than that in the control group.^82^ Studies have also shown that, compared with healthy control subjects, patients with EC have higher average fasting blood glucose and insulin levels, and in addition to carbohydrate metabolism, the insulin‐regulated circulating IGF system in postmenopausal women with EC is also changed.^83^

### 4.4 Strengths and limitations

The advantages of this study mainly include the following three points. First, we performed the first systematic review and meta‐analysis of the metabolomic signature of EC, providing strong evidence so far about biomarkers and metabolic pathway changes in EC based on metabolomics. Second, our findings were based on a systematic and comprehensive literature search and screening strategy that minimizes the possibility of missing relevant published studies. Finally, the vast majority of metabolic pathways included in this study were found in a relatively large sample (> 1,000 participants), and the evaluation results based on the large sample size were more reliable than the small sample size.

However, our study still has some limitations. First, due to the limited number of single metabolites reported in different studies, their sample size is not enough to achieve better statistical power. Therefore, we performed a meta‐analysis of metabolic pathways corresponding to any specific biomarker to explore changes in EC metabolic pathways. Although this idea can provide a reference for the identification of metabolic characteristics of diseases, we still need to verify the feasibility of specific metabolites in key metabolic pathways as biomarkers of EC, so as to make up the blank of disease biomarkers.^48^ Second, due to the insufficient data in many EC metabolomics studies, resulting in limited quantity of evidence for most individual biomarkers. Thus, further more studies with more patients are required to validate our findings. Third, due to the existence of differences in some known heterogeneity factors of this study (such as biological sample type, control type, data conversion and sample size) and in some unknown or unmeasured factors (such as enrolled populations' centre settings, complications and confounding factors [race, menopausal status, lifestyle, and the use of hormone replacement therapy]), there was significant heterogeneity in some research results. Therefore, the current results should be interpreted with prudence, and future studies with higher statistical power to assess sources of heterogeneity need to account for these factors. Fourth, due to the presence of small‐study effects, there was potential publication bias in some metabolic pathways (especially acylcarnitine metabolism). Therefore, these research findings should be treated with caution. Fifth, because all available evidence included in this meta‐analysis was from observational studies, the overall evidence quality of EC biomarkers and metabolic pathways was low or very low, indicating the possible bias and uncertainty of the results. Thus, further high‐quality studies are needed to confirm these findings.

## 5 CONCLUSION

In conclusion, this meta‐analysis integrated 350 metabolites from 4,006 EC and control populations from eight studies, and performed a quantitative meta‐analysis of 135 biomarkers and 16 metabolic pathways. The results of meta‐analysis highlight the important role of estrone, proline, glutamine and PC aa C32:2 in biomarkers, and BCAA metabolism and sphingolipid metabolism in metabolic pathways in EC early screening and diagnosis. Our findings suggest that clinicians must pay attention to the changes of above four biomarkers and two metabolic pathways of human EC, which will help to assist the existing diagnosis and detection methods of EC and realize the accurate screening and diagnosis of clinical EC.

## AUTHOR CONTRIBUTIONS

**Xingxu Yan:** Conceptualization (lead); data curation (equal); formal analysis (equal); writing – original draft (lead). **Shan Zhao:** Data curation (equal); formal analysis (equal); investigation (equal); writing – original draft (supporting). **Junjie He:** Conceptualization (supporting); data curation (equal); formal analysis (supporting); investigation (supporting); writing – original draft (supporting). **Guijiang Sun:** Methodology (equal); resources (equal); supervision (equal). **Fang Wang:** Methodology (equal); resources (equal); software (equal). **Haoran Ding:** Formal analysis (equal); methodology (equal); software (equal). **Wenqing Zhang:** Methodology (equal); software (equal); validation (equal). **Wenxiu Qian:** Methodology (equal); validation (equal); visualization (equal). **Xiaomeng Li:** Validation (equal); visualization (equal). **Yuyan Wang:** Conceptualization (equal); writing – review and editing (equal). **Yubo Li:** Conceptualization (equal); writing – review and editing (lead).

## FUNDING INFORMATION

This work was supported by the Tianjin Talent Development Special Support Project for High Level Innovation and Entrepreneurship and the National Talent Project of ‘Youth Qihuang Scholars’.

## CONFLICT OF INTEREST STATEMENT

The authors declare that they have no competing interests.

## Supporting information


Data S1.

## Data Availability

All data generated or analysed during this study are included in this published article [and its supplementary information files]. The protocol of the study is available on PROSPERO. The full texts of all included studies were retrieved from the online databases (PubMed, EMBASE, Cochrane Library and Web of Science) and other source databases (ClinicalTrials.gov, bioRxiv, medRxiv and OpenGrey). The data of this systematic review and meta‐analyses are all pubic and available from these online databases.

## APPENDIX A Preferred reporting items for systematic reviews and meta‐analysis (PRISMA) checklist

### A.1

**Table:**

Section and Topic	Item #	Checklist item	Location where item is reported
**TITLE**			
Title	1	Identify the report as a systematic review.	Page 1
**ABSTRACT**			
Abstract	2	See the PRISMA 2020 for Abstracts checklist.	Page 2
**INTRODUCTION**			
Rationale	3	Describe the rationale for the review in the context of existing knowledge.	Page 3–4
Objectives	4	Provide an explicit statement of the objective(s) or question(s) the review addresses.	Page 4–5
**METHODS**			
Eligibility criteria	5	Specify the inclusion and exclusion criteria for the review and how studies were grouped for the syntheses.	Page 5–6
Information sources	6	Specify all databases, registers, websites, organizations, reference lists and other sources searched or consulted to identify studies. Specify the date when each source was last searched or consulted.	Page 5
Search strategy	7	Present the full search strategies for all databases, registers and websites, including any filters and limits used.	Table S1
Selection process	8	Specify the methods used to decide whether a study met the inclusion criteria of the review, including how many reviewers screened each record and each report retrieved, whether they worked independently, and if applicable, details of automation tools used in the process.	Page 5
Data collection process	9	Specify the methods used to collect data from reports, including how many reviewers collected data from each report, whether they worked independently, any processes for obtaining or confirming data from study investigators, and if applicable, details of automation tools used in the process.	Page 6
Data items	10a	List and define all outcomes for which data were sought. Specify whether all results that were compatible with each outcome domain in each study were sought (e.g., for all measures, time points, analyses), and if not, the methods used to decide which results to collect.	Page 6
	10b	List and define all other variables for which data were sought (e.g., participant and intervention characteristics, funding sources). Describe any assumptions made about any missing or unclear information.	Page 6
Study risk of bias assessment	11	Specify the methods used to assess risk of bias in the included studies, including details of the tool(s) used, how many reviewers assessed each study and whether they worked independently, and if applicable, details of automation tools used in the process.	Page 6
Effect measures	12	Specify for each outcome the effect measure(s) (e.g., risk ratio, mean difference) used in the synthesis or presentation of results.	Page 7
Synthesis methods	13a	Describe the processes used to decide which studies were eligible for each synthesis (e.g., tabulating the study intervention characteristics and comparing against the planned groups for each synthesis (item #5)).	Page 7
	13b	Describe any methods required to prepare the data for presentation or synthesis, such as handling of missing summary statistics, or data conversions.	Page 7
	13c	Describe any methods used to tabulate or visually display results of individual studies and syntheses.	Page 7
	13d	Describe any methods used to synthesize results and provide a rationale for the choice(s). If meta‐analysis was performed, describe the model(s), method(s) to identify the presence and extent of statistical heterogeneity, and software package(s) used.	Page 7
	13e	Describe any methods used to explore possible causes of heterogeneity among study results (e.g., subgroup analysis, meta‐regression).	Page 7–8
	13f	Describe any sensitivity analyses conducted to assess robustness of the synthesized results.	Page 7–8
Reporting bias assessment	14	Describe any methods used to assess risk of bias due to missing results in a synthesis (arising from reporting biases).	Page 7
Certainty assessment	15	Describe any methods used to assess certainty (or confidence) in the body of evidence for an outcome.	Page 8
**RESULTS**			
Study selection	16a	Describe the results of the search and selection process, from the number of records identified in the search to the number of studies included in the review, ideally using a flow diagram.	Page 8, Figure 1
	16b	Cite studies that might appear to meet the inclusion criteria, but which were excluded, and explain why they were excluded.	Page 8
Study characteristics	17	Cite each included study and present its characteristics.	Page 8–9, Table 1
Risk of bias in studies	18	Present assessments of risk of bias for each included study.	Page 9, Table S2
Results of individual studies	19	For all outcomes, present, for each study: (a) summary statistics for each group (where appropriate) and (b) an effect estimate and its precision (e.g., confidence/credible interval), ideally using structured tables or plots.	Figure S2‐S3
Results of syntheses	20a	For each synthesis, briefly summarize the characteristics and risk of bias among contributing studies.	Page 9–11
	20b	Present results of all statistical syntheses conducted. If meta‐analysis was done, present for each the summary estimate and its precision (e.g., confidence/credible interval) and measures of statistical heterogeneity. If comparing groups, describe the direction of the effect.	Page 10–11, Figure 2–3, Table S3, S5
	20c	Present results of all investigations of possible causes of heterogeneity among study results.	Page 11–13, Table 2–3, Figure 4, Table S8, Figure S4‐S5
	20d	Present results of all sensitivity analyses conducted to assess the robustness of the synthesized results.	Page 12–13, Table S7
Reporting biases	21	Present assessments of risk of bias due to missing results (arising from reporting biases) for each synthesis assessed.	Page 11, Table S6
Certainty of evidence	22	Present assessments of certainty (or confidence) in the body of evidence for each outcome assessed.	Page 13, Table S9‐S10
**DISCUSSION**			
Discussion	23a	Provide a general interpretation of the results in the context of other evidence.	Page 14
	23b	Discuss any limitations of the evidence included in the review.	Page 14–18
	23c	Discuss any limitations of the review processes used.	Page 14–18
	23d	Discuss implications of the results for practice, policy and future research.	Page 19
**OTHER INFORMATION**			
Registration and protocol	24a	Provide registration information for the review, including register name and registration number, or state that the review was not registered.	Page 5
	24b	Indicate where the review protocol can be accessed, or state that a protocol was not prepared.	Page 5
	24c	Describe and explain any amendments to information provided at registration or in the protocol.	NA
Support	25	Describe sources of financial or non‐financial support for the review, and the role of the funders or sponsors in the review.	Page 20
Competing interests	26	Declare any competing interests of review authors.	Page 20
Availability of data, code and other materials	27	Report which of the following are publicly available and where they can be found: template data collection forms; data extracted from included studies; data used for all analyses; analytic code; any other materials used in the review.	Page 19–20

Note: EC, endometrial cancer.
