# Supplementary material for: Metabolomics biomarkers and related pathways in endometrial cancer: A systematic review and meta‐analysis
Source: J Cell Mol Med. 2024 May 28;28(10):e18401. doi: 10.1111/jcmm.18401 (PMC11132341; doi:10.1111/jcmm.18401)
Supplement: Supplementary file 1 — Data S1. [file JCMM-28-e18401-s001.docx]

**Supplementary Materials**

**Metabolomics Biomarkers and Related Pathways in Endometrial Cancer: A Systematic Review and Meta-analysis**

Xingxu Yan, Shan Zhao, Junjie He, Guijiang Sun, Fang Wang, Haoran Ding, Wenqing Zhang, Wenxiu Qian, Xiaomeng Li, Yuyan Wang, Yubo Li

**Table of Contents**

1. **Supplementary Methods:** assessment of evidence certainty.

2. **Table S1:** Literature search strategy of the study.

3. **Table S2:** The risk of bias assessment of included studies using a 9-point Newcastle–Ottawa Scale.

4. **Table S3**: Meta-analysis results of biomarkers of endometrial cancer.

5. **Table S4:** 181 metabolites and the corresponding main metabolic pathways.

6. **Table S5:** Effect size of metabolic pathways between endometrial cancer patients and controls.

7. **Table S6:** Publication bias and correction of metabolic pathways of endometrial cancer.

8. **Table S7:** Sensitivity analysis, according to data conversion.

9. **Table S8:** Meta-regression analysis of sample size.

10. **Table S9:** GRADE assessment for all outcomes of endometrial cancer biomarkers evaluated in this meta-analysis.

11. **Table S10:** GRADE assessment for all outcomes of endometrial cancer metabolic pathways evaluated in this meta-analysis.

12. **Figure S1:** Upset plot illustrating the overlap of endometrial cancer metabolites identified across studies.

13. **Figure S2:** Forest plots assessing changes in the levels of biomarkers associated with endometrial cancer.

14. **Figure S3:** Forest plots assessing changes in the levels of metabolic pathways associated with endometrial cancer.

15. **Figure S4:** Forest plots of biological sample type subgroup analysis of endometrial cancer-associated metabolic pathways.

16. **Figure S5:** Forest plots of control group type subgroup analysis of endometrial cancer-associated metabolic pathways.

17. **Supplementary References:** supplementary references for included articles.

**Supplementary Methods: assessment of evidence certainty.**

Evidence extracted from observational studies were defaulted to ‘low’ certainty and were downgraded or upgraded based on pre-specified criteria. Criteria to downgrade included: (1) rias of risk: high risk of bias in more than 75% of the included studies [studies with high risk of bias were categorized with scores ranging from 1-4 base on the Newcastle-Ottawa Scale]; (2) inconsistency: wide variance of point estimates across studies, minimal or no overlap of confidence intervals, and statistical criteria including tests of heterogeneity [*p*-value < 0.05] and I^2^ [I^2^ > 50%]; (3) indirectness: presence of factors that limit the generalizability of the results [especially the differences in population]; (4) inconsistency: small number of participants [n < 400] or wide confidence intervals around the estimate of the effect; (5) publication bias: small-study effects [if 10 or more comparisons]. Upgraded criteria included: (1) large magnitude of an effect; (2) dose-response gradient; (3) effect of plausible residual confounding. The quality of evidence based on the GRADE framework was assessed independently by two reviewers (X.Y. and J.H.), disagreements were discussed with a third reviewer (Y.L.).

Each meta-analysis outcome assessed was determined to be of very low, low, moderate or high certainty. (1) high quality: very confident that the true effect lies close to that of the estimate of the effect; (2) moderate quality: moderately confident in the effect estimate, and the true effect is likely to be close to the estimate of the effect, but there is a possibility that it is substantially different; (3) low quality: confidence in the effect estimate is limited, and the true effect could be substantially different from the estimate of the effect; (4) very low quality: very little confidence in the effect estimate, and the true effect is likely to be substantially different from the estimate of effect.

**Table S1: Literature search strategy of the study.**

| **PubMed** | |
| --- | --- |
| #1 | Endometrial Neoplasms [mesh] OR (Neoplasm* [all] AND "Endometrial" [all]) OR (Carcinoma* [all] AND "Endometrial" [all]) OR (Cancer* [all] AND "Endometrial" [all]) OR (Cancer* [all] AND "Endometrium" [all]) OR Endometrium Carcinoma* [all] OR "Carcinoma of Endometrium" [all] |
| #2 | Metabolomics [mesh] OR Metabolomic* [all] OR Metabonomic* [all] OR Metabolome [mesh] OR Metabolome* [all] OR (Profile* [all] AND "Metabolic" [all]) OR Lipidomics [mesh] OR Lipidom* [all] |
| #3 | #1 AND #2 |
| **Embase** | |
| #1 | 'endometrium carcinoma'/exp OR (neoplasm* AND Endometrial) OR (carcinoma* AND Endometrial) OR (cancer* AND Endometrial) OR (cancer* AND Endometrium) OR 'endometrium carcinoma*' OR 'Carcinoma of Endometrium' |
| #2 | 'metabolomics'/exp OR metabolomic* OR metabonomic* OR 'metabolome'/exp OR metabolome* OR (profile* AND Metabolic) OR 'lipidomics'/exp OR lipidom* |
| #3 | #1 AND #2 |
| **Cochrane Library** | |
| #1 | MeSH descriptor: [Endometrial Neoplasms] explode all trees |
| #2 | MeSH descriptor: [Metabolomics] explode all trees |
| #3 | MeSH descriptor: [Metabolome] explode all trees |
| #4 | MeSH descriptor: [Lipidomics] explode all trees |
| #5 | (neoplasm* AND Endometrial) OR (carcinoma* AND Endometrial) OR (cancer* AND Endometrial) OR (cancer* AND Endometrium) OR (Endometrium NEXT carcinoma*) OR "Carcinoma of Endometrium" |
| #6 | metabolomic* OR metabonomic* OR metabolome* OR (profile* AND Metabolic) OR lipidom* |
| #7 | (#1 OR #5) AND (#2 OR #3 OR #4 OR #6) |
| **Web of Science** | |
| #1 | TS=("endometrial neoplasms") OR (ALL=(neoplasm*) AND ALL=("Endometrial")) OR (ALL=(carcinoma*) AND ALL=("Endometrial")) OR (ALL=(cancer*) AND ALL=("Endometrial")) OR (ALL=(cancer*) AND ALL=("Endometrium")) OR ALL=("endometrium carcinoma*") OR ALL=("Carcinoma of Endometrium") |
| #2 | TS=("metabolomics") OR ALL=(metabolomic*) OR ALL=(metabonomic*) OR TS=("metabolome") OR ALL=(metabolome*) OR (ALL=(profile*) AND ALL=("Metabolic")) OR TS=("lipidomics") OR ALL=(lipidom*) |
| #3 | #1 AND #2 |
| **ClinicalTrials.gov** | |
| #1 | (metabolomics OR lipidomics) AND endometrial cancer |
| **bioRxiv and medRxiv** | |
| #1 | (metabolomics OR lipidomics) AND endometrial cancer |
| **OpenGrey** | |
| #1 | (metabolomics OR lipidomics) AND endometrial cancer |

**Table S2:** The risk of bias assessment of included studies using a 9-point Newcastle–Ottawa Scale.

| **Reference** | **Selection (4)** ^†^ | **Comparability (2)** ^‡^ | **Exposure/ Outcome (3)** ^§^ | **Overall Quality Score** |
| --- | --- | --- | --- | --- |
| Bufa A, 2010 [1] | 1 | 2 | 1 | 4 |
| Audet-Delage Y, 2018 [2] | 3 | 1 | 2 | 6 |
| Skorupa A, 2021 [3] | 3 | 2 | 2 | 7 |
| Shi K, 2018 [4] | 4 | 1 | 2 | 7 |
| Audet-Delage Y, 2018 [5] | 4 | 1 | 2 | 7 |
| Kozar N, 2021 [6] | 4 | 2 | 2 | 8 |
| Cummings M, 2019 [7] | 3 | 0 | 2 | 5 |
| Shafiee MN, 2020 [8] | 4 | 0 | 2 | 6 |
| Altadill T, 2017 [9] | 3 | 1 | 2 | 6 |
| Njoku K, 2021 [10] | 3 | 1 | 2 | 6 |
| Cheng SC, 2019 [11] | 4 | 2 | 2 | 8 |
| Bahado-Singh RO, 2017 [12] | 2 | 1 | 2 | 5 |
| Troisi J, 2018 [13] | 4 | 1 | 2 | 7 |
| Jové M, 2016 [14] | 3 | 0 | 2 | 5 |
| Knific T, 2018 [15] | 3 | 2 | 1 | 6 |
| Schuhn A, 2022 [16] | 4 | 0 | 1 | 5 |
| Dossus L, 2021 [17] | 4 | 2 | 2 | 8 |
| Shao X, 2016 [18] | 2 | 2 | 2 | 6 |
| Yan X, 2022 [19] | 4 | 0 | 2 | 6 |
| Yi R, 2022 [20] | 2 | 2 | 1 | 5 |
| Lépine J, 2010 [21] | 2 | 1 | 2 | 5 |
| Zhao SS, 2022 [22] | 4 | 0 | 1 | 5 |
| Hishinuma E, 2023 [23] | 1 | 1 | 2 | 4 |
| Boyd AE, 2023 [24] | 3 | 2 | 2 | 7 |
| Arda Düz S, 2022 [25] | 3 | 2 | 2 | 7 |
| Troisi J, 2022 [26] | 4 | 0 | 1 | 5 |
| Gu M, 2021 [27] | 3 | 0 | 2 | 5 |
| Breeur M, 2022 [28] | 4 | 1 | 2 | 7 |
| Hao C, 2023 [29] | 4 | 0 | 2 | 6 |
| Cheng F, 2023 [30] | 3 | 1 | 2 | 6 |

^†^: Based on the following factors, each study can get a maximum of four points. For case-control studies, adequacy of case definition, representativeness of cases, selection of controls, and definition of controls. For cohort studies, representativeness of exposed cohort, selection of non-exposed cohort, ascertainment of exposure, and demonstration that outcome of interest was not present at start of study.

^‡^: Based on the following factors, each study can get a maximum of two points. For case-control studies, based on the comparability between cases and controls. For cohort studies, based on the comparability between the exposed and non-exposed group. One point was awarded if the study matched for age, and the other point was awarded if the study matched menopausal status.

^§^: Based on the following factors, each study can get a maximum of three points. For case-control studies, ascertainment of exposure, same method of ascertainment for cases and controls, and same non-response rate. For cohort studies, assessment of outcome, duration of follow-up for outcome, and adequacy of follow-up of cohorts.

**Table S3: Meta-analysis results of biomarkers of endometrial cancer.**

| **Metabolite** | **N Comparisons** | **N EC/Control** | **SMD (95% CI)** | ***p*-value (overall)** | **I^2^ (%)** | ***p*-value (heterogeneity)** |
| --- | --- | --- | --- | --- | --- | --- |
| Hexose | 2 | 909/913 | 0.095 (-0.247, 0.436) | 0.587 | 71.8 | 0.060 |
| Glutamine | 4 | 1654/1662 | -0.040 (-0.069, -0.011) | 0.007 | 4.5 | 0.370 |
| Glutamate | 2 | 909/913 | 0.271 (-0.201, 0.742) | 0.261 | 83.6 | 0.014 |
| Glycine | 3 | 965/973 | -0.084 (-0.168, -0.000) | 0.050 | 5.6 | 0.347 |
| Serine | 2 | 909/913 | -0.134 (-0.364, 0.097) | 0.255 | 49.5 | 0.159 |
| Aspartate | 2 | 77/93 | -0.074 (-0.392, 0.245) | 0.650 | 6.8 | 0.300 |
| Alanine | 3 | 965/973 | 0.026 (-0.041, 0.093) | 0.444 | 0.0 | 0.453 |
| Taurine | 2 | 909/913 | 0.029 (-0.069, 0.127) | 0.561 | 0.0 | 0.495 |
| Choline | 2 | 77/93 | 5.519 (-5.126, 16.164) | 0.310 | 98.9 | < 0.001 |
| Asparagine | 3 | 930/946 | -0.298 (-0.752, 0.156) | 0.198 | 83.2 | 0.003 |
| Isoleucine | 4 | 986/1006 | 0.020 (-0.097, 0.138) | 0.737 | 13.6 | 0.325 |
| Phenylalanine | 4 | 986/1006 | 0.015 (-0.050, 0.079) | 0.657 | 0.0 | 0.866 |
| Arginine | 4 | 985/987 | -0.160 (-0.399, 0.093) | 0.196 | 55.0 | 0.083 |
| Citrulline | 2 | 909/913 | -0.141 (-0.432, 0.151) | 0.344 | 64.0 | 0.095 |
| Histidine | 3 | 965/973 | -0.138 (-0.444, 0.169) | 0.379 | 74.2 | 0.021 |
| Leucine | 3 | 965/973 | 0.038 (-0.024, 0.101) | 0.232 | 0.0 | 0.756 |
| Lysine | 3 | 965/973 | 0.054 (-0.014, 0.121) | 0.120 | 0.0 | 0.959 |
| Methionine | 4 | 985/987 | -0.396 (-0.817, 0.025) | 0.065 | 85.4 | < 0.001 |
| Ornithine | 2 | 909/913 | 0.010 (-0.076, 0.096) | 0.827 | 0.0 | 0.738 |
| Proline | 4 | 1654/1662 | 0.062 (0.035, 0.088) | < 0.001 | 0.0 | 0.506 |
| Threonine | 3 | 965/973 | 0.008 (-0.051, 0.067) | 0.780 | 0.0 | 0.496 |
| Tryptophan | 3 | 929/927 | -0.342 (-0.838, 0.154) | 0.176 | 83.7 | 0.002 |
| Tyrosine | 3 | 965/973 | -0.042 (-0.206, 0.123) | 0.621 | 34.1 | 0.219 |
| Valine | 3 | 965/973 | 0.041 (-0.022, 0.104) | 0.203 | 0.0 | 0.725 |
| Creatinine | 3 | 965/973 | -0.027 (-0.087, 0.033) | 0.375 | 0.0 | 0.838 |
| Kynurenine | 2 | 909/913 | -0.311 (-0.926, 0.304) | 0.321 | 90.6 | 0.001 |
| Formate | 2 | 77/93 | 8.954 (-8.813, 26.721) | 0.323 | 99.0 | < 0.001 |
| Pyruvate | 2 | 77/93 | -0.738 (-2.516, 1.040) | 0.416 | 95.7 | < 0.001 |
| Asymmetric dimethylarginine | 2 | 909/913 | 0.135 (-0.255, 0.524) | 0.498 | 77.0 | 0.037 |
| Estrone-sulfate | 2 | 372/220 | 0.530 (0.169, 0.893) | 0.004 | 58.5 | 0.121 |
| Estrone | 2 | 372/220 | 0.882 (0.655, 1.108) | < 0.001 | 0.0 | 0.395 |
| Estradiol | 2 | 372/220 | 0.544 (0.114, 0.974) | 0.013 | 63.2 | 0.099 |
| SM (OH) C14:1 | 2 | 909/913 | 0.005 (-0.059, 0.070) | 0.872 | 0.0 | 0.447 |
| SM (OH) C16:1 | 2 | 909/913 | 0.052 (-0.012, 0.116) | 0.109 | 0.0 | 0.928 |
| SM (OH) C22:1 | 2 | 909/913 | -0.046 (-0.290, 0.198) | 0.711 | 53.3 | 0.143 |
| SM (OH) C22:2 | 2 | 909/913 | -0.104 (-0.402, 0.194) | 0.495 | 65.1 | 0.090 |
| SM (OH) C24:1 | 2 | 909/913 | 0.010 (-0.059, 0.080) | 0.767 | 0.0 | 0.481 |
| SM C16:0 | 2 | 909/913 | 0.011 (-0.048, 0.070) | 0.718 | 0.0 | 0.652 |
| SM C16:1 | 2 | 909/913 | -0.011 (-0.072, 0.050) | 0.719 | 0.0 | 0.590 |
| SM C18:0 | 2 | 909/913 | 0.172 (-0.077, 0.421) | 0.177 | 54.3 | 0.139 |
| SM C18:1 | 2 | 909/913 | 0.096 (-0.087, 0.279) | 0.306 | 35.3 | 0.214 |
| SM C20:2 | 2 | 909/913 | 0.034 (-0.044, 0.111) | 0.399 | 0.0 | 0.525 |
| SM C24:0 | 2 | 909/913 | -0.088 (-0.339, 0.163) | 0.493 | 55.0 | 0.136 |
| SM C24:1 | 2 | 909/913 | 0.044 (-0.021, 0.109) | 0.186 | 0.0 | 0.838 |
| SM C26:0 | 2 | 909/913 | 0.004 (-0.105, 0.113) | 0.943 | 11.8 | 0.287 |
| SM C26:1 | 2 | 909/913 | 0.019 (-0.086, 0.125) | 0.718 | 10.7 | 0.290 |
| C0 | 3 | 965/973 | -0.076 (-0.167, 0.014) | 0.098 | 7.7 | 0.339 |
| C10 | 3 | 1598/1602 | 0.092 (-0.027, 0.211) | 0.129 | 85.6 | 0.001 |
| C14:1 | 2 | 909/913 | 0.340 (-0.373, 1.053) | 0.350 | 92.8 | < 0.001 |
| C14:2 | 2 | 909/913 | 0.376 (-0.377, 1.129) | 0.328 | 93.5 | < 0.001 |
| C16 | 2 | 909/913 | 0.386 (-0.347, 1.119) | 0.302 | 93.1 | < 0.001 |
| C18 | 2 | 909/913 | -0.013 (-0.083, 0.057) | 0.718 | 0.0 | 0.943 |
| C18:1 | 2 | 909/913 | 0.490 (-0.459, 1.439) | 0.312 | 95.7 | < 0.001 |
| C18:2 | 2 | 909/913 | 0.488 (-0.465, 1.441) | 0.315 | 95.7 | < 0.001 |
| C2 | 2 | 909/913 | 0.331 (-0.335, 0.998) | 0.330 | 91.9 | < 0.001 |
| C3 | 2 | 909/913 | -0.096 (-0.380, 0.188) | 0.509 | 62.7 | 0.102 |
| C4 | 3 | 1598/1602 | -0.045 (-0.090, 0.001) | 0.055 | 74.5 | 0.020 |
| C5 | 2 | 909/913 | 0.019 (-0.113, 0.152) | 0.773 | 18.9 | 0.267 |
| C6 (C4:1-DC) | 2 | 909/913 | 0.378 (-0.371, 1.127) | 0.322 | 93.5 | < 0.001 |
| LysoPC a C16:0 | 2 | 909/913 | -0.132 (-0.452, 0.188) | 0.419 | 68.1 | 0.077 |
| LysoPC a C16:1 | 2 | 909/913 | -0.002 (-0.078, 0.074) | 0.965 | 0.0 | 0.637 |
| LysoPC a C17:0 | 2 | 909/913 | -0.263 (-0.830, 0.305) | 0.365 | 88.9 | 0.003 |
| LysoPC a C18:0 | 2 | 909/913 | -0.192 (-0.563, 0.179) | 0.310 | 75.5 | 0.043 |
| LysoPC a C18:1 | 2 | 909/913 | -0.231 (-0.686, 0.224) | 0.320 | 83.3 | 0.014 |
| LysoPC a C18:2 | 3 | 1598/1602 | -0.077 (-0.160, 0.006) | 0.069 | 67.5 | 0.046 |
| LysoPC a C20:3 | 2 | 909/913 | 0.012 (-0.050, 0.074) | 0.704 | 0.0 | 0.695 |
| LysoPC a C20:4 | 2 | 909/913 | 0.010 (-0.053, 0.073) | 0.754 | 0.0 | 0.418 |
| PC aa C28:1 | 3 | 1598/1602 | -0.035 (-0.010, 0.081) | 0.128 | 21.1 | 0.282 |
| PC aa C30:0 | 2 | 909/913 | -0.029 (-0.173, 0.116) | 0.697 | 22.5 | 0.256 |
| PC aa C32:0 | 2 | 909/913 | 0.019 (-0.054, 0.092) | 0.604 | 0.0 | 0.911 |
| PC aa C32:1 | 2 | 909/913 | 0.010 (-0.059, 0.079) | 0.779 | 0.0 | 0.926 |
| PC aa C32:2 | 3 | 1598/1602 | -0.047 (-0.085, -0.009) | 0.015 | 0.0 | 0.425 |
| PC aa C32:3 | 2 | 909/913 | -0.009 (-0.097, 0.080) | 0.849 | 0.0 | 0.817 |
| PC aa C34:1 | 2 | 909/913 | -0.009 (-0.115, 0.097) | 0.871 | 9.5 | 0.293 |
| PC aa C34:2 | 2 | 909/913 | -0.012 (-0.095, 0.071) | 0.784 | 0.0 | 0.343 |
| PC aa C34:3 | 2 | 909/913 | -0.061 (-0.216, 0.095) | 0.445 | 23.8 | 0.252 |
| PC aa C34:4 | 2 | 909/913 | -0.073 (-0.320, 0.173) | 0.561 | 53.7 | 0.142 |
| PC aa C36:0 | 3 | 1598/1602 | -0.101 (-0.225, 0.023) | 0.111 | 83.0 | 0.003 |
| PC aa C36:1 | 3 | 1598/1602 | -0.045 (-0.122, 0.032) | 0.252 | 65.3 | 0.056 |
| PC aa C36:2 | 2 | 909/913 | -0.146 (-0.439, 0.147) | 0.328 | 63.8 | 0.097 |
| PC aa C36:3 | 2 | 909/913 | -0.158 (-0.506, 0.190) | 0.375 | 72.8 | 0.055 |
| PC aa C36:4 | 2 | 909/913 | 0.015 (-0.053, 0.082) | 0.674 | 0.0 | 0.913 |
| PC aa C36:5 | 2 | 909/913 | -0.214 (-0.805, 0.378) | 0.478 | 89.9 | 0.002 |
| PC aa C36:6 | 2 | 909/913 | -0.285 (-0.967, 0.397) | 0.413 | 92.3 | < 0.001 |
| PC aa C38:0 | 2 | 909/913 | -0.194 (-0.630, 0.242) | 0.384 | 82.0 | 0.018 |
| PC aa C38:3 | 2 | 909/913 | 0.017 (-0.049, 0.082) | 0.622 | 0.0 | 0.769 |
| PC aa C38:4 | 2 | 909/913 | 0.010 (-0.054, 0.073) | 0.763 | 0.0 | 0.862 |
| PC aa C38:5 | 2 | 909/913 | -0.234 (-0.797, 0.330) | 0.416 | 88.9 | 0.003 |
| PC aa C38:6 | 2 | 909/913 | -0.115 (-0.493, 0.263) | 0.498 | 76.7 | 0.038 |
| PC aa C40:2 | 2 | 909/913 | -0.187 (-0.605, 0.232) | 0.382 | 80.7 | 0.023 |
| PC aa C40:3 | 2 | 909/913 | -0.145 (-0.545, 0.254) | 0.476 | 78.9 | 0.030 |
| PC aa C40:4 | 2 | 909/913 | -0.011 (-0.076, 0.053) | 0.729 | 0.0 | 0.378 |
| PC aa C40:5 | 2 | 909/913 | -0.003 (-0.068, 0.063) | 0.937 | 0.0 | 0.376 |
| PC aa C40:6 | 2 | 909/913 | 0.011 (-0.090, 0.111) | 0.837 | 8.8 | 0.295 |
| PC aa C42:0 | 2 | 909/913 | 0.049 (-0.014, 0.111) | 0.128 | 0.0 | 0.587 |
| PC aa C42:1 | 2 | 909/913 | 0.006 (-0.057, 0.069) | 0.842 | 0.0 | 0.972 |
| PC aa C42:2 | 2 | 909/913 | -0.271 (-0.813, 0.272) | 0.329 | 88.1 | 0.004 |
| PC aa C42:4 | 2 | 909/913 | -0.055 (-0.264, 0.153) | 0.604 | 43.7 | 0.183 |
| PC aa C42:5 | 3 | 970/978 | -0.240 (-0.634, 0.153) | 0.231 | 84.9 | 0.001 |
| PC aa C42:6 | 3 | 970/978 | -0.305 (-0.744, 0.134) | 0.173 | 87.8 | < 0.001 |
| PC ae C30:0 | 2 | 909/913 | -0.031 (-0.217, 0.154) | 0.740 | 35.9 | 0.212 |
| PC ae C30:2 | 2 | 909/913 | 0.046 (-0.019, 0.110) | 0.167 | 0.0 | 0.803 |
| PC ae C32:1 | 2 | 909/913 | 0.009 (-0.062, 0.080) | 0.809 | 0.0 | 0.447 |
| PC ae C32:2 | 2 | 909/913 | 0.003 (-0.105, 0.111) | 0.957 | 9.8 | 0.292 |
| PC ae C34:0 | 2 | 909/913 | -0.107 (-0.480, 0.266) | 0.573 | 76.1 | 0.041 |
| PC ae C34:1 | 2 | 909/913 | -0.108 (-0.402, 0.186) | 0.472 | 64.1 | 0.095 |
| PC ae C34:2 | 2 | 909/913 | -0.230 (-0.667, 0.207) | 0.303 | 81.9 | 0.019 |
| PC ae C34:3 | 2 | 909/913 | -0.228 (-0.642, 0.185) | 0.279 | 80.0 | 0.025 |
| PC ae C36:0 | 2 | 909/913 | -0.070 (-0.365, 0.224) | 0.640 | 64.4 | 0.094 |
| PC ae C36:1 | 2 | 909/913 | -0.218 (-0.735, 0.300) | 0.409 | 86.9 | 0.006 |
| PC ae C36:2 | 2 | 909/913 | -0.218 (-0.659, 0.224) | 0.334 | 82.3 | 0.018 |
| PC ae C36:3 | 2 | 909/913 | -0.261 (-0.746, 0.224) | 0.291 | 85.1 | 0.010 |
| PC ae C36:4 | 2 | 909/913 | -0.025 (-0.095, 0.044) | 0.475 | 0.8 | 0.315 |
| PC ae C36:5 | 2 | 909/913 | -0.114 (-0.392, 0.165) | 0.423 | 61.3 | 0.108 |
| PC ae C38:0 | 2 | 909/913 | -0.315 (-1.041, 0.411) | 0.396 | 93.1 | < 0.001 |
| PC ae C38:2 | 2 | 909/913 | -0.212 (-0.654, 0.230) | 0.347 | 82.5 | 0.017 |
| PC ae C38:3 | 2 | 909/913 | 0.020 (-0.047, 0.087) | 0.557 | 0.0 | 0.369 |
| PC ae C38:4 | 2 | 909/913 | 0.006 (-0.060, 0.072) | 0.860 | 0.0 | 0.525 |
| PC ae C38:5 | 2 | 909/913 | -0.134 (-0.454, 0.187) | 0.414 | 69.1 | 0.072 |
| PC ae C38:6 | 2 | 909/913 | -0.239 (-0.777, 0.300) | 0.385 | 87.9 | 0.004 |
| PC ae C40:1 | 2 | 909/913 | -0.420 (-1.273, 0.434) | 0.335 | 94.8 | < 0.001 |
| PC ae C40:2 | 2 | 909/913 | 0.052 (-0.011, 0.114) | 0.104 | 0.0 | 0.365 |
| PC ae C40:3 | 2 | 909/913 | 0.024 (-0.040, 0.088) | 0.460 | 0.0 | 0.895 |
| PC ae C40:4 | 2 | 909/913 | 0.023 (-0.039, 0.085) | 0.470 | 0.0 | 0.632 |
| PC ae C40:5 | 2 | 909/913 | -0.028 (-0.212, 0.157) | 0.769 | 35.6 | 0.213 |
| PC ae C40:6 | 2 | 909/913 | -0.198 (-0.683, 0.288) | 0.425 | 85.3 | 0.009 |
| PC ae C42:1 | 2 | 909/913 | -0.239 (-0.804, 0.326) | 0.407 | 89.0 | 0.003 |
| PC ae C42:2 | 2 | 909/913 | -0.254 (-0.838, 0.330) | 0.393 | 89.7 | 0.002 |
| PC ae C42:3 | 2 | 909/913 | -0.118 (-0.512, 0.275) | 0.555 | 78.4 | 0.031 |
| PC ae C42:4 | 2 | 909/913 | 0.025 (-0.034, 0.083) | 0.412 | 0.0 | 0.947 |
| PC ae C42:5 | 2 | 909/913 | 0.041 (-0.016, 0.099) | 0.161 | 0.0 | 0.894 |
| PC ae C44:3 | 2 | 909/913 | 0.031 (-0.032, 0.093) | 0.333 | 0.0 | 0.867 |
| PC ae C44:4 | 2 | 909/913 | 0.039 (-0.021, 0.099) | 0.205 | 0.0 | 0.763 |
| PC ae C44:5 | 2 | 909/913 | 0.060 (-0.001, 0.120) | 0.052 | 0.0 | 0.387 |
| PC ae C44:6 | 2 | 909/913 | 0.043 (-0.016, 0.103) | 0.154 | 0.0 | 0.511 |

Abbreviations: EC, endometrial cancer; C0, carnitine; C10, decanoylcarnitine; decanoylcarnitine; C14:1, tetradecenoylcarnitine; C14:2, tetradecadienoylcarnitine; C16, hexadecenoylcarnitine; C18, 3-hydroxylhexadecanoylcarnitine; C18:1, octadecenoylcarnitine; C18:2, octadecadienoylcarnitine; C2, acetylcarnitine; C3, propionylcarnitine; C4, malonylcarnitine; C5, methylmalonylcarnitine; C6, glutarylcarnitine; LysoPC, lysophosphatidylcholine; PC, phosphatidylcholine; SM, sphingomyelin; SMD, standardized mean difference

**Table S4: 181 metabolites and the corresponding main metabolic pathways.**

| **Metabolite** | **HMDB ID** | **Pathway** | **Reference** |
| --- | --- | --- | --- |
| Dehydroepiandrosterone-sulfate | HMDB0001032 | Cholesterol hormone metabolism | [ 2] |
| Dehydroepiandrosterone | HMDB0000077 | Cholesterol hormone metabolism | [2] |
| Androstenediol | HMDB0003818 | Cholesterol hormone metabolism | [2] |
| Androstenedione | HMDB0000053 | Cholesterol hormone metabolism | [2] |
| Testosterone | HMDB0000234 | Cholesterol hormone metabolism | [2] |
| Dihydrotestosterone | HMDB0002961 | Cholesterol hormone metabolism | [2] |
| Androsterone-glucuronide | HMDB0002829 | Cholesterol hormone metabolism | [2] |
| Androstane-3α, 17β-diol 3-glucuronide | NA | Cholesterol hormone metabolism | [2] |
| Androstane-3α, 17β-diol 17-glucuronide | NA | Cholesterol hormone metabolism | [2] |
| Estrone-sulfate | HMDB0001425 | Cholesterol hormone metabolism | [2, 21] |
| Estrone | HMDB0000145 | Cholesterol hormone metabolism | [2, 21] |
| Estradiol | HMDB0000151 | Cholesterol hormone metabolism | [2, 21] |
| Choline | HMDB0000097 | Choline metabolism | [11, 12] |
| Formate | HMDB0000142 | Carbohydrate metabolism | [11, 12] |
| Fumarate | HMDB0000134 | Carbohydrate metabolism | [11] |
| Malate | HMDB0000156 | Carbohydrate metabolism | [11] |
| Phosphorylcholine | HMDB0001565 | Glycerophospholipid metabolism | [11] |
| Asparagine | HMDB0000168 | Alanine, aspartate, and glutamate metabolism | [11, 12, 17] |
| Aspartate | HMDB0000191 | Alanine, aspartate, and glutamate metabolism | [11, 12] |
| Isoleucine | HMDB0000172 | BCAA metabolism | [11, 12, 17] |
| Phenylalanine | HMDB0000159 | AAA metabolism | [11, 12, 17] |
| Pyruvate | HMDB0000243 | Carbohydrate metabolism | [11, 12] |
| C0 | HMDB0000062 | Acylcarnitine metabolism | [12, 17] |
| C10 | HMDB0000651 | Acylcarnitine metabolism | [12, 17, 28] |
| C14:1 | CHEBI:86066 | Acylcarnitine metabolism | [12, 17] |
| C14:2 | CHEBI:86069 | Acylcarnitine metabolism | [12, 17] |
| C16 | HMDB0000222 | Acylcarnitine metabolism | [12, 17] |
| C18 | HMDB0000848 | Acylcarnitine metabolism | [12, 17] |
| C18:1 | CHEBI:86475 | Acylcarnitine metabolism | [12, 17] |
| C18:2 | CHEBI:86480 | Acylcarnitine metabolism | [12, 17] |
| C2 | HMDB0000201 | Acylcarnitine metabolism | [12, 17] |
| C3 | HMDB0000824 | Acylcarnitine metabolism | [12, 17] |
| C3-DC | HMDB0002095 | Acylcarnitine metabolism | [16] |
| C4 | HMDB0002013 | Acylcarnitine metabolism | [12, 17, 28] |
| C6 (C4:1-DC) | HMDB0000756 | Acylcarnitine metabolism | [12, 17] |
| C5 | HMDB0013128 | Acylcarnitine metabolism | [12, 17] |
| C5-DC (C6-OH) | HMDB0013130 | Acylcarnitine metabolism | [12] |
| C7-DC | NA | Acylcarnitine metabolism | [12] |
| C8 | HMDB0000791 | Acylcarnitine metabolism | [12] |
| LysoPC a C16:0 | HMDB0010382 | Glycerophospholipid metabolism | [12, 17] |
| LysoPC a C16:1 | HMDB0010383 | Glycerophospholipid metabolism | [12, 17] |
| LysoPC a C17:0 | HMDB0012108 | Glycerophospholipid metabolism | [12, 17] |
| LysoPC a C18:0 | HMDB0010384 | Glycerophospholipid metabolism | [12, 17] |
| LysoPC a C18:1 | HMDB0010385 | Glycerophospholipid metabolism | [12, 17] |
| LysoPC a C18:2 | HMDB0010386 | Glycerophospholipid metabolism | [12, 17, 28] |
| LysoPC a C20:3 | HMDB0010394 | Glycerophospholipid metabolism | [12, 17] |
| LysoPC a C20:4 | HMDB0010396 | Glycerophospholipid metabolism | [12, 17] |
| LysoPC a C26:0 | HMDB0029205 | Glycerophospholipid metabolism | [12] |
| LysoPC a C26:1 | HMDB0029220 | Glycerophospholipid metabolism | [12] |
| LysoPC a C28:0 | HMDB0029206 | Glycerophospholipid metabolism | [12] |
| LysoPC a C28:1 | HMDB0029221 | Glycerophospholipid metabolism | [12] |
| PC aa C24:0 | HMDB0010405 | Glycerophospholipid metabolism | [12] |
| PC aa C28:1 | HMDB0007899 | Glycerophospholipid metabolism | [12, 17, 28] |
| PC aa C30:0 | HMDB0007895 | Glycerophospholipid metabolism | [12, 17] |
| PC aa C30:2 | HMDB0007903 | Glycerophospholipid metabolism | [12] |
| PC aa C32:0 | HMDB0007994 | Glycerophospholipid metabolism | [12, 17] |
| PC aa C32:1 | HMDB0007872 | Glycerophospholipid metabolism | [12, 17] |
| PC aa C32:2 | HMDB0007874 | Glycerophospholipid metabolism | [12, 17, 28] |
| PC aa C32:3 | HMDB0007875 | Glycerophospholipid metabolism | [12, 17] |
| PC aa C34:1 | HMDB0007996 | Glycerophospholipid metabolism | [12, 17] |
| PC aa C34:2 | HMDB0008159 | Glycerophospholipid metabolism | [12, 17] |
| PC aa C34:3 | HMDB0007881 | Glycerophospholipid metabolism | [12, 17] |
| PC aa C34:4 | HMDB0007883 | Glycerophospholipid metabolism | [12, 17] |
| PC aa C36:0 | HMDB0008061 | Glycerophospholipid metabolism | [12, 17, 28] |
| PC aa C36:1 | HMDB0007887 | Glycerophospholipid metabolism | [12, 17, 28] |
| PC aa C36:2 | HMDB0008039 | Glycerophospholipid metabolism | [12, 17] |
| PC aa C36:3 | HMDB0007980 | Glycerophospholipid metabolism | [12, 17] |
| PC aa C36:4 | HMDB0007982 | Glycerophospholipid metabolism | [12, 17] |
| PC aa C36:5 | HMDB0007890 | Glycerophospholipid metabolism | [12, 17] |
| PC aa C36:6 | HMDB0008722 | Glycerophospholipid metabolism | [12, 17] |
| PC aa C38:0 | HMDB0008554 | Glycerophospholipid metabolism | [12, 17] |
| PC aa C38:1 | HMDB0008268 | Glycerophospholipid metabolism | [12] |
| PC aa C38:3 | HMDB0008046 | Glycerophospholipid metabolism | [12, 17] |
| PC aa C38:4 | HMDB0008489 | Glycerophospholipid metabolism | [12, 17] |
| PC aa C38:5 | HMDB0007989 | Glycerophospholipid metabolism | [12, 17] |
| PC aa C38:6 | HMDB0007991 | Glycerophospholipid metabolism | [12, 17] |
| PC aa C40:2 | HMDB0008595 | Glycerophospholipid metabolism | [12, 17] |
| PC aa C40:3 | HMDB0008278 | Glycerophospholipid metabolism | [12, 17] |
| PC aa C40:4 | HMDB0008054 | Glycerophospholipid metabolism | [12, 17] |
| PC aa C40:5 | HMDB0008055 | Glycerophospholipid metabolism | [12, 17] |
| PC aa C40:6 | HMDB0008057 | Glycerophospholipid metabolism | [12, 17] |
| PC aa C42:0 | HMDB0008785 | Glycerophospholipid metabolism | [12, 17] |
| PC aa C42:1 | HMDB0008059 | Glycerophospholipid metabolism | [12, 17] |
| PC aa C42:2 | HMDB0008539 | Glycerophospholipid metabolism | [12, 17] |
| PC aa C42:4 | HMDB0008635 | Glycerophospholipid metabolism | [12, 17] |
| PC aa C42:5 | HMDB0008668 | Glycerophospholipid metabolism | [12, 15, 17] |
| PC aa C42:6 | HMDB0008288 | Glycerophospholipid metabolism | [12, 15, 17] |
| PC ae C30:0 | HMDB0013341 | Glycerophospholipid metabolism | [12, 17] |
| PC ae C30:1 | HMDB0013402 | Glycerophospholipid metabolism | [12] |
| PC ae C30:2 | HMDB0013410 | Glycerophospholipid metabolism | [12, 17] |
| PC ae C32:1 | HMDB0013404 | Glycerophospholipid metabolism | [12, 17] |
| PC ae C32:2 | HMDB0013411 | Glycerophospholipid metabolism | [12, 17] |
| PC ae C34:0 | HMDB0013405 | Glycerophospholipid metabolism | [12, 17] |
| PC ae C34:1 | HMDB0013412 | Glycerophospholipid metabolism | [12, 17] |
| PC ae C34:2 | HMDB0011151 | Glycerophospholipid metabolism | [12, 17] |
| PC ae C34:3 | HMDB0013413 | Glycerophospholipid metabolism | [12, 17] |
| PC ae C36:0 | HMDB0013417 | Glycerophospholipid metabolism | [12, 17] |
| PC ae C36:1 | HMDB0013414 | Glycerophospholipid metabolism | [12, 17] |
| PC ae C36:2 | HMDB0013418 | Glycerophospholipid metabolism | [12, 17] |
| PC ae C36:3 | HMDB0013429 | Glycerophospholipid metabolism | [12, 17] |
| PC ae C36:4 | HMDB0013407 | Glycerophospholipid metabolism | [12, 17] |
| PC ae C36:5 | HMDB0013415 | Glycerophospholipid metabolism | [12, 17] |
| PC ae C38:0 | HMDB0013408 | Glycerophospholipid metabolism | [12, 17] |
| PC ae C38:1 | HMDB0013430 | Glycerophospholipid metabolism | [12] |
| PC ae C38:2 | HMDB0013436 | Glycerophospholipid metabolism | [12, 17] |
| PC ae C38:3 | HMDB0013439 | Glycerophospholipid metabolism | [12, 17] |
| PC ae C38:4 | HMDB0013420 | Glycerophospholipid metabolism | [12, 17] |
| PC ae C38:5 | HMDB0013432 | Glycerophospholipid metabolism | [12, 17] |
| PC ae C38:6 | HMDB0013409 | Glycerophospholipid metabolism | [12, 17] |
| PC ae C40:1 | HMDB0013433 | Glycerophospholipid metabolism | [12, 17] |
| PC ae C40:2 | HMDB0013437 | Glycerophospholipid metabolism | [12, 17] |
| PC ae C40:3 | HMDB0013445 | Glycerophospholipid metabolism | [12, 17] |
| PC ae C40:4 | HMDB0013442 | Glycerophospholipid metabolism | [12, 17] |
| PC ae C40:5 | HMDB0013444 | Glycerophospholipid metabolism | [12, 17] |
| PC ae C40:6 | HMDB0013422 | Glycerophospholipid metabolism | [12, 17] |
| PC ae C42:1 | HMDB0013434 | Glycerophospholipid metabolism | [12, 17] |
| PC ae C42:2 | HMDB0013438 | Glycerophospholipid metabolism | [12, 17] |
| PC ae C42:3 | HMDB0013458 | Glycerophospholipid metabolism | [12, 17] |
| PC ae C42:4 | HMDB0013448 | Glycerophospholipid metabolism | [12, 17] |
| PC ae C42:5 | HMDB0013451 | Glycerophospholipid metabolism | [12, 17] |
| PC ae C44:3 | HMDB0013452 | Glycerophospholipid metabolism | [12, 17] |
| PC ae C44:4 | HMDB0013460 | Glycerophospholipid metabolism | [12, 17] |
| PC ae C44:5 | HMDB0013456 | Glycerophospholipid metabolism | [12, 17] |
| PC ae C44:6 | HMDB0013450 | Glycerophospholipid metabolism | [12, 17] |
| SM (OH) C14:1 | HMDB0013462 | Sphingolipid metabolism | [12, 17] |
| SM (OH) C16:1 | HMDB0013463 | Sphingolipid metabolism | [12, 17] |
| SM (OH) C22:1 | HMDB0013466 | Sphingolipid metabolism | [12, 17] |
| SM (OH) C22:2 | HMDB0013467 | Sphingolipid metabolism | [12, 17] |
| SM (OH) C24:1 | HMDB0013469 | Sphingolipid metabolism | [12, 17] |
| SM C16:0 | NA | Sphingolipid metabolism | [12, 17] |
| SM C16:1 | HMDB0029216 | Sphingolipid metabolism | [12, 17] |
| SM C18:0 | NA | Sphingolipid metabolism | [12, 17] |
| SM C18:1 | HMDB0012101 | Sphingolipid metabolism | [12, 17] |
| SM C20:2 | HMDB0013465 | Sphingolipid metabolism | [12, 17] |
| SM C22:3 | HMDB0013468 | Sphingolipid metabolism | [12] |
| SM C24:0 | NA | Sphingolipid metabolism | [12, 17] |
| SM C24:1 | HMDB0012107 | Sphingolipid metabolism | [12, 17] |
| SM C26:0 | NA | Sphingolipid metabolism | [12, 17] |
| SM C26:1 | HMDB0013461 | Sphingolipid metabolism | [12, 17] |
| Hexose | HMDB0250761 | Carbohydrate metabolism | [12, 17] |
| Alanine | HMDB0000161 | Alanine, aspartate, and glutamate metabolism | [12, 17] |
| Arginine | HMDB0000517 | Arginine and proline metabolism, Urea cycle | [12, 16, 17] |
| Citrulline | HMDB0000904 | Arginine and proline metabolism, Urea cycle | [12, 17] |
| Glutamine | HMDB0000641 | Alanine, aspartate, and glutamate metabolism | [12, 17, 28] |
| Glutamate | HMDB0000148 | Alanine, aspartate, and glutamate metabolism | [12, 17] |
| Glycine | HMDB0000123 | Glycine, serine, and threonine metabolism | [12, 17] |
| Histidine | HMDB0000177 | Histidine metabolism | [12, 17] |
| Leucine | HMDB0000687 | BCAA metabolism | [12, 17] |
| Lysine | HMDB0000182 | Lysine metabolism | [12, 17] |
| Methionine | HMDB0000696 | Methionine metabolism | [12, 16, 17] |
| Ornithine | HMDB0000214 | Arginine and proline metabolism, Urea cycle | [12, 17] |
| Proline | HMDB0000162 | Arginine and proline metabolism, Urea cycle | [12, 17, 28] |
| Serine | HMDB0000187 | Glycine, serine, and threonine metabolism | [12, 17] |
| Threonine | HMDB0000167 | Glycine, serine, and threonine metabolism | [12, 17] |
| Tryptophan | HMDB0000929 | Tryptophan Metabolism | [12, 16, 17] |
| Tyrosine | HMDB0000158 | AAA metabolism | [12, 17] |
| Valine | HMDB0000883 | BCAA metabolism | [12, 17] |
| Acetylornithine | HMDB0003357 | Arginine and proline metabolism, Urea cycle | [12] |
| Asymmetric dimethylarginine | HMDB0001539 | Arginine and proline metabolism, Urea cycle | [12, 17] |
| Symmetric dimethylarginine | HMDB0003334 | Arginine and proline metabolism, Urea cycle | [12] |
| Aminoadipic acid | HMDB0000510 | Lysine metabolism | [12] |
| Creatinine | HMDB0000562 | Arginine and proline metabolism, Urea cycle | [12, 17] |
| Kynurenine | HMDB0000684 | Tryptophan Metabolism | [12, 17] |
| Hydroxyproline | HMDB0240251 | Arginine and proline metabolism, Urea cycle | [12] |
| Putrescine | HMDB0001414 | Methionine metabolism | [12] |
| Serotonin | HMDB0000259 | Tryptophan Metabolism | [12] |
| Taurine | HMDB0000251 | Taurine and hypotaurine metabolism | [12, 17] |
| 1-Methylhistidine | HMDB0000001 | Histidine metabolism | [12] |
| 2-Hydroxybutyrate | HMDB0000008 | Glycine, serine, and threonine metabolism | [12] |
| Acetic acid | HMDB0000042 | Carbohydrate metabolism | [12] |
| Betaine | HMDB0000043 | Choline metabolism | [12] |
| Creatine | HMDB0000064 | Glycine, serine, and threonine metabolism | [12] |
| Citric acid | HMDB0000094 | Carbohydrate metabolism | [12] |
| Glucose | HMDB0000122 | Carbohydrate metabolism | [12] |
| Glycerol | HMDB0000131 | Carbohydrate metabolism | [12] |
| Lactic acid | HMDB0000190 | Carbohydrate metabolism | [12] |
| 3-Hydroxybutyric acid | HMDB0000011 | Carbohydrate metabolism | [12] |
| Acetone | HMDB0001659 | Carbohydrate metabolism | [12] |
| Methanol | HMDB0001875 | Carbohydrate metabolism | [12] |
| 4-Hydroxyproline | HMDB0000725 | Arginine and proline metabolism, Urea cycle | [17] |
| PC aa C40:1 | HMDB0008275 | Glycerophospholipid metabolism | [15] |

Note: NA, not available.

Abbreviations: AAA, aromatic amino acid; BCAA, branched-chain amino acid; C0, carnitine; C10, decanoylcarnitine; C14:1, tetradecenoylcarnitine; C14:2, tetradecadienoylcarnitine; C16, hexadecenoylcarnitine; C18, 3-hydroxylhexadecanoylcarnitine; C18:1, octadecenoylcarnitine; C18:2, octadecadienoylcarnitine; C2, acetylcarnitine; C3, propionylcarnitine; C3-DC, malonylcarnitine; C4, malonylcarnitine; C5, methylmalonylcarnitine; C5-DC, 3-hydroxyl-isovalerylcarnitine; C6, glutarylcarnitine; C7-DC, pimelylcarnitine; C8, 3-methylglutarylcarnitine; LysoPC, lysophosphatidylcholine; PC, Phosphatidylcholine; SM, sphingomyelin.

**Table S5: Effect size of metabolic pathways between endometrial cancer patients and controls.**

| **Pathway** | **N Comparisons** | **N EC/Control** | **SMD (95% CI)** | ***p*-value (overall)** | **I^2^ (%)** | ***p*-value (heterogeneity)** | ***p*-value (Egger)** |
| --- | --- | --- | --- | --- | --- | --- | --- |
| Cholesterol hormone metabolism | 15 | 3330/1650 | 0.750 (0.598, 0.903) | < 0.001 | 61.9 | 0.001 | 0.147 |
| Choline metabolism | 3 | 133/153 | 2.929 (0.858, 5.001) | 0.006 | 97.8 | < 0.001 | —— |
| Carbohydrate metabolism | 16 | 1553/1645 | 0.762 (0.333, 1.191) | < 0.001 | 95.6 | < 0.001 | 0.015 |
| Alanine, aspartate, and glutamate metabolism | 15 | 4591/4647 | -0.007 (-0.064, 0.050) | 0.814 | 58.4 | 0.002 | 0.750 |
| BCAA metabolism | 10 | 2916/2952 | 0.041 (0.005, 0.078) | 0.026 | 0.0 | 0.860 | 0.003 |
| AAA metabolism | 7 | 1951/1979 | 0.016 (-0.028, 0.060) | 0.481 | 0.0 | 0.708 | —— |
| Arginine and proline metabolism, Urea cycle | 21 | 7352/7394 | -0.009 (-0.051, 0.032) | 0.663 | 53.5 | 0.002 | 0.024 |
| Glycine, serine, and threonine metabolism | 10 | 2951/2979 | -0.057 (-0.128, 0.013) | 0.110 | 50.8 | 0.032 | 0.440 |
| Histidine metabolism | 4 | 1021/1033 | -0.097 (-0.318, 0.124) | 0.389 | 61.4 | 0.051 | —— |
| Lysine metabolism | 4 | 1021/1033 | 0.056 (-0.011, 0.122) | 0.100 | 0.0 | 0.976 | —— |
| Methionine metabolism | 5 | 1041/1047 | -0.227 (-0.582, 0.127) | 0.209 | 84.5 | < 0.001 | —— |
| Tryptophan Metabolism | 6 | 1894/1900 | -0.164 (-0.315, -0.014) | 0.033 | 80.0 | < 0.001 | —— |
| Taurine and hypotaurine metabolism | 2 | 909/913 | 0.029 (-0.069, 0.127) | 0.561 | 0.0 | 0.495 | —— |
| Acylcarnitine metabolism | 33 | 13439/13501 | 0.092 (0.046, 0.137) | < 0.001 | 84.7 | < 0.001 | < 0.001 |
| Glycerophospholipid metabolism | 170 | 73237/73601 | -0.030 (-0.045, -0.015) | < 0.001 | 68.0 | < 0.001 | < 0.001 |
| Sphingolipid metabolism | 29 | 12782/12842 | 0.025 (0.008, 0.042) | 0.004 | 0.0 | 0.472 | 0.286 |

Abbreviations: AAA, aromatic amino acid; BCAA, branched-chain amino acid; CI, confidence intervals; EC, endometrial cancer; SMD, standardized mean difference.

**Table S6: Publication bias and correction of metabolic pathway.**

| **Pathway** | ***p*-value (Egger)** | **SMD (95% CI)** | **Trim and Fill**  **SMD (95% CI) + AS** |
| --- | --- | --- | --- |
| Cholesterol hormone metabolism | 0.147 | 0.750 (0.598, 0.903) | —— |
| Choline metabolism | —— | 2.929 (0.858, 5.001) | —— |
| Carbohydrate metabolism | 0.015 | 0.762 (0.333, 1.191) | No missing studies |
| Alanine, aspartate, and glutamate metabolism | 0.750 | -0.007 (-0.064, 0.050) | —— |
| BCAA metabolism | 0.003 | 0.041 (0.005, 0.078) | No missing studies |
| AAA metabolism | —— | 0.016 (-0.028, 0.060) | —— |
| Arginine and proline metabolism, Urea cycle | 0.024 | -0.009 (-0.051, 0.032) | No missing studies |
| Glycine, serine, and threonine metabolism | 0.440 | -0.057 (-0.128, 0.013) | —— |
| Histidine metabolism | —— | -0.097 (-0.318, 0.124) | —— |
| Lysine metabolism | —— | 0.056 (-0.011, 0.122) | —— |
| Methionine metabolism | —— | -0.227 (-0.582, 0.127) | —— |
| Tryptophan Metabolism | —— | -0.164 (-0.315, -0.014) | —— |
| Taurine and hypotaurine metabolism | —— | 0.029 (-0.069, 0.127) | —— |
| Acylcarnitine metabolism | < 0.001 | 0.092 (0.046, 0.137) | 0.077 (0.029, 0.126) +2 |
| Glycerophospholipid metabolism | < 0.001 | -0.030 (-0.045, -0.015) | No missing studies |
| Sphingolipid metabolism | 0.286 | 0.025 (0.008, 0.042) | —— |

Abbreviations: AAA, aromatic amino acid; AS, added studies; BCAA, branched-chain amino acid; CI, confidence intervals; SMD, standardized mean difference.

**Table S7: Sensitivity analysis, according to data conversion.**

| **Pathways** | **No. of comparisons** | **SMD (95% CI)** | ***p*-value (overall)** | **I^2^ (%)** | ***p*-value (heterogeneity)** |
| --- | --- | --- | --- | --- | --- |
| Cholesterol hormone metabolism | 15 | 0.750 (0.598, 0.903) | < 0.001 | 61.9 | 0.001 |
| Cholesterol hormone metabolism^†^ | 3 | 0.664 (0.334, 0.994) | < 0.001 | 78.7 | 0.009 |
| Carbohydrate metabolism | 16 | 0.762 (0.333, 1.191) | < 0.001 | 95.6 | < 0.001 |
| Carbohydrate metabolism^†^ | 15 | 1.119 (0.549, 1.689) | < 0.001 | 95.8 | < 0.001 |
| Alanine, aspartate, and glutamate metabolism | 15 | -0.007 (-0.064, 0.050) | 0.814 | 58.4 | 0.002 |
| Alanine, aspartate, and glutamate metabolism^†^ | 10 | -0.067 (-0.265, 0.131) | 0.507 | 60.6 | 0.007 |
| BCAA metabolism | 10 | 0.041 (0.005, 0.078) | 0.026 | 0.0 | 0.860 |
| BCAA metabolism^†^ | 7 | -0.074 (-0.218, 0.070) | 0.312 | 0.0 | 0.923 |
| Arginine and proline metabolism, Urea cycle | 21 | -0.009 (-0.051, 0.032) | 0.663 | 53.5 | 0.002 |
| Arginine and proline metabolism, Urea cycle^†^ | 13 | -0.081 (-0.217, 0.055) | 0.242 | 40.2 | 0.066 |
| Glycine, serine, and threonine metabolism | 10 | -0.057 (-0.128, 0.013) | 0.110 | 50.8 | 0.032 |
| Glycine, serine, and threonine metabolism^†^ | 7 | -0.122 (-0.321, 0.077) | 0.229 | 51.8 | 0.053 |
| Acylcarnitine metabolism | 33 | 0.092 (0.046, 0.137) | < 0.001 | 84.7 | < 0.001 |
| Acylcarnitine metabolism^†^ | 18 | 0.437 (0.201, 0.672) | < 0.001 | 85.3 | < 0.001 |
| Glycerophospholipid metabolism | 170 | -0.030 (-0.045, -0.015) | < 0.001 | 68.0 | < 0.001 |
| Glycerophospholipid metabolism^†^ | 89 | -0.251 (-0.318, -0.185) | < 0.001 | 65.3 | < 0.001 |
| Sphingolipid metabolism | 29 | 0.025 (0.008, 0.042) | 0.004 | 0.0 | 0.472 |
| Sphingolipid metabolism^†^ | 15 | -0.027 (-0.133, 0.079) | 0.616 | 20.5 | 0.226 |

^†^: metabolites analyzed in the sensitivity analysis that removing the converting values.

Abbreviations: BCAA, branched-chain amino acid; CI, confidence intervals; SMD, standardized mean difference.

**Table S8: Meta-regression analysis of sample size.**

| **Pathways** | **No. of Comparisons** | **Regression coefficient** | **95% CI** | ***p*-value** |
| --- | --- | --- | --- | --- |
| Cholesterol hormone metabolism | 15 | 0.00097 | (-0.00238, -0.00433) | 0.541 |
| Carbohydrate metabolism | 16 | -0.00247 | (-0.01088, 0.00595) | 0.540 |
| Alanine, aspartate, and glutamate metabolism | 15 | 0.00005 | (-0.00007, 0.00018) | 0.381 |
| BCAA metabolism | 10 | 0.00008 | (-0.00003, 0.00019) | 0.139 |
| Arginine and proline metabolism, Urea cycle | 21 | 0.00003 | (-0.00005, 0.00012) | 0.425 |
| Glycine, serine, and threonine metabolism | 10 | 0.00005 | (-0.00009, 0.00020) | 0.427 |
| Acylcarnitine metabolism | 33 | -0.00027 | (-0.00042, -0.00011) | 0.001 |
| Glycerophospholipid metabolism | 170 | 0.00017 | (0.00013, 0.00020) | < 0.001 |
| Sphingolipid metabolism | 29 | 0.00003 | (-0.00003, 0.00010) | 0.281 |

Abbreviations: BCAA, branched-chain amino acid; CI, confidence intervals.

**Table S9: GRADE assessment for all outcomes of endometrial cancer biomarkers evaluated in this meta-analysis.**

| **Certainty assessment** | | | | | | | **Relative Effect (95% CI)** | **Certainty** | **Importance** |
| --- | --- | --- | --- | --- | --- | --- | --- | --- | --- |
| **№ of studies** | **Study design** | **Risk of bias** | **Inconsistency** | **Indirectness** | **Imprecision** | **Publication bias** |  |  |  |
| **Hexose** | | | | | | | | | |
| 2 | observational studies | Not serious | Serious^a^ | Not serious | Not serious | Undetected^g^ | **SMD 0.095**  (-0.247 to 0.436) | ⨁◯◯◯  Very low | CRITICAL |
| **Glutamine** | | | | | | | | | |
| 4 | observational studies | Not serious | Not serious | Not serious | Not serious | Undetected^g^ | **SMD -0.040**  (-0.069 to -0.011) | ⨁⨁◯◯  Low | CRITICAL |
| **Glutamate** | | | | | | | | | |
| 2 | observational studies | Not serious | Serious^b^ | Not serious | Not serious | Undetected^g^ | **SMD 0.271**  (-0.201 to 0.742) | ⨁◯◯◯  Very low | CRITICAL |
| **Glycine** | | | | | | | | | |
| 3 | observational studies | Not serious | Not serious | Not serious | Not serious | Undetected^g^ | **SMD -0.084**  (-0.168 to -0.000) | ⨁⨁◯◯  Low | CRITICAL |
| **Serine** | | | | | | | | | |
| 2 | observational studies | Not serious | Not serious | Not serious | Not serious | Undetected^g^ | **SMD -0.134**  (-0.364 to 0.097) | ⨁⨁◯◯  Low | CRITICAL |
| **Aspartate** | | | | | | | | | |
| 2 | observational studies | Not serious | Not serious | Not serious | Serious^e^ | Undetected^g^ | **SMD -0.074**  (-0.392 to 0.245) | ⨁◯◯◯  Very low | CRITICAL |
| **Alanine** | | | | | | | | | |
| 3 | observational studies | Not serious | Not serious | Not serious | Not serious | Undetected^g^ | **SMD 0.026**  (-0.041 to 0.093) | ⨁⨁◯◯  Low | CRITICAL |
| **Taurine** | | | | | | | | | |
| 2 | observational studies | Not serious | Not serious | Not serious | Not serious | Undetected^g^ | **SMD 0.029**  (-0.069 to 0.127) | ⨁⨁◯◯  Low | CRITICAL |
| **Choline** | | | | | | | | | |
| 2 | observational studies | Not serious | Serious^b^ | Not serious | Serious^f^ | Undetected^g^ | **SMD 5.519**  (-5.126 to 16.164) | ⨁◯◯◯  Very low | CRITICAL |
| **Asparagine** | | | | | | | | | |
| 3 | observational studies | Not serious | Serious^a^ | Not serious | Not serious | Undetected^g^ | **SMD -0.298**  (-0.752 to 0.156) | ⨁◯◯◯  Very low | CRITICAL |
| **Isoleucine** | | | | | | | | | |
| 4 | observational studies | Not serious | Not serious | Not serious | Not serious | Undetected^g^ | **SMD 0.020**  (-0.097 to 0.138) | ⨁⨁◯◯  Low | CRITICAL |
| **Phenylalanine** | | | | | | | | | |
| 4 | observational studies | Not serious | Not serious | Not serious | Not serious | Undetected^g^ | **SMD 0.015**  (-0.050 to 0.079) | ⨁⨁◯◯  Low | CRITICAL |
| **Arginine** | | | | | | | | | |
| 4 | observational studies | Not serious | Serious^d^ | Not serious | Not serious | Undetected^g^ | **SMD -0.160**  (-0.399 to 0.093) | ⨁◯◯◯  Very low | CRITICAL |
| **Citrulline** | | | | | | | | | |
| 2 | observational studies | Not serious | Serious^d^ | Not serious | Not serious | Undetected^g^ | **SMD -0.141**  (-0.432 to 0.151) | ⨁◯◯◯  Very low | CRITICAL |
| **Histidine** | | | | | | | | | |
| 3 | observational studies | Not serious | Serious^a^ | Not serious | Not serious | Undetected^g^ | **SMD -0.138**  (-0.444 to 0.169) | ⨁◯◯◯  Very low | CRITICAL |
| **Leucine** | | | | | | | | | |
| 3 | observational studies | Not serious | Not serious | Not serious | Not serious | Undetected^g^ | **SMD 0.038**  (-0.024 to 0.101) | ⨁⨁◯◯  Low | CRITICAL |
| **Lysine** | | | | | | | | | |
| 3 | observational studies | Not serious | Not serious | Not serious | Not serious | Undetected^g^ | **SMD 0.054**  (-0.014 to 0.121) | ⨁⨁◯◯  Low | CRITICAL |
| **Methionine** | | | | | | | | | |
| 4 | observational studies | Not serious | Serious^b^ | Not serious | Not serious | Undetected^g^ | **SMD -0.396**  (-0.817 to 0.025) | ⨁◯◯◯  Very low | CRITICAL |
| **Ornithine** | | | | | | | | | |
| 2 | observational studies | Not serious | Not serious | Not serious | Not serious | Undetected^g^ | **SMD 0.010**  (-0.076 to 0.096) | ⨁⨁◯◯  Low | CRITICAL |
| **Proline** | | | | | | | | | |
| 4 | observational studies | Not serious | Not serious | Not serious | Not serious | Undetected^g^ | **SMD 0.062**  (0.035 to 0.088) | ⨁⨁◯◯  Low | CRITICAL |
| **Threonine** | | | | | | | | | |
| 3 | observational studies | Not serious | Not serious | Not serious | Not serious | Undetected^g^ | **SMD 0.008**  (-0.051 to 0.067) | ⨁⨁◯◯  Low | CRITICAL |
| **Tryptophan** | | | | | | | | | |
| 3 | observational studies | Not serious | Serious^b^ | Not serious | Not serious | Undetected^g^ | **SMD -0.342**  (-0.838 to 0.154) | ⨁◯◯◯  Very low | CRITICAL |
| **Tyrosine** | | | | | | | | | |
| 3 | observational studies | Not serious | Not serious | Not serious | Not serious | Undetected^g^ | **SMD -0.042**  (-0.206 to 0.123) | ⨁⨁◯◯  Low | CRITICAL |
| **Valine** | | | | | | | | | |
| 3 | observational studies | Not serious | Not serious | Not serious | Not serious | Undetected^g^ | **SMD 0.041**  (-0.022 to 0.104) | ⨁⨁◯◯  Low | CRITICAL |
| **Creatinine** | | | | | | | | | |
| 3 | observational studies | Not serious | Not serious | Not serious | Not serious | Undetected^g^ | **SMD -0.027**  (-0.087 to 0.033) | ⨁⨁◯◯  Low | CRITICAL |
| **Kynurenine** | | | | | | | | | |
| 2 | observational studies | Not serious | Serious^b^ | Not serious | Not serious | Undetected^g^ | **SMD -0.311**  (-0.926 to 0.304) | ⨁◯◯◯  Very low | CRITICAL |
| **Formate** | | | | | | | | | |
| 2 | observational studies | Not serious | Serious^b^ | Not serious | Serious^f^ | Undetected^g^ | **SMD 8.954**  (-8.813 to 26.721) | ⨁◯◯◯  Very low | CRITICAL |
| **Pyruvate** | | | | | | | | | |
| 2 | observational studies | Not serious | Serious^b^ | Not serious | Serious^f^ | Undetected^g^ | **SMD -0.738**  (-2.516 to 1.040) | ⨁◯◯◯  Very low | CRITICAL |
| **Asymmetric dimethylarginine** | | | | | | | | | |
| 2 | observational studies | Not serious | Serious^a^ | Not serious | Not serious | Undetected^g^ | **SMD 0.135**  (-0.255 to 0.524) | ⨁◯◯◯  Very low | CRITICAL |
| **Estrone-sulfate** | | | | | | | | | |
| 2 | observational studies | Not serious | Serious^d^ | Not serious | Not serious | Undetected^g^ | **SMD 0.530**  (0.169 to 0.893) | ⨁◯◯◯  Very low | CRITICAL |
| **Estrone** |  |  |  |  |  |  |  |  |  |
| 2 | observational studies | Not serious | Not serious | Not serious | Not serious | Undetected^g^ | **SMD 0.882**  (0.655 to 1.108) | ⨁⨁◯◯  Low | CRITICAL |
| **Estradiol** | | | | | | | | | |
| 2 | observational studies | Not serious | Serious^d^ | Not serious | Not serious | Undetected^g^ | **SMD 0.544**  (0.114 to 0.974) | ⨁◯◯◯  Very low | CRITICAL |
| **SM (OH) C14:1** | | | | | | | | | |
| 2 | observational studies | Not serious | Not serious | Not serious | Not serious | Undetected^g^ | **SMD 0.005**  (-0.059 to 0.070) | ⨁⨁◯◯  Low | CRITICAL |
| **SM (OH) C16:1** | | | | | | | | | |
| 2 | observational studies | Not serious | Not serious | Not serious | Not serious | Undetected^g^ | **SMD 0.052**  (-0.012 to 0.116) | ⨁⨁◯◯  Low | CRITICAL |
| **SM (OH) C22:1** | | | | | | | | | |
| 2 | observational studies | Not serious | Serious^d^ | Not serious | Not serious | Undetected^g^ | **SMD -0.046**  (-0.290 to 0.198) | ⨁◯◯◯  Very low | CRITICAL |
| **SM (OH) C22:2** | | | | | | | | | |
| 2 | observational studies | Not serious | Serious^d^ | Not serious | Not serious | Undetected^g^ | **SMD -0.104**  (-0.402 to 0.194) | ⨁◯◯◯  Very low | CRITICAL |
| **SM (OH) C24:1** | | | | | | | | | |
| 2 | observational studies | Not serious | Not serious | Not serious | Not serious | Undetected^g^ | **SMD 0.010**  (-0.059 to 0.080) | ⨁⨁◯◯  Low | CRITICAL |
| **SM C16:0** | | | | | | | | | |
| 2 | observational studies | Not serious | Not serious | Not serious | Not serious | Undetected^g^ | **SMD 0.011**  (-0.048 to 0.070) | ⨁⨁◯◯  Low | CRITICAL |
| **SM C16:1** | | | | | | | | | |
| 2 | observational studies | Not serious | Not serious | Not serious | Not serious | Undetected^g^ | **SMD -0.011**  (-0.072 to 0.050) | ⨁⨁◯◯  Low | CRITICAL |
| **SM C18:0** | | | | | | | | | |
| 2 | observational studies | Not serious | Serious^d^ | Not serious | Not serious | Undetected^g^ | **SMD 0.172**  (-0.077 to 0.421) | ⨁◯◯◯  Very low | CRITICAL |
| **SM C18:1** | | | | | | | | | |
| 2 | observational studies | Not serious | Not serious | Not serious | Not serious | Undetected^g^ | **SMD 0.096**  (-0.087 to 0.279) | ⨁⨁◯◯  Low | CRITICAL |
| **SM C20:2** | | | | | | | | | |
| 2 | observational studies | Not serious | Not serious | Not serious | Not serious | Undetected^g^ | **SMD 0.034**  (-0.044 to 0.111) | ⨁⨁◯◯  Low | CRITICAL |
| **SM C24:0** |  |  |  |  |  |  |  |  |  |
| 2 | observational studies | Not serious | Serious^d^ | Not serious | Not serious | Undetected^g^ | **SMD -0.088**  (-0.339 to 0.163) | ⨁◯◯◯  Very low | CRITICAL |
| **SM C24:1** |  |  |  |  |  |  |  |  |  |
| 2 | observational studies | Not serious | Not serious | Not serious | Not serious | Undetected^g^ | **SMD 0.044**  (-0.021 to 0.109) | ⨁⨁◯◯  Low | CRITICAL |
| **SM C26:0** | | | | | | | | | |
| 2 | observational studies | Not serious | Not serious | Not serious | Not serious | Undetected^g^ | **SMD 0.004**  (-0.105 to 0.113) | ⨁⨁◯◯  Low | CRITICAL |
| **SM C26:1** | | | | | | | | | |
| 2 | observational studies | Not serious | Not serious | Not serious | Not serious | Undetected^g^ | **SMD 0.019**  (-0.086 to 0.125) | ⨁⨁◯◯  Low | CRITICAL |
| **C0** | | | | | | | | | |
| 3 | observational studies | Not serious | Not serious | Not serious | Not serious | Undetected^g^ | **SMD -0.076**  (-0.167 to 0.014) | ⨁⨁◯◯  Low | CRITICAL |
| **C10** | | | | | | | | | |
| 3 | observational studies | Not serious | Serious^b^ | Not serious | Not serious | Undetected^g^ | **SMD 0.092**  (-0.027 to 0.211) | ⨁◯◯◯  Very low | CRITICAL |
| **C14:1** | | | | | | | | | |
| 2 | observational studies | Not serious | Serious^b^ | Not serious | Not serious | Undetected^g^ | **SMD 0.340**  (-0.373 to 1.053) | ⨁◯◯◯  Very low | CRITICAL |
| **C14:2** | | | | | | | | | |
| 2 | observational studies | Not serious | Serious^b^ | Not serious | Not serious | Undetected^g^ | **SMD 0.376**  (-0.377 to 1.129) | ⨁◯◯◯  Very low | CRITICAL |
| **C16** | | | | | | | | | |
| 2 | observational studies | Not serious | Serious^b^ | Not serious | Not serious | Undetected^g^ | **SMD 0.386**  (-0.347 to 1.119) | ⨁◯◯◯  Very low | CRITICAL |
| **C18** | | | | | | | | | |
| 2 | observational studies | Not serious | Not serious | Not serious | Not serious | Undetected^g^ | **SMD -0.013**  (-0.083 to 0.057) | ⨁⨁◯◯  Low | CRITICAL |
| **C18:1** | | | | | | | | | |
| 2 | observational studies | Not serious | Serious^b^ | Not serious | Not serious | Undetected^g^ | **SMD 0.490**  (-0.459 to 1.439) | ⨁◯◯◯  Very low | CRITICAL |
| **C18:2** | | | | | | | | | |
| 2 | observational studies | Not serious | Serious^b^ | Not serious | Not serious | Undetected^g^ | **SMD 0.488**  (-0.465 to 1.441) | ⨁◯◯◯  Very low | CRITICAL |
| **C2** | | | | | | | | | |
| 2 | observational studies | Not serious | Serious^b^ | Not serious | Not serious | Undetected^g^ | **SMD 0.331**  (-0.335 to 0.998) | ⨁◯◯◯  Very low | CRITICAL |
| **C3** | | | | | | | | | |
| 2 | observational studies | Not serious | Serious^d^ | Not serious | Not serious | Undetected^g^ | **SMD -0.096**  (-0.380 to 0.188) | ⨁◯◯◯  Very low | CRITICAL |
| **C4** | | | | | | | | | |
| 3 | observational studies | Not serious | Serious^c^ | Not serious | Not serious | Undetected^g^ | **SMD -0.045**  (-0.090 to 0.001) | ⨁◯◯◯  Very low | CRITICAL |
| **C5** | | | | | | | | | |
| 2 | observational studies | Not serious | Not serious | Not serious | Not serious | Undetected^g^ | **SMD 0.019**  (-0.113 to 0.152) | ⨁⨁◯◯  Low | CRITICAL |
| **C6 (C4:1-DC)** | | | | | | | | | |
| 2 | observational studies | Not serious | Serious^b^ | Not serious | Not serious | Undetected^g^ | **SMD 0.378**  (-0.371 to 1.127) | ⨁◯◯◯  Very low | CRITICAL |
| **LysoPC a C16:0** | | | | | | | | | |
| 2 | observational studies | Not serious | Serious^d^ | Not serious | Not serious | Undetected^g^ | **SMD -0.132**  (-0.452 to 0.188) | ⨁◯◯◯  Very low | CRITICAL |
| **LysoPC a C16:1** | | | | | | | | | |
| 2 | observational studies | Not serious | Not serious | Not serious | Not serious | Undetected^g^ | **SMD -0.002**  (-0.078 to 0.074) | ⨁⨁◯◯  Low | CRITICAL |
| **LysoPC a C17:0** | | | | | | | | | |
| 2 | observational studies | Not serious | Serious^b^ | Not serious | Not serious | Undetected^g^ | **SMD -0.263**  (-0.830 to 0.305) | ⨁◯◯◯  Very low | CRITICAL |
| **LysoPC a C18:0** | | | | | | | | | |
| 2 | observational studies | Not serious | Serious^c^ | Not serious | Not serious | Undetected^g^ | **SMD -0.192**  (-0.563 to 0.179) | ⨁◯◯◯  Very low | CRITICAL |
| **LysoPC a C18:1** | | | | | | | | | |
| 2 | observational studies | Not serious | Serious^c^ | Not serious | Not serious | Undetected^g^ | **SMD -0.231**  (-0.686 to 0.224) | ⨁◯◯◯  Very low | CRITICAL |
| **LysoPC a C18:2** | | | | | | | | | |
| 3 | observational studies | Not serious | Serious^c^ | Not serious | Not serious | Undetected^g^ | **SMD -0.077**  (-0.160 to 0.006) | ⨁◯◯◯  Very low | CRITICAL |
| **LysoPC a C20:3** | | | | | | | | | |
| 2 | observational studies | Not serious | Not serious | Not serious | Not serious | Undetected^g^ | **SMD 0.012**  (-0.050 to 0.074) | ⨁⨁◯◯  Low | CRITICAL |
| **LysoPC a C20:4** | | | | | | | | | |
| 2 | observational studies | Not serious | Not serious | Not serious | Not serious | Undetected^g^ | **SMD 0.010**  (-0.053 to 0.073) | ⨁⨁◯◯  Low | CRITICAL |
| **PC aa C28:1** | | | | | | | | | |
| 3 | observational studies | Not serious | Not serious | Not serious | Not serious | Undetected^g^ | **SMD -0.035**  (-0.010 to 0.081) | ⨁⨁◯◯  Low | CRITICAL |
| **PC aa C30:0** | | | | | | | | | |
| 2 | observational studies | Not serious | Not serious | Not serious | Not serious | Undetected^g^ | **SMD -0.029**  (-0.173 to 0.116) | ⨁⨁◯◯  Low | CRITICAL |
| **PC aa C32:0** | | | | | | | | | |
| 2 | observational studies | Not serious | Not serious | Not serious | Not serious | Undetected^g^ | **SMD 0.019**  (-0.054 to 0.092) | ⨁⨁◯◯  Low | CRITICAL |
| **PC aa C32:1** | | | | | | | | | |
| 2 | observational studies | Not serious | Not serious | Not serious | Not serious | Undetected^g^ | **SMD 0.010**  (-0.059 to 0.079) | ⨁⨁◯◯  Low | CRITICAL |
| **PC aa C32:2** | | | | | | | | | |
| 3 | observational studies | Not serious | Not serious | Not serious | Not serious | Undetected^g^ | **SMD -0.047**  (-0.085 to -0.009) | ⨁⨁◯◯  Low | CRITICAL |
| **PC aa C32:3** | | | | | | | | | |
| 2 | observational studies | Not serious | Not serious | Not serious | Not serious | Undetected^g^ | **SMD -0.009**  (-0.097 to 0.080) | ⨁⨁◯◯  Low | CRITICAL |
| **PC aa C34:1** | | | | | | | | | |
| 2 | observational studies | Not serious | Not serious | Not serious | Not serious | Undetected^g^ | **SMD -0.009**  (-0.115 to 0.097) | ⨁⨁◯◯  Low | CRITICAL |
| **PC aa C34:2** | | | | | | | | | |
| 2 | observational studies | Not serious | Not serious | Not serious | Not serious | Undetected^g^ | **SMD -0.012**  (-0.095 to 0.071) | ⨁⨁◯◯  Low | CRITICAL |
| **PC aa C34:3** | | | | | | | | | |
| 2 | observational studies | Not serious | Not serious | Not serious | Not serious | Undetected^g^ | **SMD -0.061**  (-0.216 to 0.095) | ⨁⨁◯◯  Low | CRITICAL |
| **PC aa C34:4** | | | | | | | | | |
| 2 | observational studies | Not serious | Serious^d^ | Not serious | Not serious | Undetected^g^ | **SMD -0.073**  (-0.320 to 0.173) | ⨁◯◯◯  Very low | CRITICAL |
| **PC aa C36:0** | | | | | | | | | |
| 3 | observational studies | Not serious | Serious^c^ | Not serious | Not serious | Undetected^g^ | **SMD -0.101**  (-0.225 to 0.023) | ⨁◯◯◯  Very low | CRITICAL |
| **PC aa C36:1** | | | | | | | | | |
| 3 | observational studies | Not serious | Serious^c^ | Not serious | Not serious | Undetected^g^ | **SMD -0.045**  (-0.122 to 0.032) | ⨁◯◯◯  Very low | CRITICAL |
| **PC aa C36:2** | | | | | | | | | |
| 2 | observational studies | Not serious | Serious^d^ | Not serious | Not serious | Undetected^g^ | **SMD -0.146**  (-0.439 to 0.147) | ⨁◯◯◯  Very low | CRITICAL |
| **PC aa C36:3** | | | | | | | | | |
| 2 | observational studies | Not serious | Serious^d^ | Not serious | Not serious | Undetected^g^ | **SMD -0.158**  (-0.506 to 0.190) | ⨁◯◯◯  Very low | CRITICAL |
| **PC aa C36:4** | | | | | | | | | |
| 2 | observational studies | Not serious | Not serious | Not serious | Not serious | Undetected^g^ | **SMD 0.015**  (-0.053 to 0.082) | ⨁⨁◯◯  Low | CRITICAL |
| **PC aa C36:5** | | | | | | | | | |
| 2 | observational studies | Not serious | Serious^b^ | Not serious | Not serious | Undetected^g^ | **SMD -0.214**  (-0.805 to 0.378) | ⨁◯◯◯  Very low | CRITICAL |
| **PC aa C36:6** | | | | | | | | | |
| 2 | observational studies | Not serious | Serious^b^ | Not serious | Not serious | Undetected^g^ | **SMD -0.285**  (-0.967 to 0.397) | ⨁◯◯◯  Very low | CRITICAL |
| **PC aa C38:0** | | | | | | | | | |
| 2 | observational studies | Not serious | Serious^b^ | Not serious | Not serious | Undetected^g^ | **SMD -0.194**  (-0.630 to 0.242) | ⨁◯◯◯  Very low | CRITICAL |
| **PC aa C38:3** | | | | | | | | | |
| 2 | observational studies | Not serious | Not serious | Not serious | Not serious | Undetected^g^ | **SMD 0.017**  (-0.049 to 0.082) | ⨁⨁◯◯  Low | CRITICAL |
| **PC aa C38:4** | | | | | | | | | |
| 2 | observational studies | Not serious | Not serious | Not serious | Not serious | Undetected^g^ | **SMD 0.010**  (-0.054 to 0.073) | ⨁⨁◯◯  Low | CRITICAL |
| **PC aa C38:5** | | | | | | | | | |
| 2 | observational studies | Not serious | Serious^b^ | Not serious | Not serious | Undetected^g^ | **SMD -0.234**  (-0.797 to 0.330) | ⨁◯◯◯  Very low | CRITICAL |
| **PC aa C38:6** | | | | | | | | | |
| 2 | observational studies | Not serious | Serious^b^ | Not serious | Not serious | Undetected^g^ | **SMD -0.115**  (-0.493 to 0.263) | ⨁◯◯◯  Very low | CRITICAL |
| **PC aa C40:2** | | | | | | | | | |
| 2 | observational studies | Not serious | Serious^b^ | Not serious | Not serious | Undetected^g^ | **SMD -0.187**  (-0.605 to 0.232) | ⨁◯◯◯  Very low | CRITICAL |
| **PC aa C40:3** | | | | | | | | | |
| 2 | observational studies | Not serious | Serious^b^ | Not serious | Not serious | Undetected^g^ | **SMD -0.145**  (-0.545 to 0.254) | ⨁◯◯◯  Very low | CRITICAL |
| **PC aa C40:4** | | | | | | | | | |
| 2 | observational studies | Not serious | Not serious | Not serious | Not serious | Undetected^g^ | **SMD -0.011**  (-0.076 to 0.053) | ⨁⨁◯◯  Low | CRITICAL |
| **PC aa C40:5** | | | | | | | | | |
| 2 | observational studies | Not serious | Not serious | Not serious | Not serious | Undetected^g^ | **SMD -0.003**  (-0.068 to 0.063) | ⨁⨁◯◯  Low | CRITICAL |
| **PC aa C40:6** | | | | | | | | | |
| 2 | observational studies | Not serious | Not serious | Not serious | Not serious | Undetected^g^ | **SMD 0.011**  (-0.090 to 0.111) | ⨁⨁◯◯  Low | CRITICAL |
| **PC aa C42:0** | | | | | | | | | |
| 2 | observational studies | Not serious | Not serious | Not serious | Not serious | Undetected^g^ | **SMD 0.049**  (-0.014 to 0.111) | ⨁⨁◯◯  Low | CRITICAL |
| **PC aa C42:1** | | | | | | | | | |
| 2 | observational studies | Not serious | Not serious | Not serious | Not serious | Undetected^g^ | **SMD 0.006**  (-0.057 to 0.069) | ⨁⨁◯◯  Low | CRITICAL |
| **PC aa C42:2** | | | | | | | | | |
| 2 | observational studies | Not serious | Serious^b^ | Not serious | Not serious | Undetected^g^ | **SMD -0.271**  (-0.813 to 0.272) | ⨁◯◯◯  Very low | CRITICAL |
| **PC aa C42:4** | | | | | | | | | |
| 2 | observational studies | Not serious | Not serious | Not serious | Not serious | Undetected^g^ | **SMD -0.055**  (-0.264 to 0.153) | ⨁⨁◯◯  Low | CRITICAL |
| **PC aa C42:5** | | | | | | | | | |
| 3 | observational studies | Not serious | Serious^b^ | Not serious | Not serious | Undetected^g^ | **SMD -0.240**  (-0.634 to 0.153) | ⨁◯◯◯  Very low | CRITICAL |
| **PC aa C42:6** | | | | | | | | | |
| 3 | observational studies | Not serious | Serious^b^ | Not serious | Not serious | Undetected^g^ | **SMD -0.305**  (-0.744 to 0.134) | ⨁◯◯◯  Very low | CRITICAL |
| **PC ae C30:0** | | | | | | | | | |
| 2 | observational studies | Not serious | Not serious | Not serious | Not serious | Undetected^g^ | **SMD -0.031**  (-0.217 to 0.154) | ⨁⨁◯◯  Low | CRITICAL |
| **PC ae C30:2** | | | | | | | | | |
| 2 | observational studies | Not serious | Not serious | Not serious | Not serious | Undetected^g^ | **SMD 0.046**  (-0.019 to 0.110) | ⨁⨁◯◯  Low | CRITICAL |
| **PC ae C32:1** | | | | | | | | | |
| 2 | observational studies | Not serious | Not serious | Not serious | Not serious | Undetected^g^ | **SMD 0.009**  (-0.062 to 0.080) | ⨁⨁◯◯  Low | CRITICAL |
| **PC ae C32:2** | | | | | | | | | |
| 2 | observational studies | Not serious | Not serious | Not serious | Not serious | Undetected^g^ | **SMD 0.003**  (-0.105 to 0.111) | ⨁⨁◯◯  Low | CRITICAL |
| **PC ae C34:0** | | | | | | | | | |
| 2 | observational studies | Not serious | Serious^b^ | Not serious | Not serious | Undetected^g^ | **SMD -0.107**  (-0.480 to 0.266) | ⨁◯◯◯  Very low | CRITICAL |
| **PC ae C34:1** | | | | | | | | | |
| 2 | observational studies | Not serious | Serious^d^ | Not serious | Not serious | Undetected^g^ | **SMD -0.108**  (-0.402 to 0.186) | ⨁◯◯◯  Very low | CRITICAL |
| **PC ae C34:2** | | | | | | | | | |
| 2 | observational studies | Not serious | Serious^b^ | Not serious | Not serious | Undetected^g^ | **SMD -0.230**  (-0.667 to 0.207) | ⨁◯◯◯  Very low | CRITICAL |
| **PC ae C34:3** | | | | | | | | | |
| 2 | observational studies | Not serious | Serious^c^ | Not serious | Not serious | Undetected^g^ | **SMD -0.228**  (-0.642 to 0.185) | ⨁◯◯◯  Very low | CRITICAL |
| **PC ae C36:0** | | | | | | | | | |
| 2 | observational studies | Not serious | Serious^a^ | Not serious | Not serious | Undetected^g^ | **SMD -0.070**  (-0.365 to 0.224) | ⨁◯◯◯  Very low | CRITICAL |
| **PC ae C36:1** | | | | | | | | | |
| 2 | observational studies | Not serious | Serious^b^ | Not serious | Not serious | Undetected^g^ | **SMD -0.218**  (-0.735 to 0.300) | ⨁◯◯◯  Very low | CRITICAL |
| **PC ae C36:2** | | | | | | | | | |
| 2 | observational studies | Not serious | Serious^b^ | Not serious | Not serious | Undetected^g^ | **SMD -0.218**  (-0.659 to 0.224) | ⨁◯◯◯  Very low | CRITICAL |
| **PC ae C36:3** | | | | | | | | | |
| 2 | observational studies | Not serious | Serious^b^ | Not serious | Not serious | Undetected^g^ | **SMD -0.261**  (-0.746 to 0.224) | ⨁◯◯◯  Very low | CRITICAL |
| **PC ae C36:4** | | | | | | | | | |
| 2 | observational studies | Not serious | Not serious | Not serious | Not serious | Undetected^g^ | **SMD -0.025**  (-0.095 to 0.044) | ⨁⨁◯◯  Low | CRITICAL |
| **PC ae C36:5** | | | | | | | | | |
| 2 | observational studies | Not serious | Serious^d^ | Not serious | Not serious | Undetected^g^ | **SMD -0.114**  (-0.392 to 0.165) | ⨁◯◯◯  Very low | CRITICAL |
| **PC ae C38:0** | | | | | | | | | |
| 2 | observational studies | Not serious | Serious^b^ | Not serious | Not serious | Undetected^g^ | **SMD -0.315**  (-1.041 to 0.411) | ⨁◯◯◯  Very low | CRITICAL |
| **PC ae C38:2** | | | | | | | | | |
| 2 | observational studies | Not serious | Serious^b^ | Not serious | Not serious | Undetected^g^ | **SMD -0.212**  (-0.654 to 0.230) | ⨁◯◯◯  Very low | CRITICAL |
| **PC ae C38:3** | | | | | | | | | |
| 2 | observational studies | Not serious | Not serious | Not serious | Not serious | Undetected^g^ | **SMD 0.020**  (-0.047 to 0.087) | ⨁⨁◯◯  Low | CRITICAL |
| **PC ae C38:4** | | | | | | | | | |
| 2 | observational studies | Not serious | Not serious | Not serious | Not serious | Undetected^g^ | **SMD 0.006**  (-0.060 to 0.072) | ⨁⨁◯◯  Low | CRITICAL |
| **PC ae C38:5** | | | | | | | | | |
| 2 | observational studies | Not serious | Serious^a^ | Not serious | Not serious | Undetected^g^ | **SMD -0.134**  (-0.454 to 0.187) | ⨁◯◯◯  Very low | CRITICAL |
| **PC ae C38:6** | | | | | | | | | |
| 2 | observational studies | Not serious | Serious^b^ | Not serious | Not serious | Undetected^g^ | **SMD -0.239**  (-0.777 to 0.300) | ⨁◯◯◯  Very low | CRITICAL |
| **PC ae C40:1** | | | | | | | | | |
| 2 | observational studies | Not serious | Serious^b^ | Not serious | Not serious | Undetected^g^ | **SMD -0.420**  (-1.273 to 0.434) | ⨁◯◯◯  Very low | CRITICAL |
| **PC ae C40:2** | | | | | | | | | |
| 2 | observational studies | Not serious | Not serious | Not serious | Not serious | Undetected^g^ | **SMD 0.052**  (-0.011 to 0.114) | ⨁⨁◯◯  Low | CRITICAL |
| **PC ae C40:3** | | | | | | | | | |
| 2 | observational studies | Not serious | Not serious | Not serious | Not serious | Undetected^g^ | **SMD 0.024**  (-0.040 to 0.088) | ⨁⨁◯◯  Low | CRITICAL |
| **PC ae C40:4** | | | | | | | | | |
| 2 | observational studies | Not serious | Not serious | Not serious | Not serious | Undetected^g^ | **SMD 0.023**  (-0.039 to 0.085) | ⨁⨁◯◯  Low | CRITICAL |
| **PC ae C40:5** | | | | | | | | | |
| 2 | observational studies | Not serious | Not serious | Not serious | Not serious | Undetected^g^ | **SMD -0.028**  (-0.212 to 0.157) | ⨁⨁◯◯  Low | CRITICAL |
| **PC ae C40:6** | | | | | | | | | |
| 2 | observational studies | Not serious | Serious^b^ | Not serious | Not serious | Undetected^g^ | **SMD -0.198**  (-0.683 to 0.288) | ⨁◯◯◯  Very low | CRITICAL |
| **PC ae C42:1** | | | | | | | | | |
| 2 | observational studies | Not serious | Serious^b^ | Not serious | Not serious | Undetected^g^ | **SMD -0.239**  (-0.804 to 0.326) | ⨁◯◯◯  Very low | CRITICAL |
| **PC ae C42:2** | | | | | | | | | |
| 2 | observational studies | Not serious | Serious^b^ | Not serious | Not serious | Undetected^g^ | **SMD -0.254**  (-0.838 to 0.330) | ⨁◯◯◯  Very low | CRITICAL |
| **PC ae C42:3** | | | | | | | | | |
| 2 | observational studies | Not serious | Serious^b^ | Not serious | Not serious | Undetected^g^ | **SMD -0.118**  (-0.512 to 0.275) | ⨁◯◯◯  Very low | CRITICAL |
| **PC ae C42:4** | | | | | | | | | |
| 2 | observational studies | Not serious | Not serious | Not serious | Not serious | Undetected^g^ | **SMD 0.025**  (-0.034 to 0.083) | ⨁⨁◯◯  Low | CRITICAL |
| **PC ae C42:5** | | | | | | | | | |
| 2 | observational studies | Not serious | Not serious | Not serious | Not serious | Undetected^g^ | **SMD 0.041**  (-0.016 to 0.099) | ⨁⨁◯◯  Low | CRITICAL |
| **PC ae C44:3** | | | | | | | | | |
| 2 | observational studies | Not serious | Not serious | Not serious | Not serious | Undetected^g^ | **SMD 0.031**  (-0.032 to 0.093) | ⨁⨁◯◯  Low | CRITICAL |
| **PC ae C44:4** | | | | | | | | | |
| 2 | observational studies | Not serious | Not serious | Not serious | Not serious | Undetected^g^ | **SMD 0.039**  (-0.021 to 0.099) | ⨁⨁◯◯  Low | CRITICAL |
| **PC ae C44:5** | | | | | | | | | |
| 2 | observational studies | Not serious | Not serious | Not serious | Not serious | Undetected^g^ | **SMD 0.060**  (-0.001 to 0.120) | ⨁⨁◯◯  Low | CRITICAL |
| **PC ae C44:6** | | | | | | | | | |
| 2 | observational studies | Not serious | Not serious | Not serious | Not serious | Undetected^g^ | **SMD 0.043**  (-0.016 to 0.103) | ⨁⨁◯◯  Low | CRITICAL |

Abbreviations: C0, carnitine; C10, decanoylcarnitine; C14:1, tetradecenoylcarnitine; C14:2, tetradecadienoylcarnitine; C16, hexadecenoylcarnitine; C18, 3-hydroxylhexadecanoylcarnitine; C18:1, octadecenoylcarnitine; C18:2, octadecadienoylcarnitine; C2, acetylcarnitine; C3, propionylcarnitine; C4, malonylcarnitine; C5, methylmalonylcarnitine; C6, glutarylcarnitine; LysoPC, lysophosphatidylcholine; PC, Phosphatidylcholine; SM, sphingomyelin; SMD, standardized mean difference.

^a^ I^2^ > 50% and *p*-value ≤ 0.10 (heterogeneity was high), downgrade for a wide variation of point estimates across studies.

^b^ I^2^ > 50% and *p*-value ≤ 0.10 (heterogeneity was high), downgrade for a wide variation of point estimates across studies and minimal or no overlap of confidence intervals.

^c^ I^2^ > 50% and *p*-value ≤ 0.10 (heterogeneity was high), downgrade for minimal or no overlap of confidence intervals.

^d^ I^2^ > 50% and/or *p*-value ≤ 0.10 (heterogeneity was high), but no downgrade for similarity of point estimates and considerable overlap of confidence intervals.

^e^ Downgrade for small number of participants (n < 400).

^f^ Downgrade for small number of participants (n < 400) and wide confidence intervals around the estimate of the effect.

^g^ No downgrade for publication bias, as publication bias could not be assessed due to lack of power (< 10 data points included in our meta-analysis).

**Table S10: GRADE assessment for all outcomes of endometrial cancer metabolic pathways evaluated in this meta-analysis.**

| **Certainty assessment** | | | | | | | **Relative Effect (95% CI)** | **Certainty** | **Importance** |
| --- | --- | --- | --- | --- | --- | --- | --- | --- | --- |
| **№ of studies** | **Study design** | **Risk of bias** | **Inconsistency** | **Indirectness** | **Imprecision** | **Publication bias** |  |  |  |
| **Cholesterol hormone metabolism** | | | | | | | | | |
| 15 | observational studies | Not serious | Not serious | Not serious | Not serious | Not serious | **SMD 0.750**  (0.598 to 0.903) | ⨁⨁◯◯  Low | CRITICAL |
| **Choline metabolism** | | | | | | | | | |
| 3 | observational studies | Not serious | Serious^a^ | Not serious | Serious^d^ | Undetected^e^ | **SMD 2.929**  (0.858 to 5.001) | ⨁◯◯◯  Very low | CRITICAL |
| **Carbohydrate metabolism** | | | | | | | | | |
| 16 | observational studies | Not serious | Not serious^b^ | Not serious | Not serious | Serious^f^ | **SMD 0.762**  (0.333 to 1.191) | ⨁◯◯◯  Very low | CRITICAL |
| **Alanine, aspartate, and glutamate metabolism** | | | | | | | | | |
| 15 | observational studies | Not serious | Not serious | Not serious | Not serious | Not serious | **SMD -0.007**  (-0.064 to 0.050) | ⨁⨁◯◯  Low | CRITICAL |
| **BCAA metabolism** | | | | | | | | | |
| 10 | observational studies | Not serious | Not serious | Not serious | Not serious | Serious^f^ | **SMD 0.041**  (0.005 to 0.078) | ⨁◯◯◯  Very low | CRITICAL |
| **AAA metabolism** | | | | | | | | | |
| 7 | observational studies | Not serious | Not serious | Not serious | Not serious | Undetected^e^ | **SMD 0.016**  (-0.028 to 0.060) | ⨁⨁◯◯  Low | CRITICAL |
| **Arginine and proline metabolism, Urea cycle** | | | | | | | | | |
| 21 | observational studies | Not serious | Not serious | Not serious | Not serious | Serious^f^ | **SMD -0.009**  (-0.051 to 0.032) | ⨁◯◯◯  Very low | CRITICAL |
| **Glycine, serine, and threonine metabolism** | | | | | | | | | |
| 10 | observational studies | Not serious | Not serious | Not serious | Not serious | Not serious | **SMD -0.057**  (-0.128 to 0.013) | ⨁⨁◯◯  Low | CRITICAL |
| **Histidine metabolism** | | | | | | | | | |
| 4 | observational studies | Not serious | Not serious | Not serious | Not serious | Undetected^e^ | **SMD -0.097**  (-0.318 to 0.124) | ⨁⨁◯◯  Low | CRITICAL |
| **Lysine metabolism** | | | | | | | | | |
| 4 | observational studies | Not serious | Not serious | Not serious | Not serious | Undetected^e^ | **SMD 0.056**  (-0.011 to 0.122) | ⨁⨁◯◯  Low | CRITICAL |
| **Methionine metabolism** | | | | | | | | | |
| 5 | observational studies | Not serious | Serious^a^ | Not serious | Not serious | Undetected^e^ | **SMD -0.227**  (-0.582 to 0.127) | ⨁◯◯◯  Very low | CRITICAL |
| **Tryptophan metabolism** | | | | | | | | | |
| 6 | observational studies | Not serious | Serious^a^ | Not serious | Not serious | Undetected^e^ | **SMD -0.164**  (-0.315 to -0.014) | ⨁◯◯◯  Very low | CRITICAL |
| **Taurine and hypotaurine metabolism** | | | | | | | | | |
| 2 | observational studies | Not serious | Not serious | Not serious | Not serious | Undetected^e^ | **SMD 0.029**  (-0.069 to 0.127) | ⨁⨁◯◯  Low | CRITICAL |
| **Acylcarnitine metabolism** | | | | | | | | | |
| 33 | observational studies | Not serious | Not serious^c^ | Not serious | Not serious | Serious^f^ | **SMD 0.092**  (0.046 to 0.137) | ⨁◯◯◯  Very low | CRITICAL |
| **Glycerophospholipid metabolism** | | | | | | | | | |
| 170 | observational studies | Not serious | Not serious | Not serious | Not serious | Serious^f^ | **SMD -0.030**  (-0.045 to -0.015) | ⨁◯◯◯  Very low | CRITICAL |
| **Sphingolipid metabolism** | | | | | | | | | |
| 29 | observational studies | Not serious | Not serious | Not serious | Not serious | Not serious | **SMD 0.025**  (0.008 to 0.042) | ⨁⨁◯◯  Low | CRITICAL |

Abbreviations: AAA, aromatic amino acid; BCAA, branched-chain amino acid; SMD, standardized mean difference.

^a^ I^2^ > 50% and *p*-value ≤ 0.10 (heterogeneity was high), downgrade for a wide variation of point estimates across studies and minimal or no overlap of confidence intervals.

^b^ I^2^ > 50% and *p*-value ≤ 0.10 (heterogeneity was high), this was explained by the biological sample type and control type. Therefore, we did not downgrade for serious inconsistency.

^b^ I^2^ > 50% and *p*-value ≤ 0.10 (heterogeneity was high), this was explained by the biological sample type, control type and sample size. Therefore, we did not downgrade for serious inconsistency.

^d^ Downgrade for small number of participants (n < 400) and wide confidence intervals around the estimate of the effect.

^e^ No downgrade for publication bias, as publication bias could not be assessed due to lack of power (< 10 data points included in our meta-analysis).

^f^ Downgrade for small-study effects.


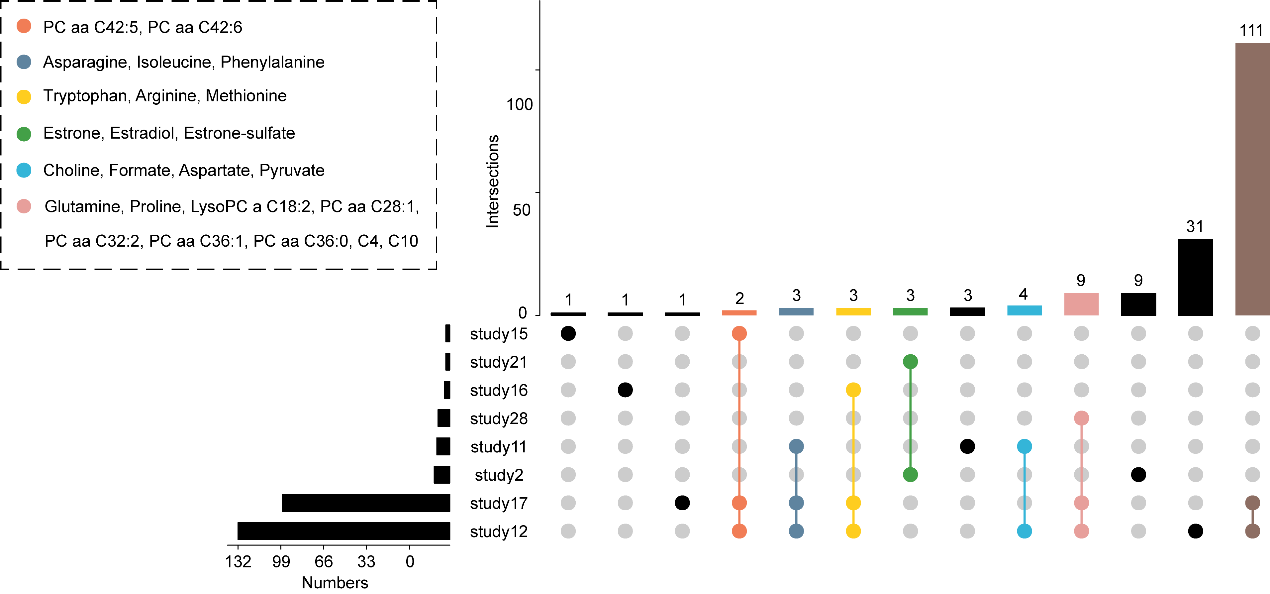


**Figure S1: Upset plot illustrating the overlap of endometrial cancer metabolites identified across studies.** C4, Butyrylcarnitine; C10, Decanoylcarnitine; LysoPC, lysophosphatidylcholine; PC, Phosphatidylcholine.


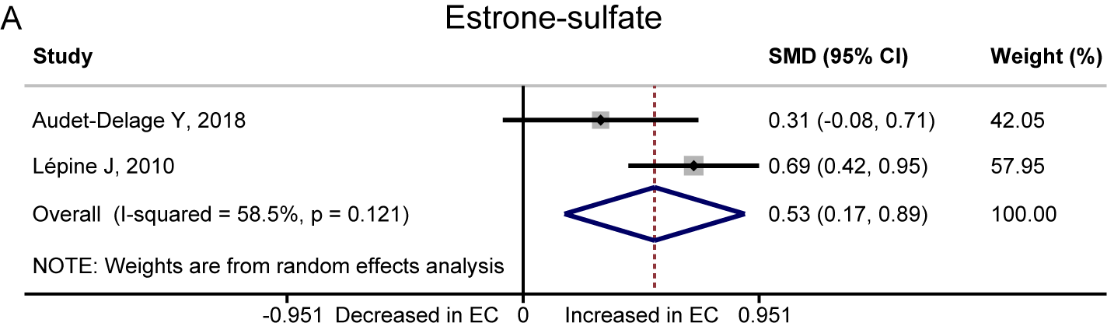


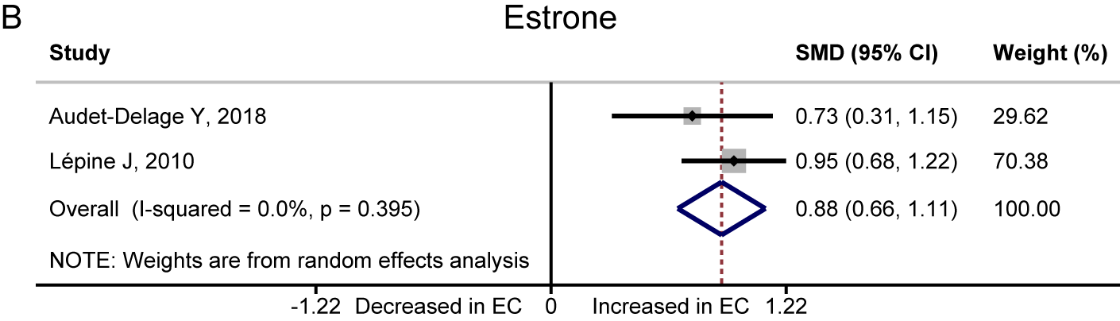


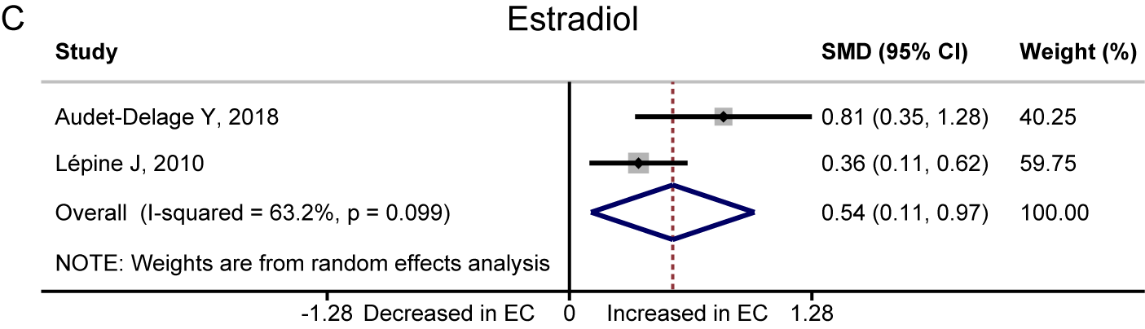


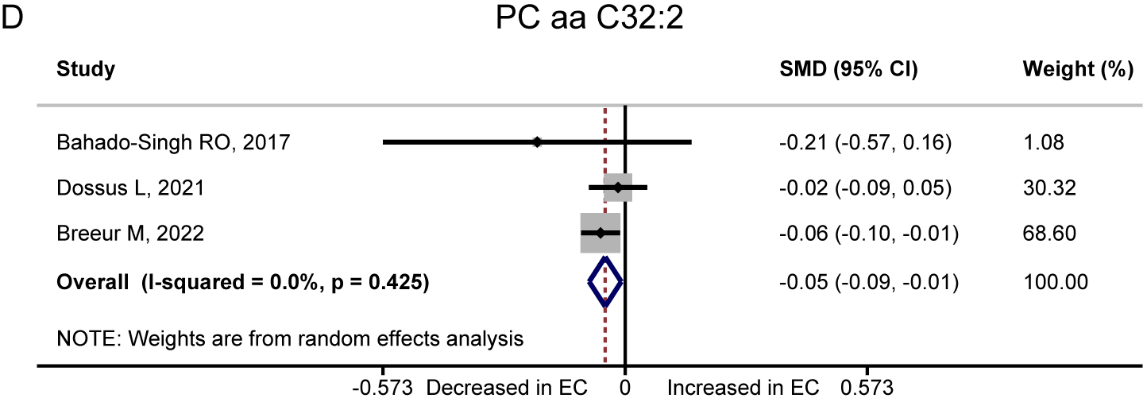


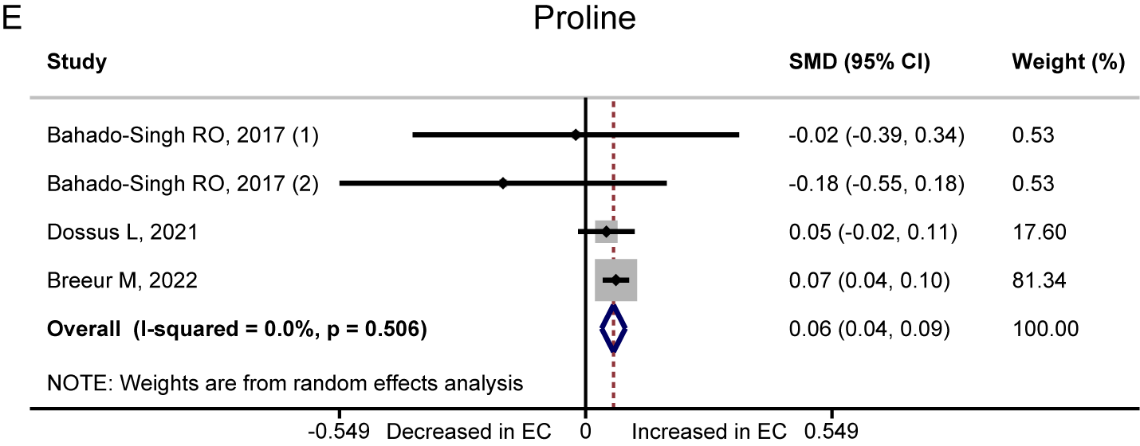


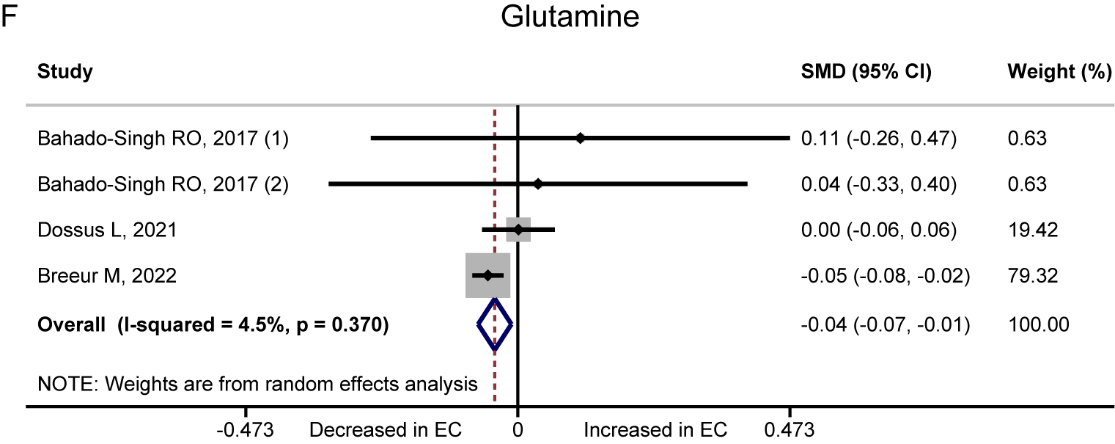


**Figure S2: Forest plots assessing changes in the levels of biomarkers associated with endometrial cancer. (A) Estrone-sulfate; (B) Estrone; (C) Estradiol; (D) PC aa C32:2; (E) Proline; (F) Glutamine.** CI, confidence intervals; PC, Phosphatidylcholine; SMD, standardized mean difference.

**
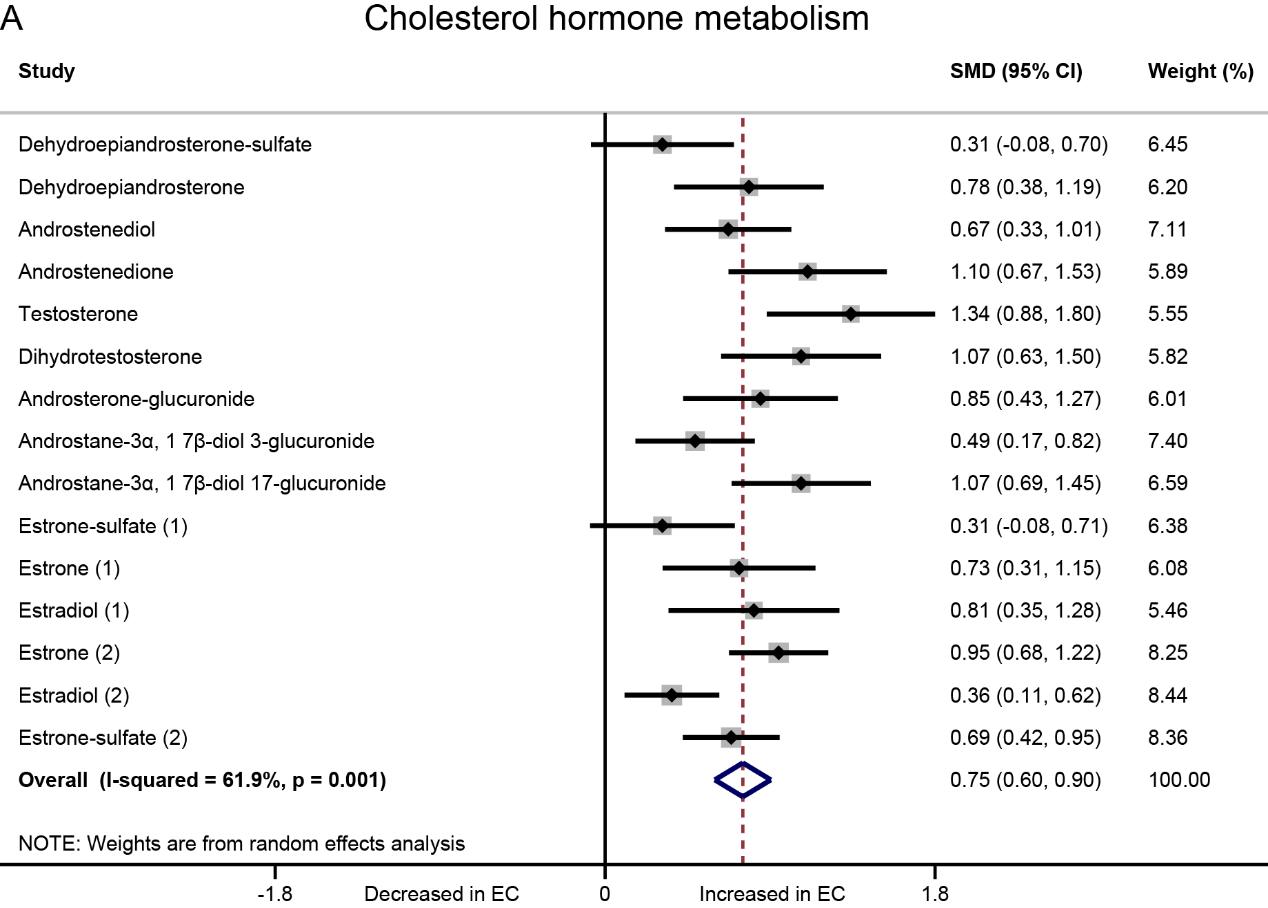
**

**
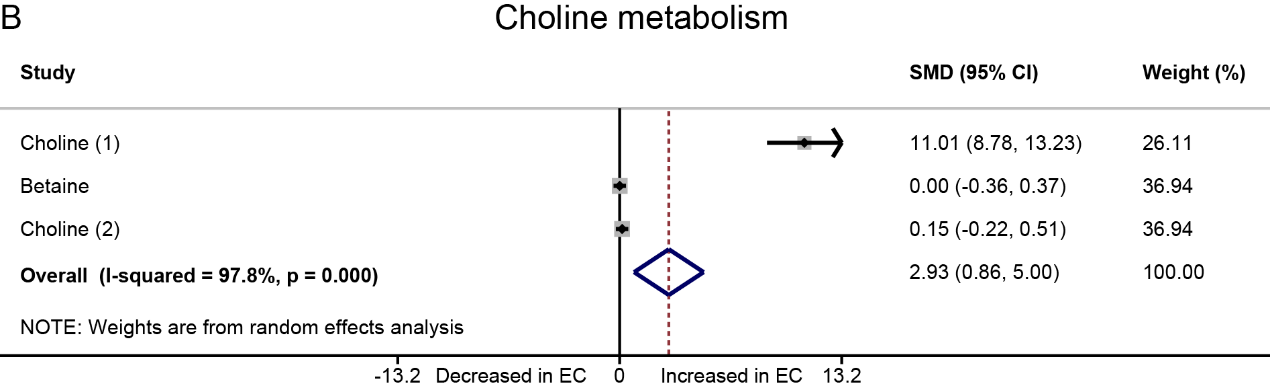
**

**
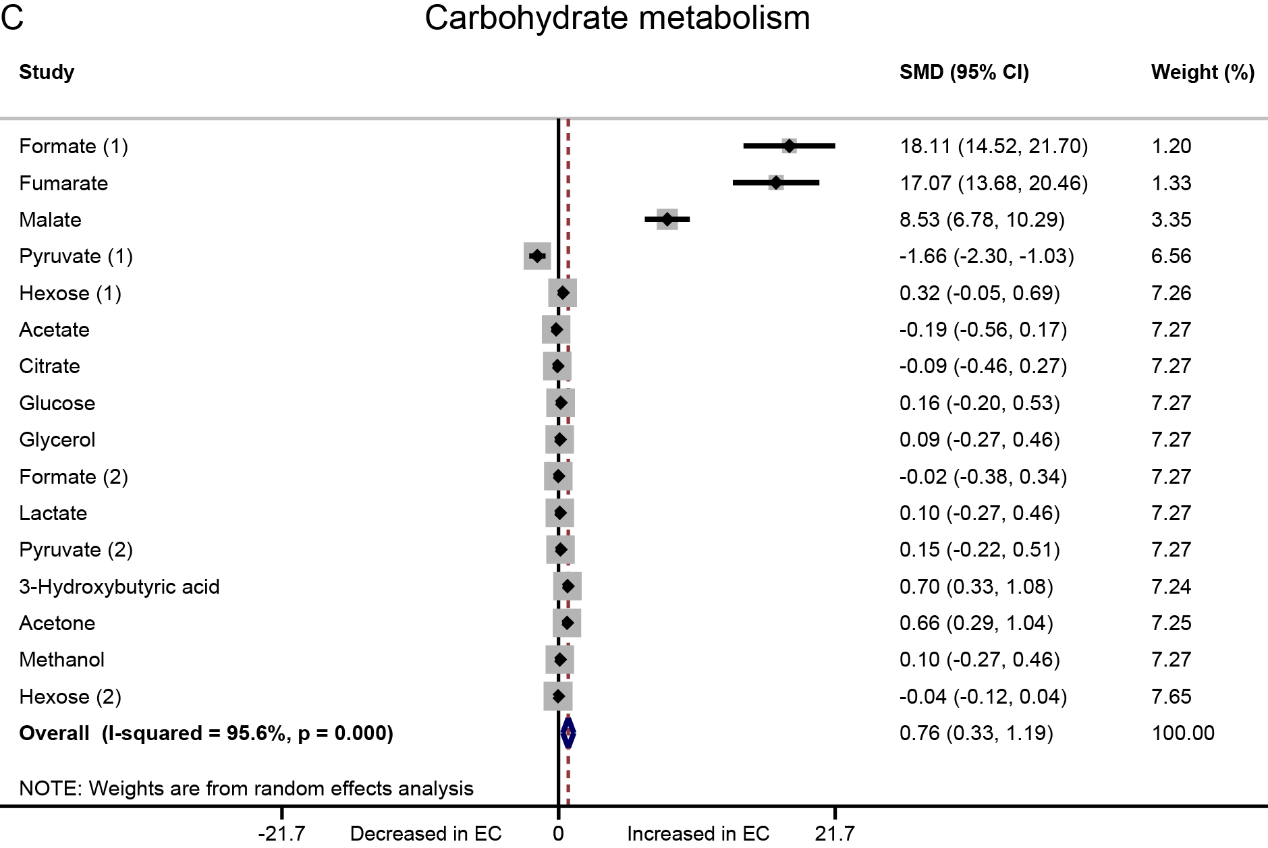
**


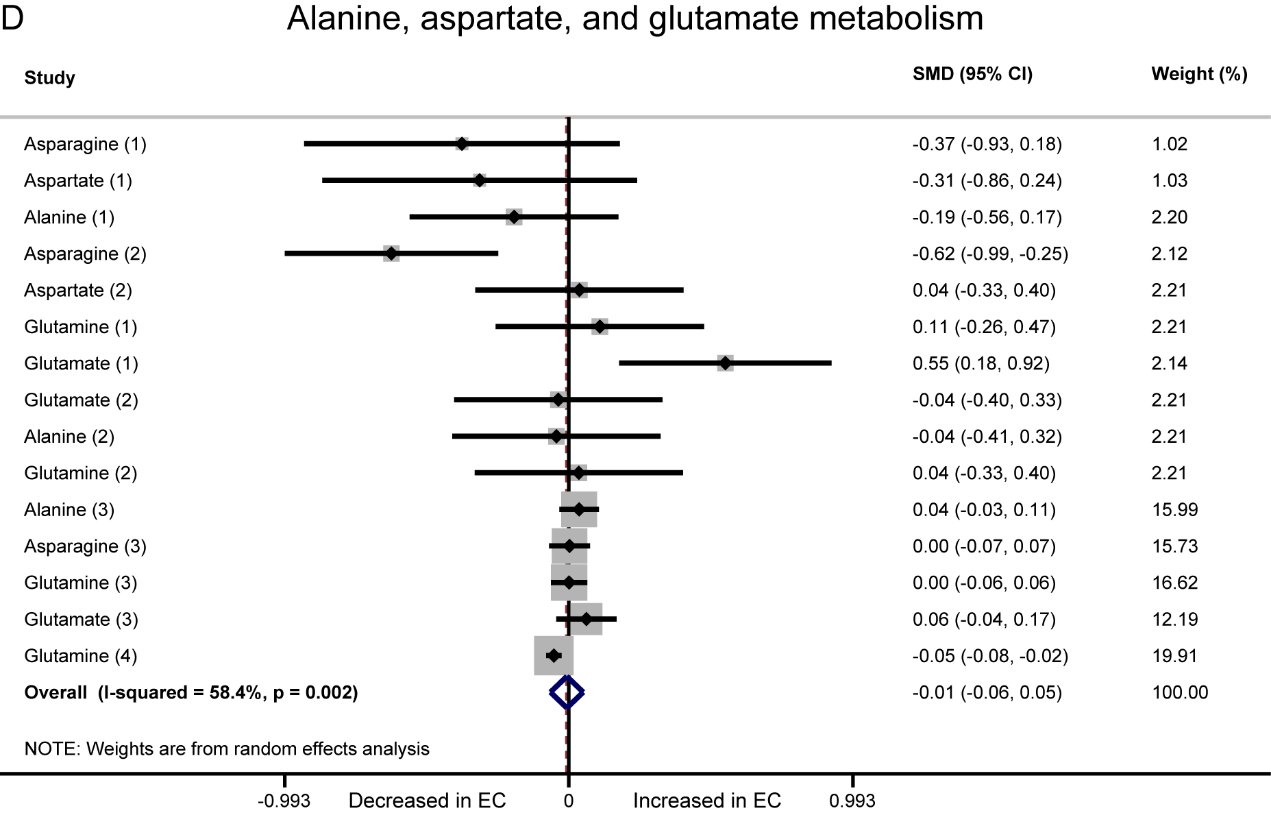


**
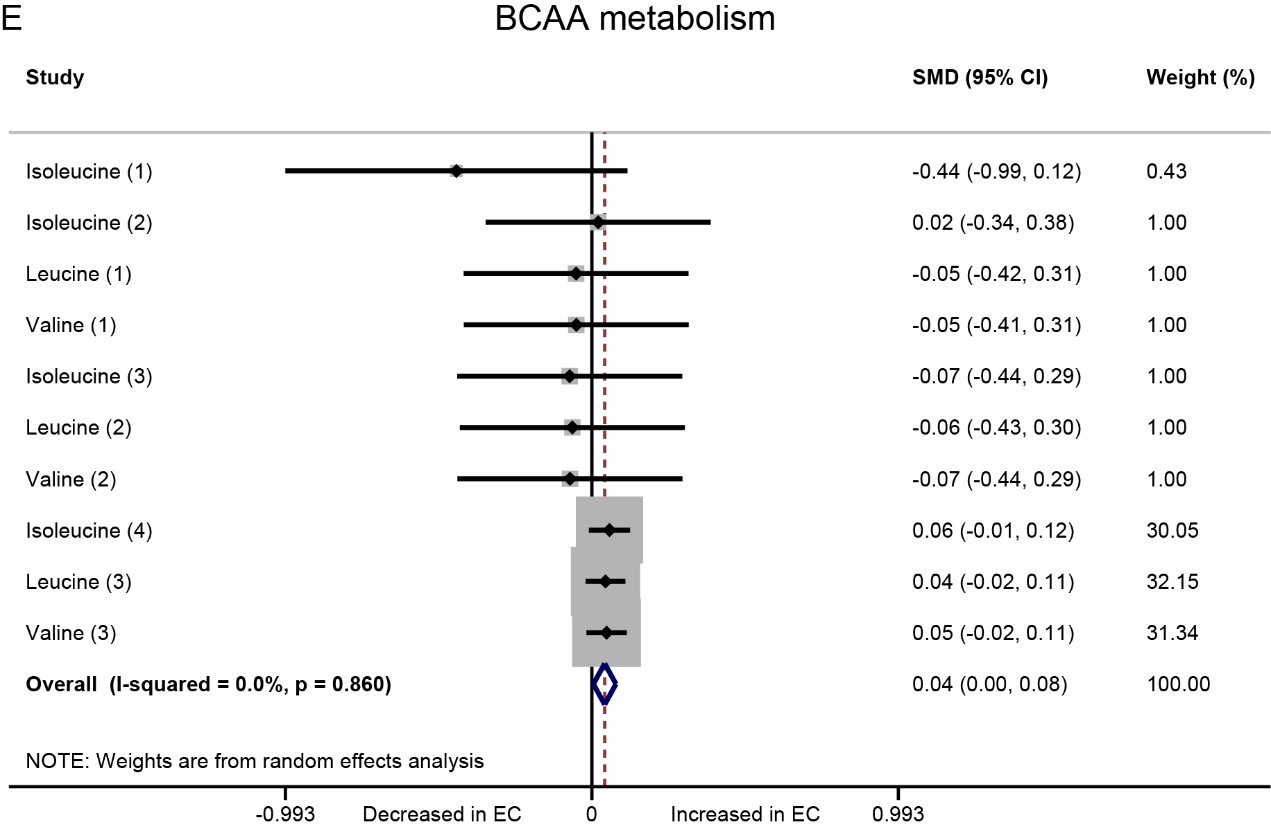
**

**
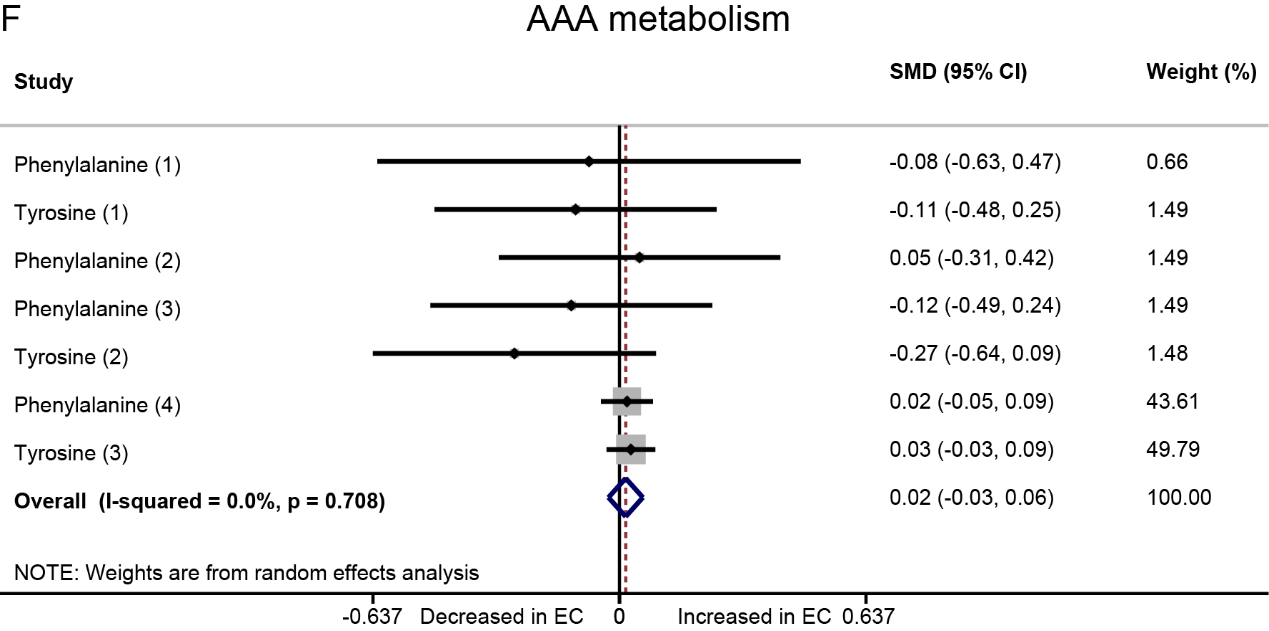
**


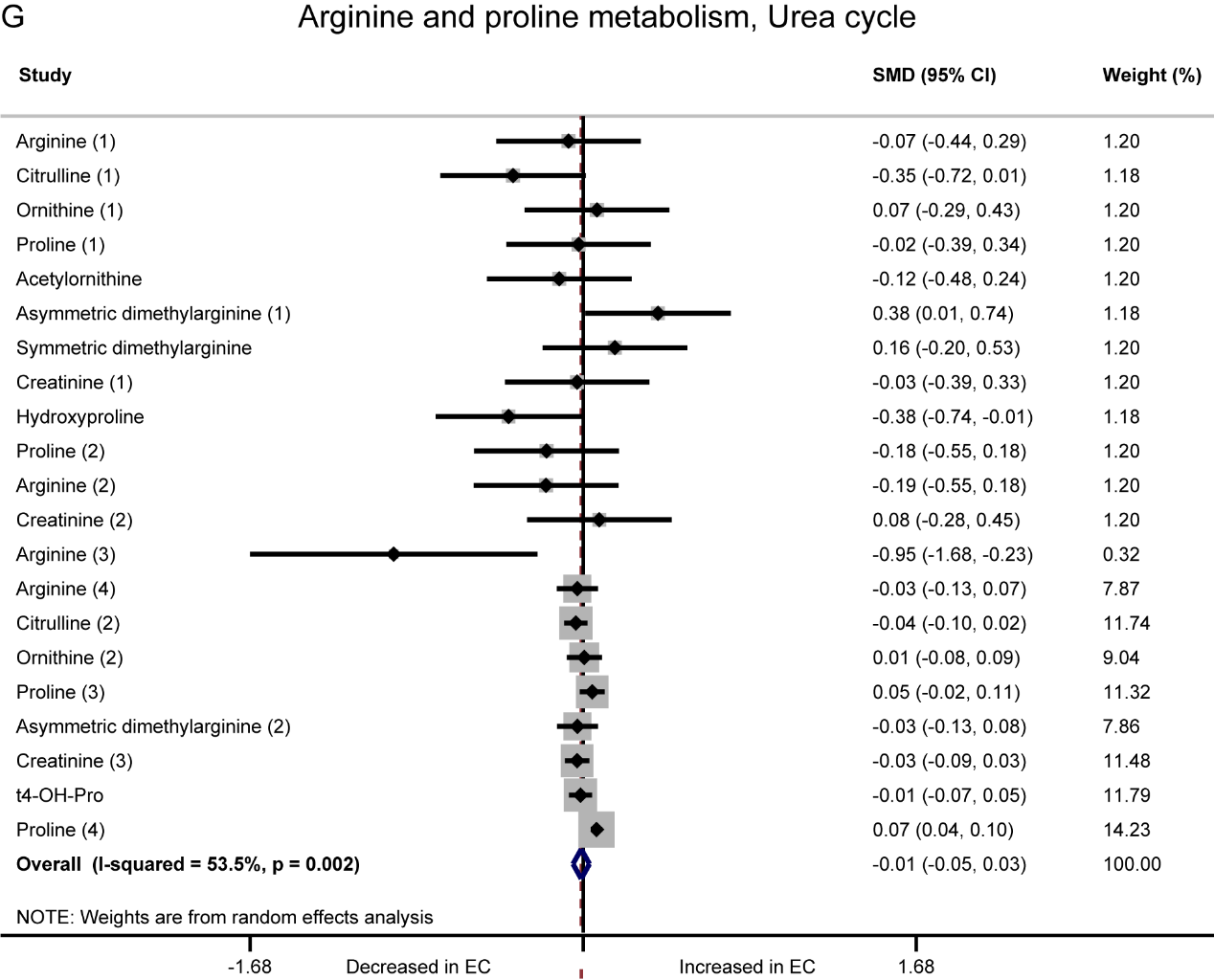


**
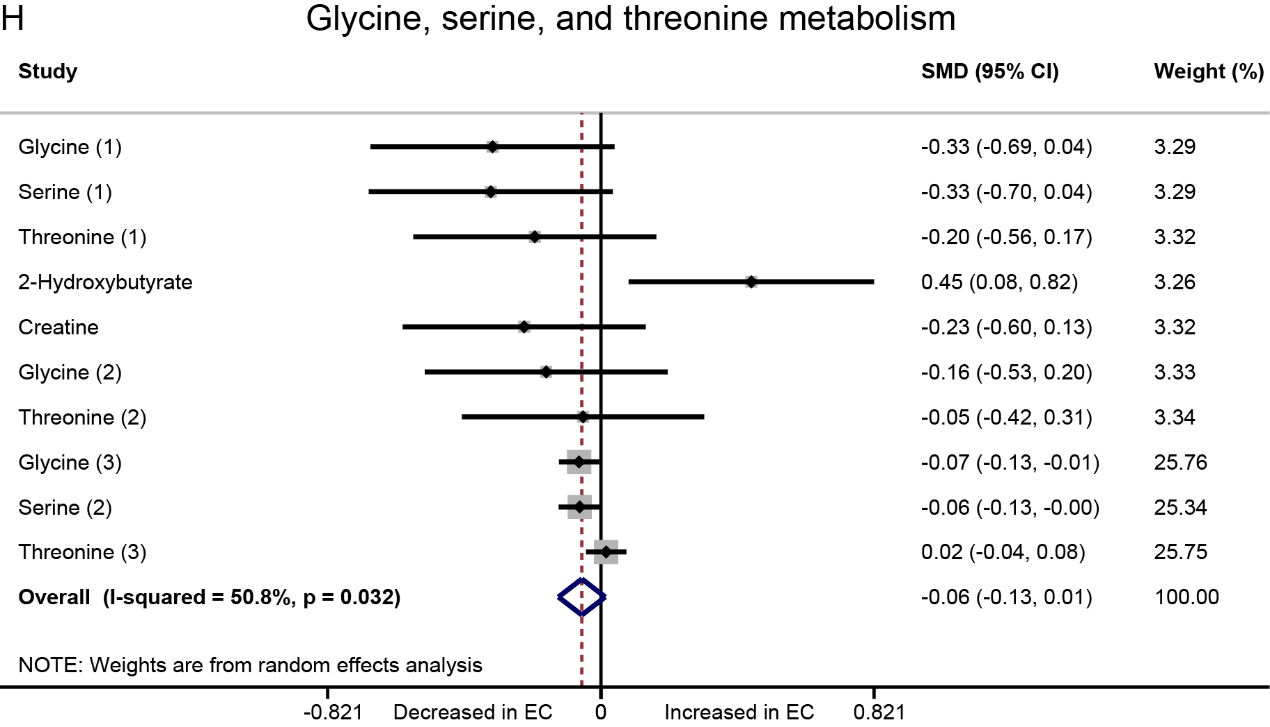
**

**
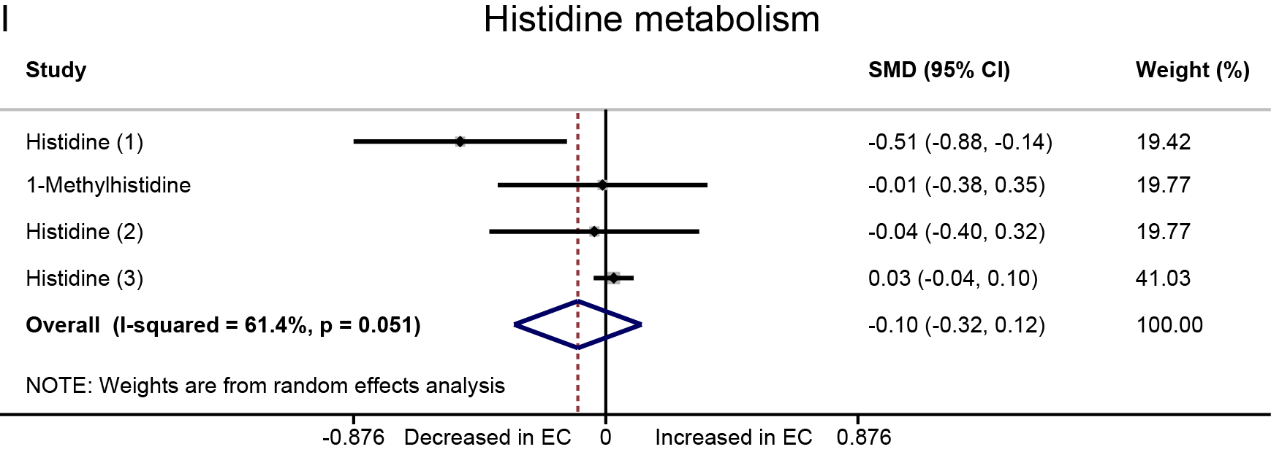
**

**
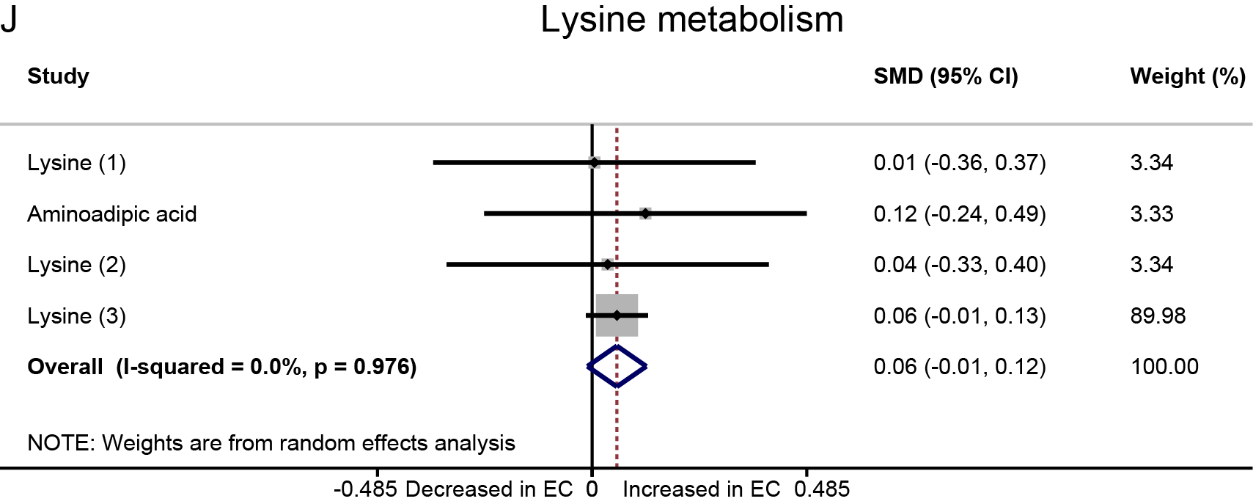
**

**
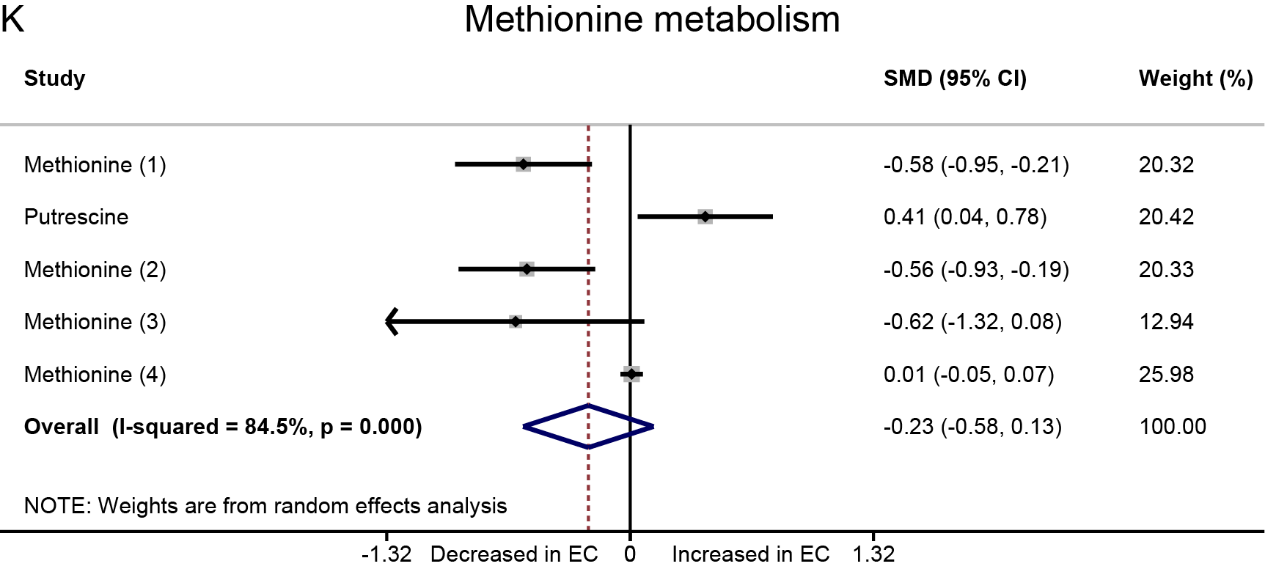
**

**
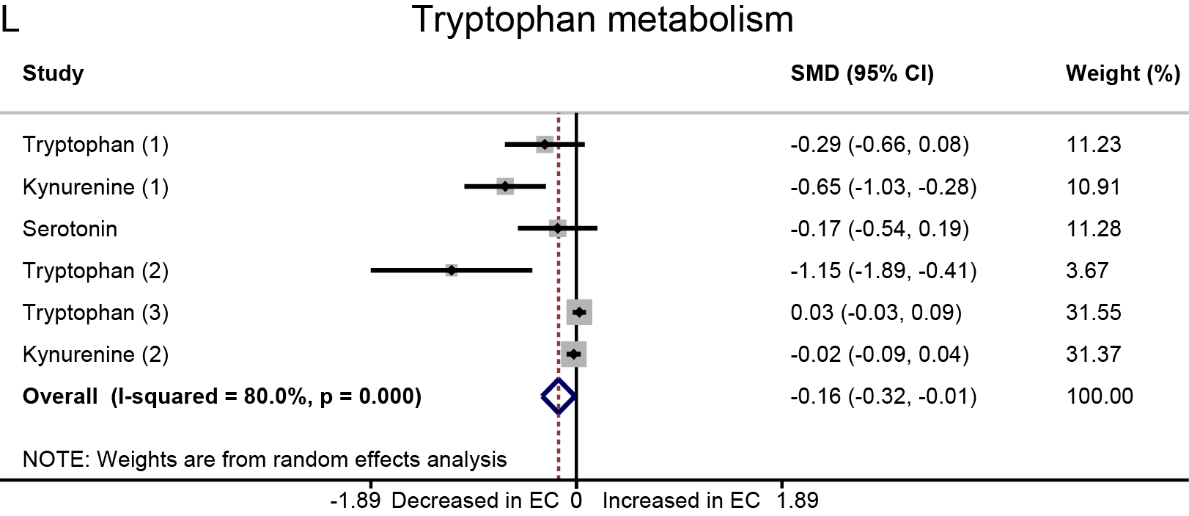
**

**
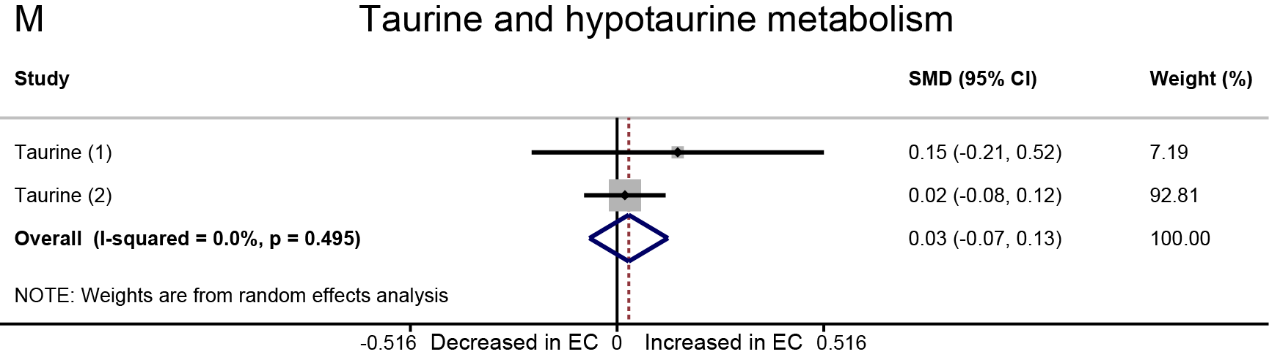
**


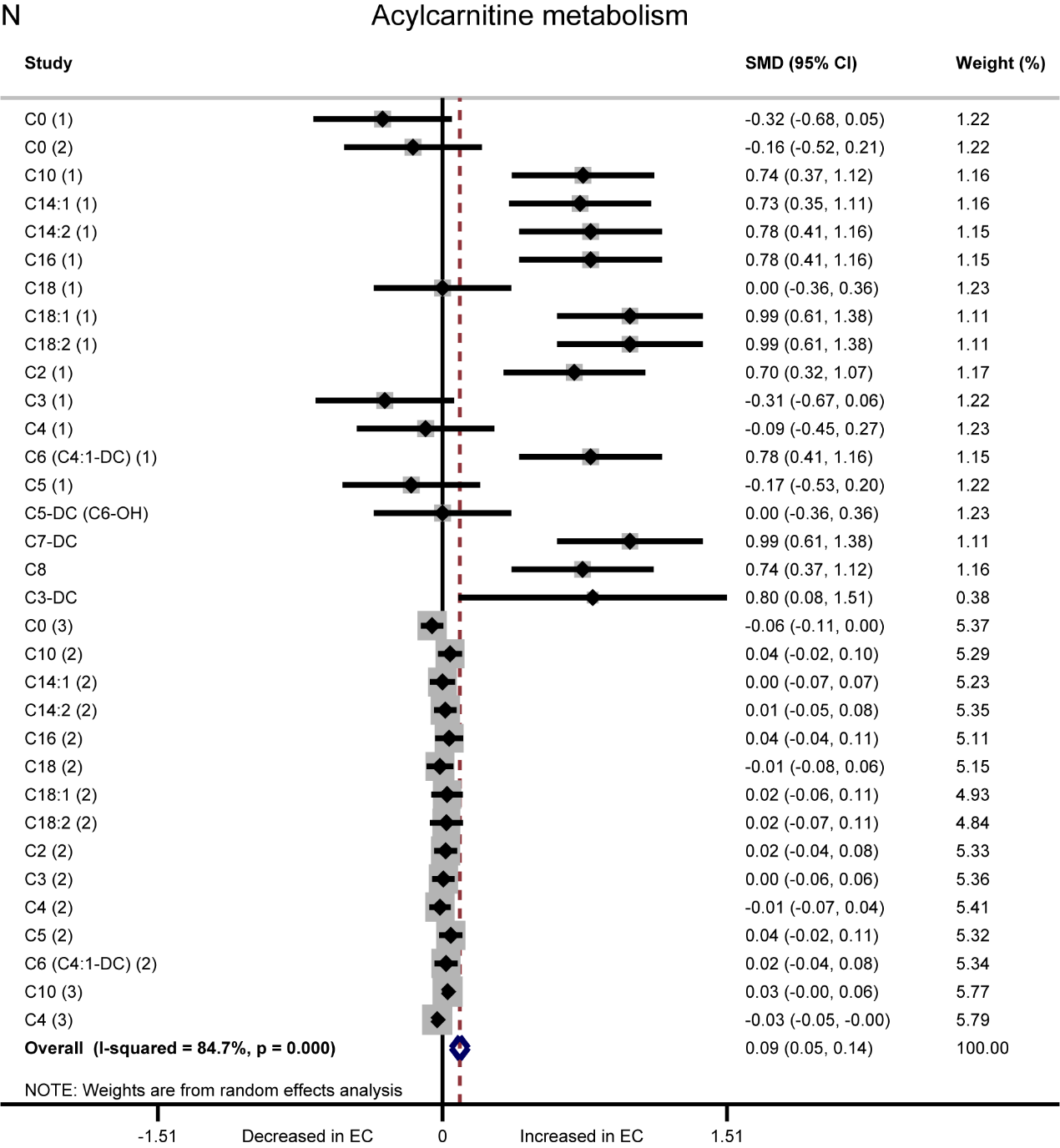


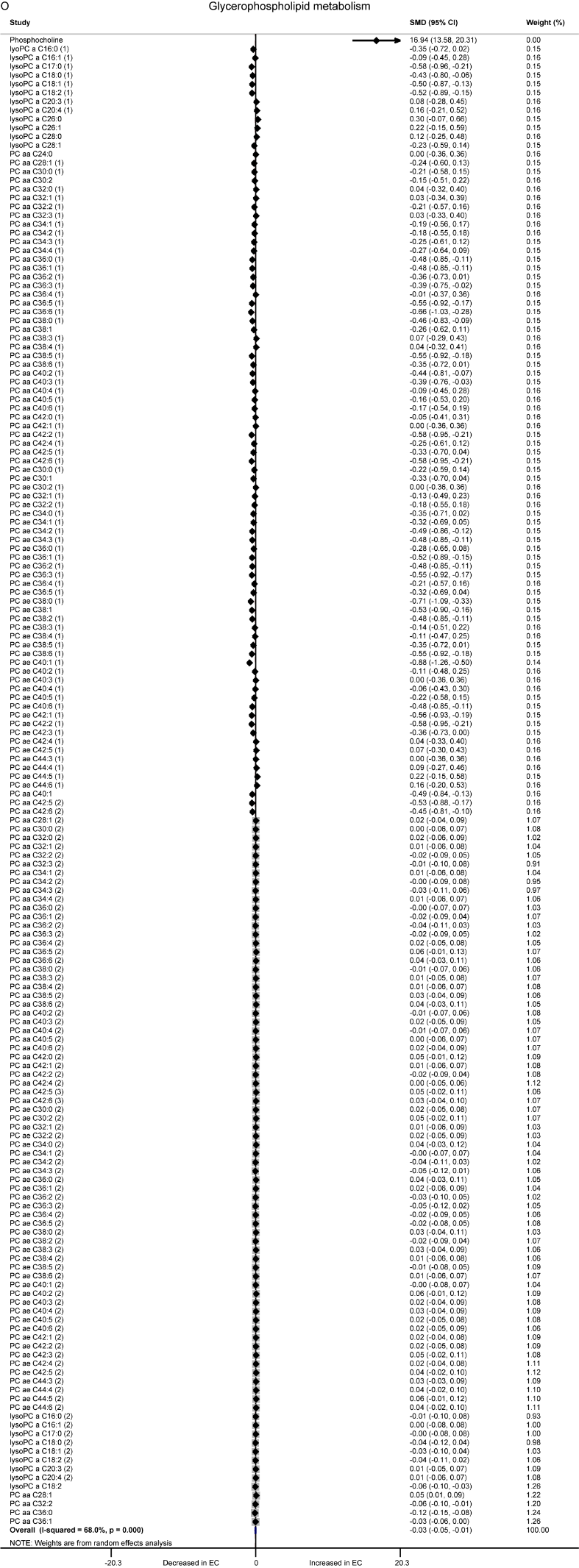


**
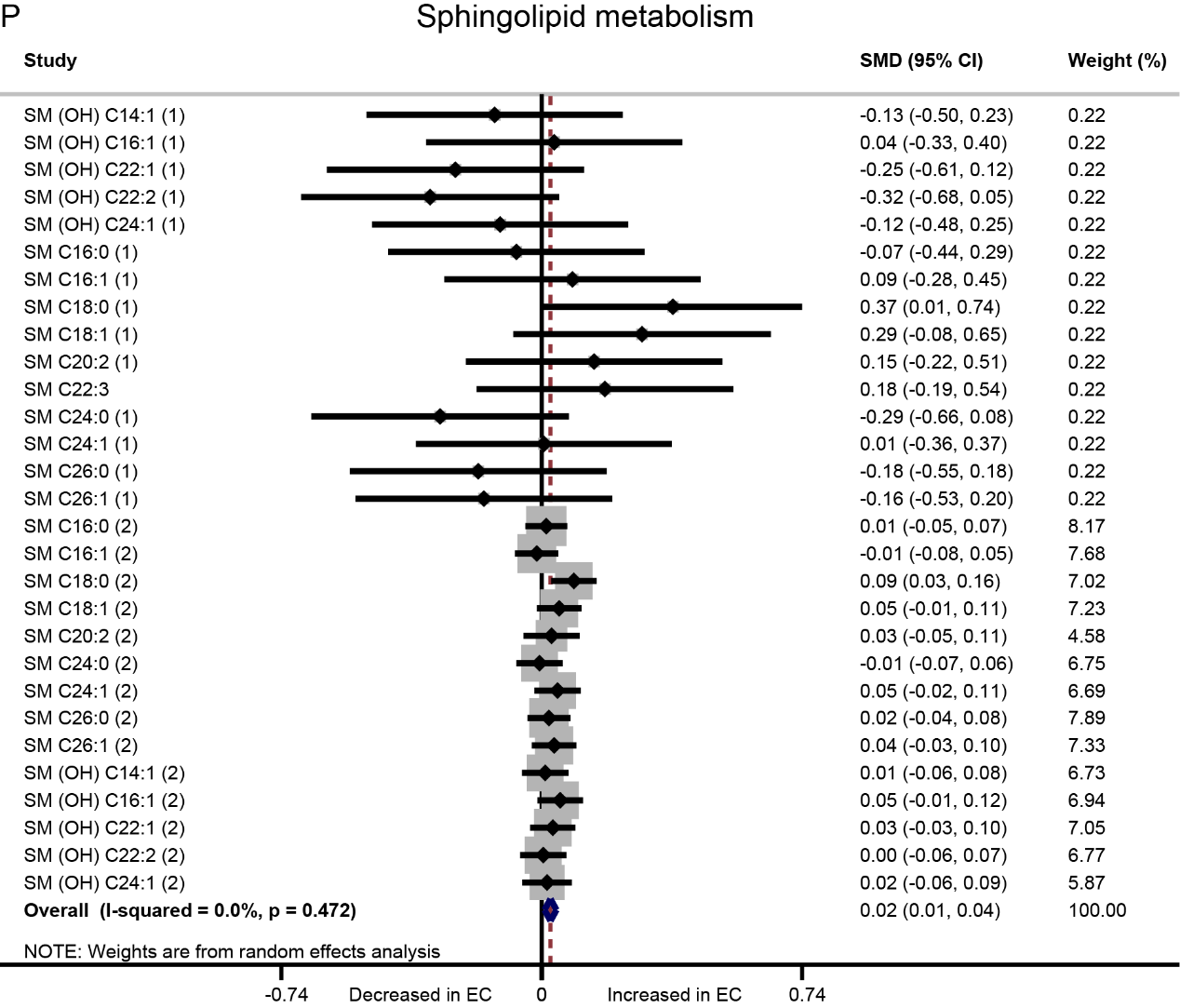
**

**Figure S3: Forest plots assessing changes in the levels of metabolic pathways associated with endometrial cancer. (A) Cholesterol hormone metabolism; (B) Choline metabolism; (C) Carbohydrate metabolism; (D) Alanine, aspartate, and glutamate metabolism; (E) BCAA metabolism; (F) AAA metabolism; (G) Arginine and proline metabolism, Urea cycle; (H) Glycine, serine, and threonine metabolism; (I) Histidine metabolism; (J) Lysine metabolism; (K) Methionine metabolism; (L) Tryptophan metabolism; (M) Taurine and hypotaurine metabolism; (N) Acylcarnitine metabolism; (O) Glycerophospholipid metabolism; (P) Sphingolipid metabolism.** AAA, aromatic amino acid; BCAA, branched-chain amino acid; C0, carnitine; C10, decanoylcarnitine; C14:1, tetradecenoylcarnitine; C14:2, tetradecadienoylcarnitine; C16, hexadecenoylcarnitine; C18, 3-hydroxylhexadecanoylcarnitine; C18:1, octadecenoylcarnitine; C18:2, octadecadienoylcarnitine; C2, acetylcarnitine; C3, propionylcarnitine; C3-DC, malonylcarnitine; C4, malonylcarnitine; C5, methylmalonylcarnitine; C5-DC, 3-hydroxyl-isovalerylcarnitine; C6, glutarylcarnitine; C7-DC, pimelylcarnitine; C8, 3-methylglutarylcarnitine; LysoPC, lysophosphatidylcholine; PC, Phosphatidylcholine; SM, sphingomyelin; SMD, standardized mean difference.


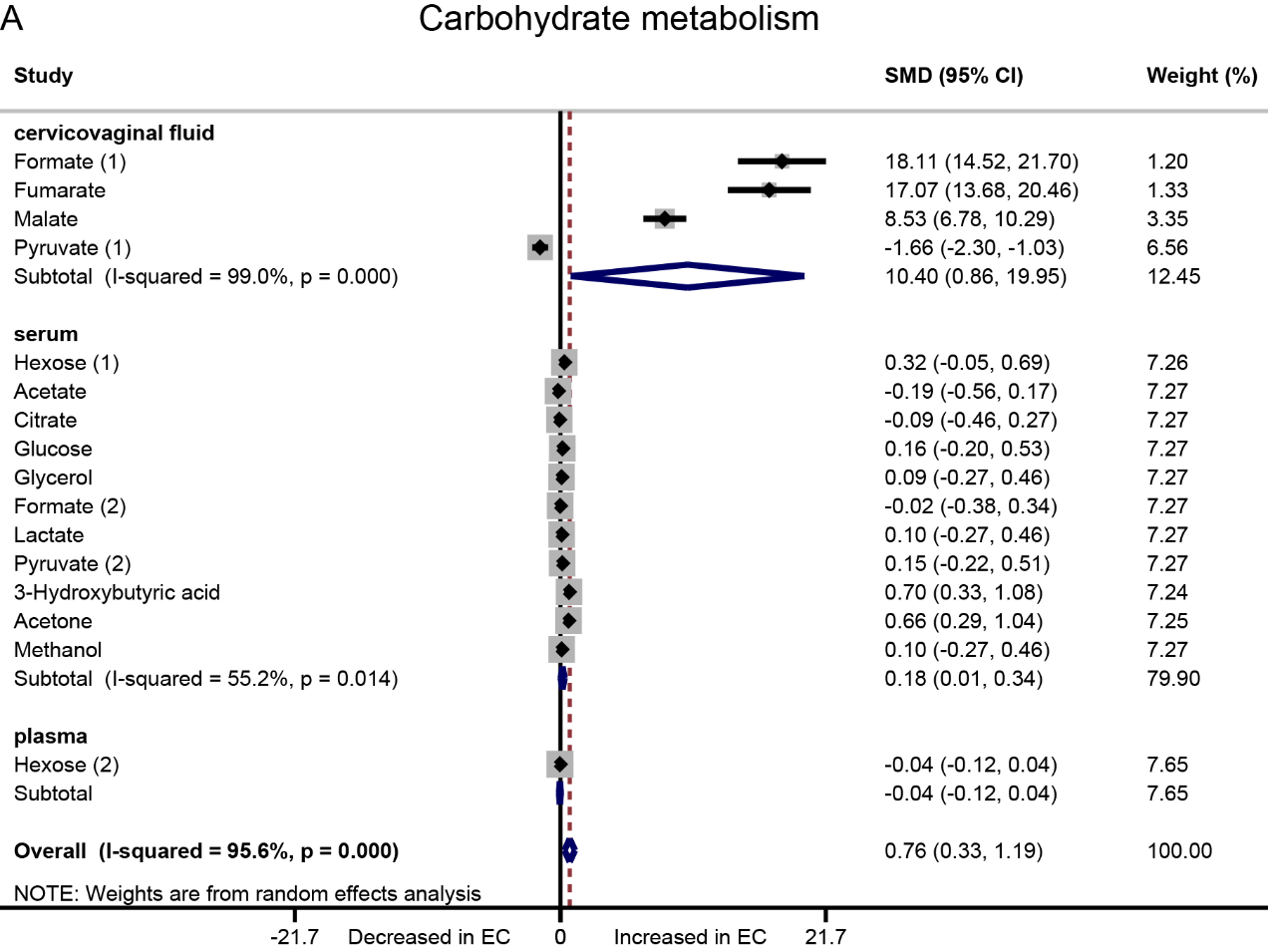


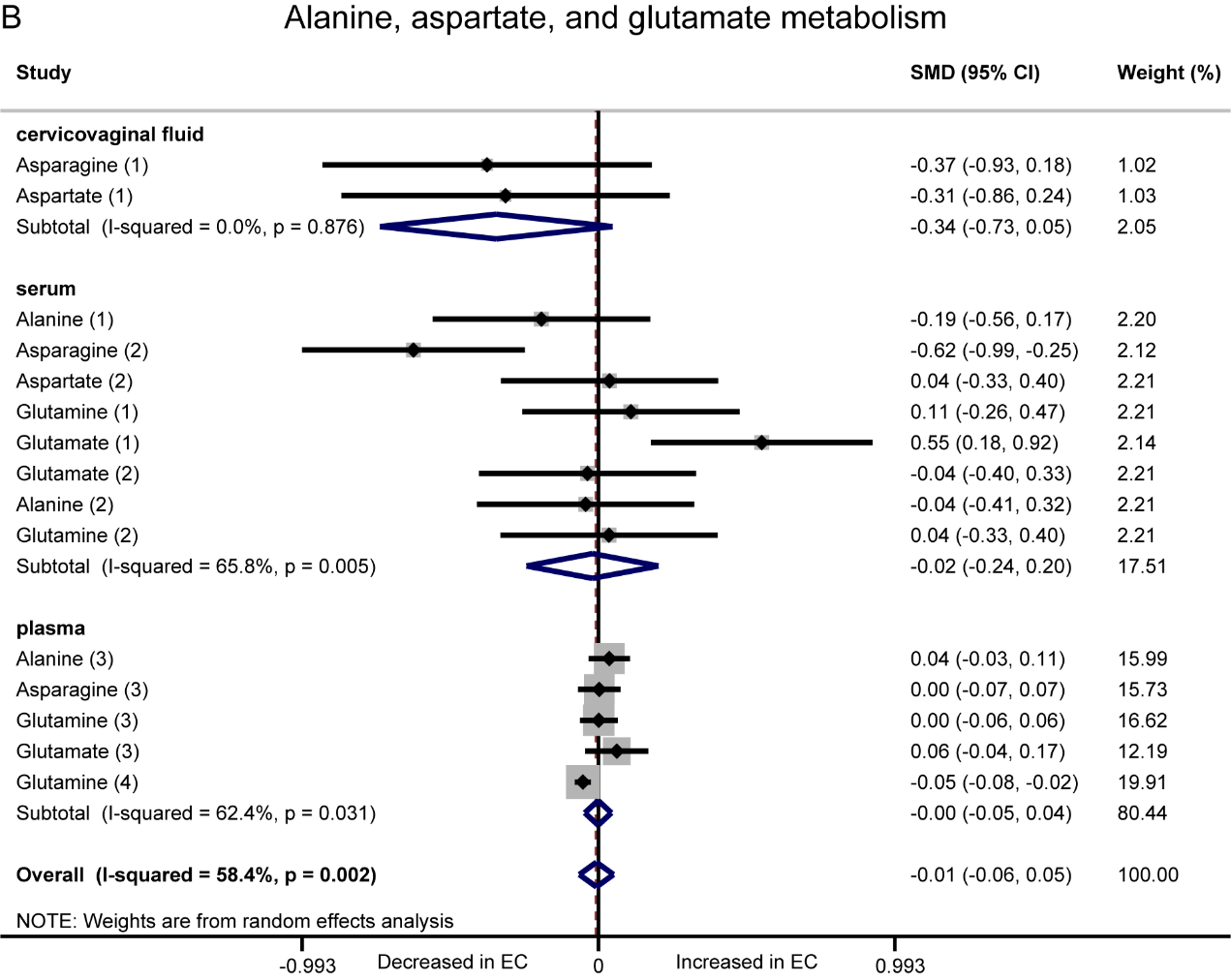


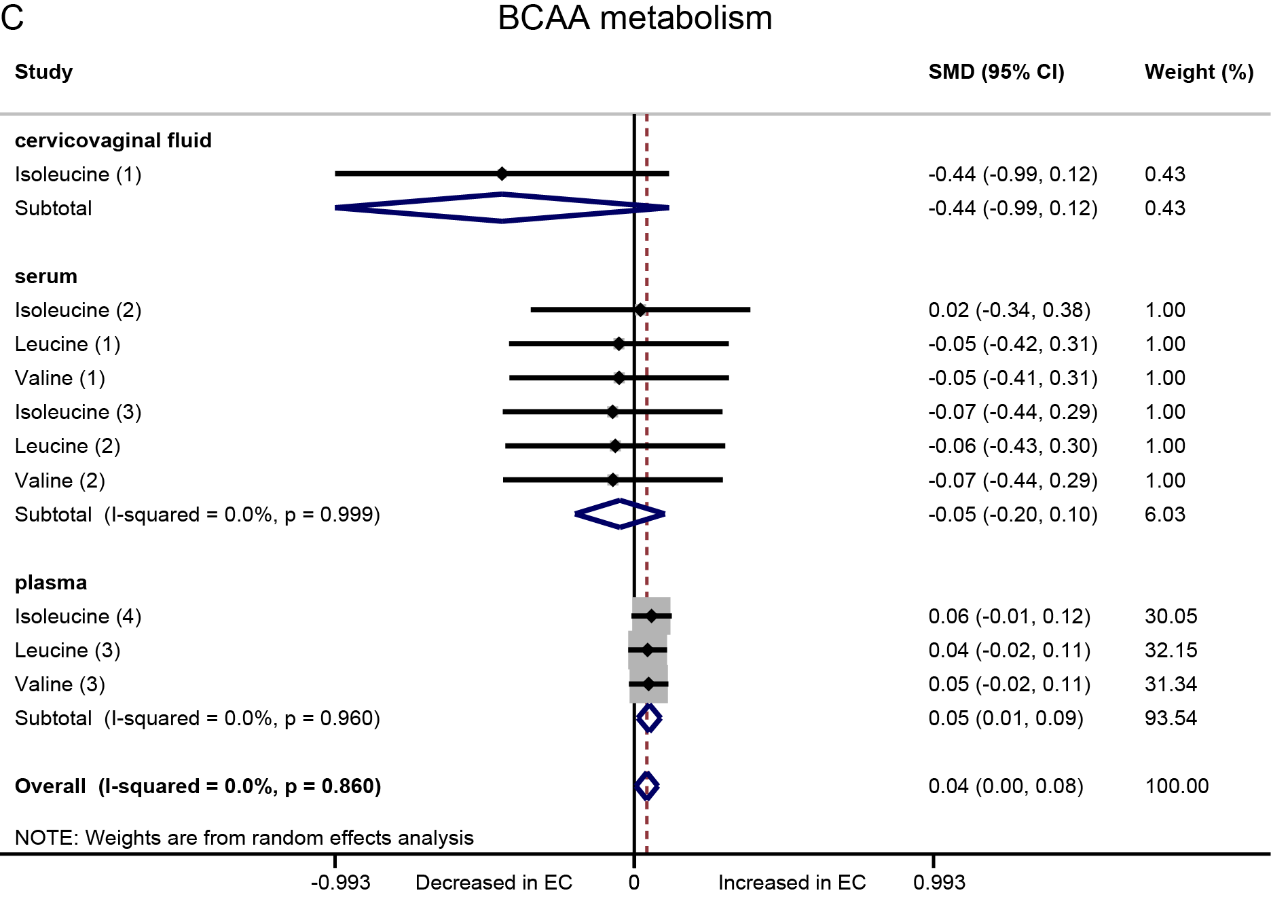


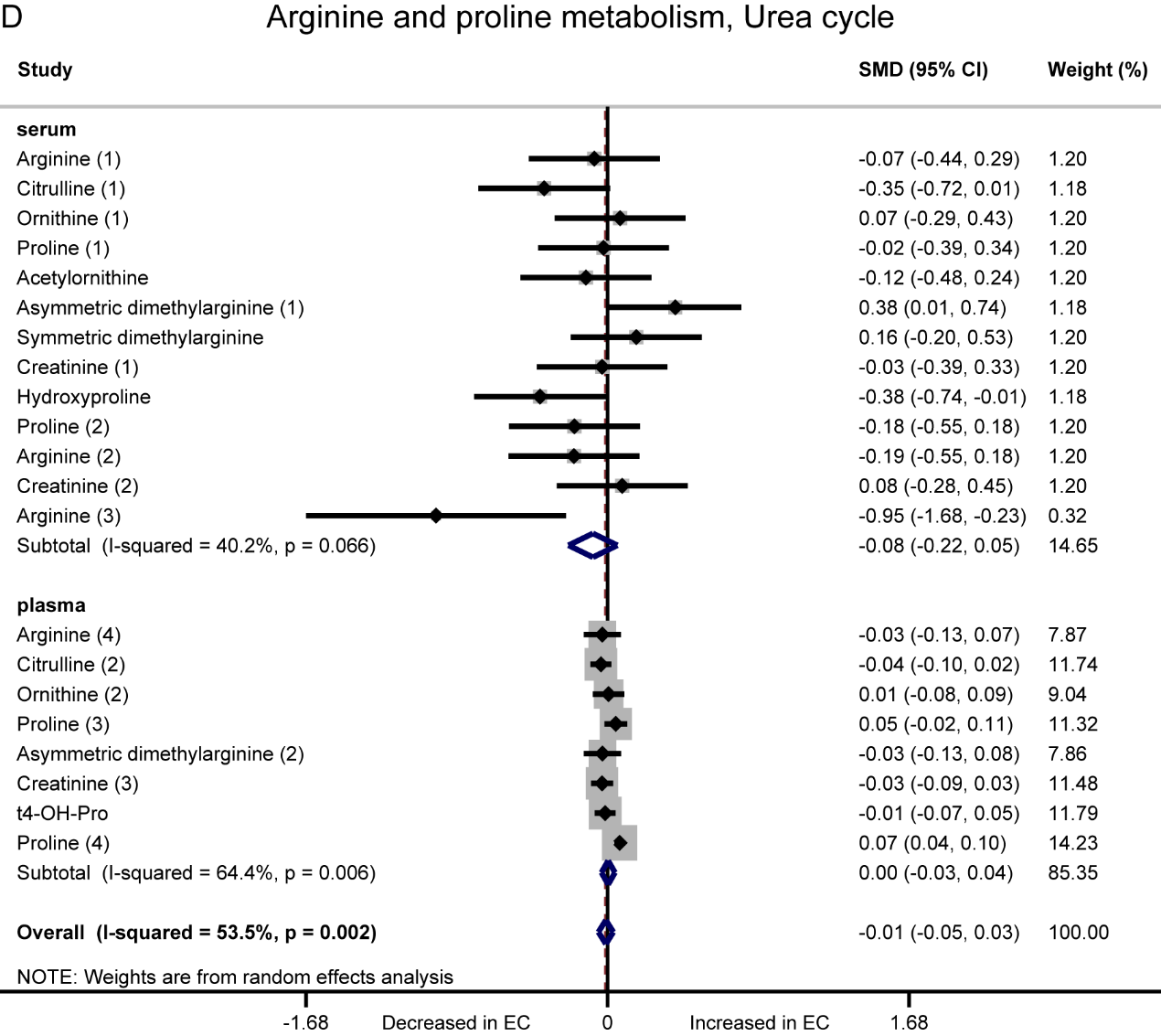


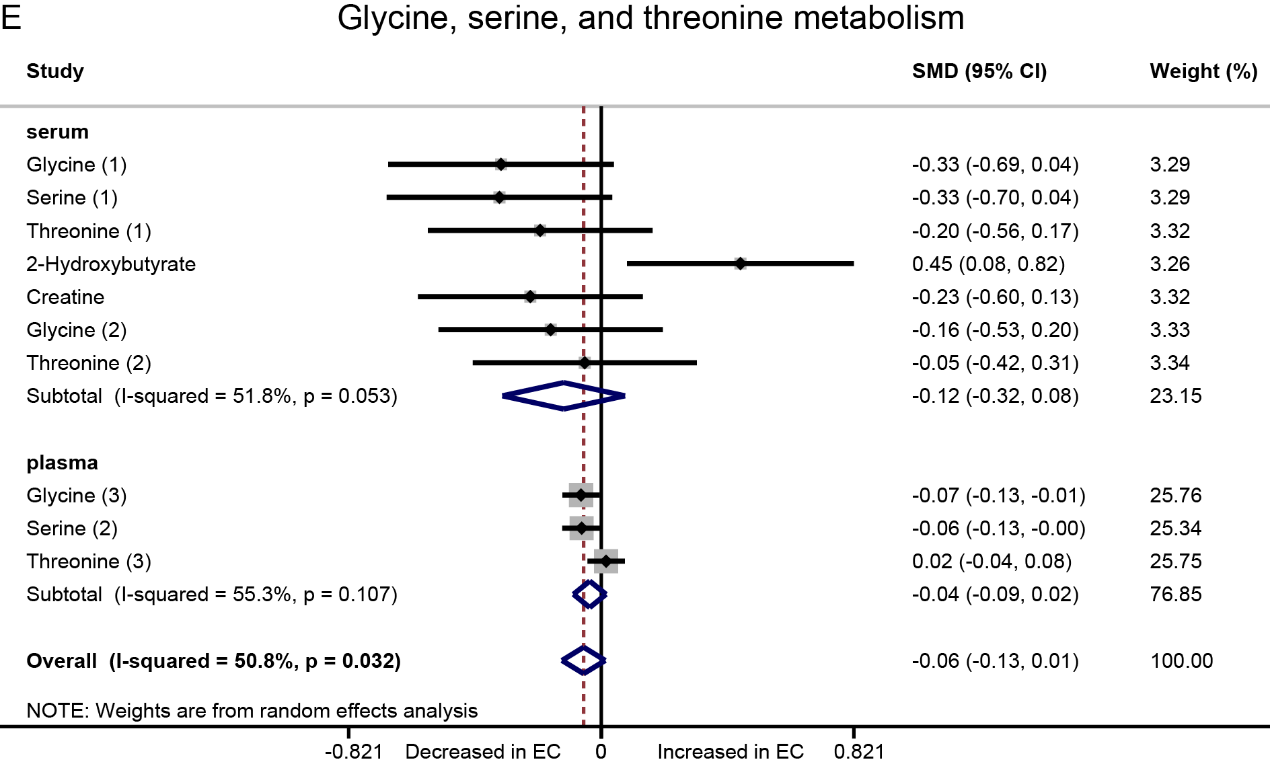


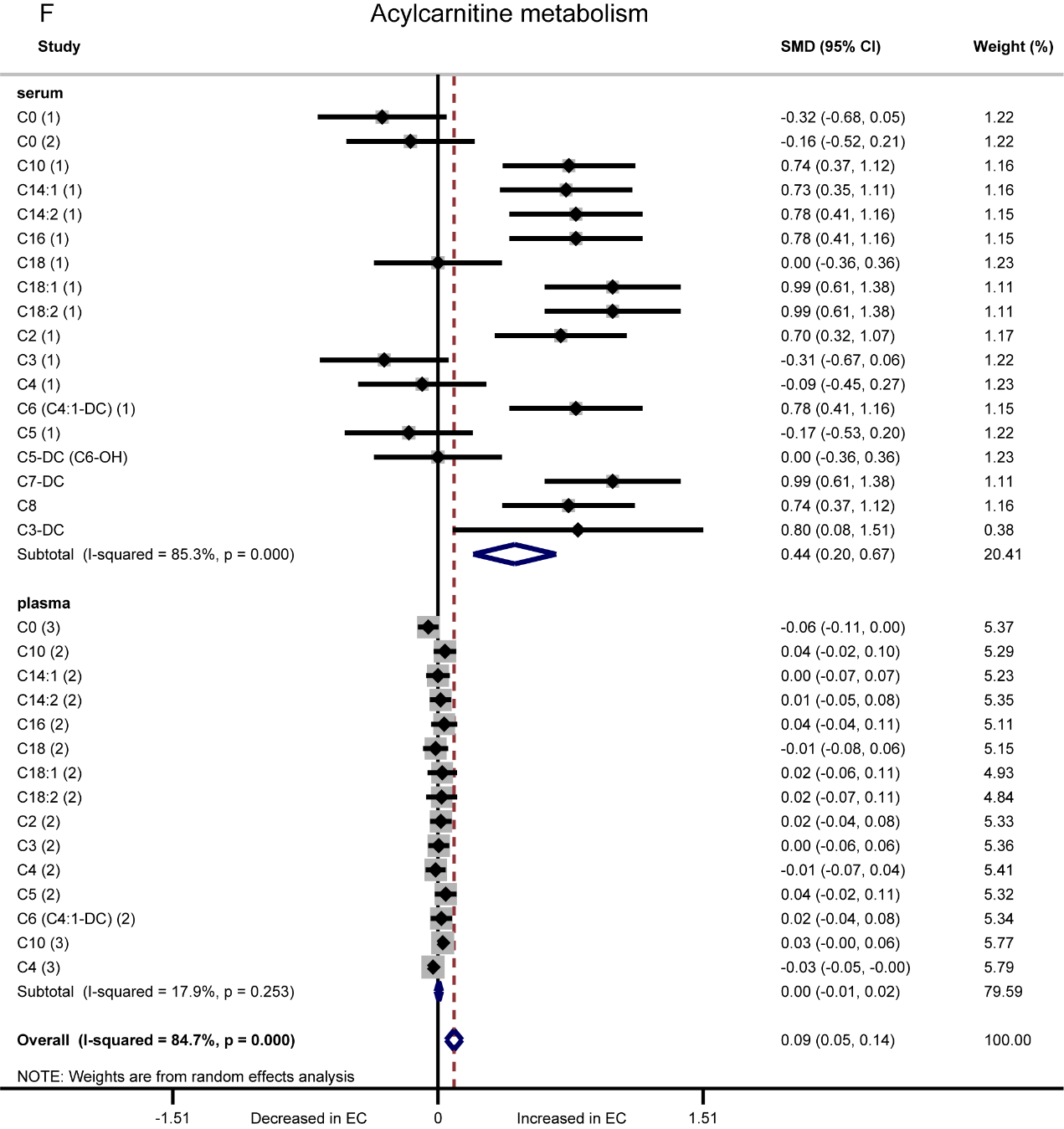


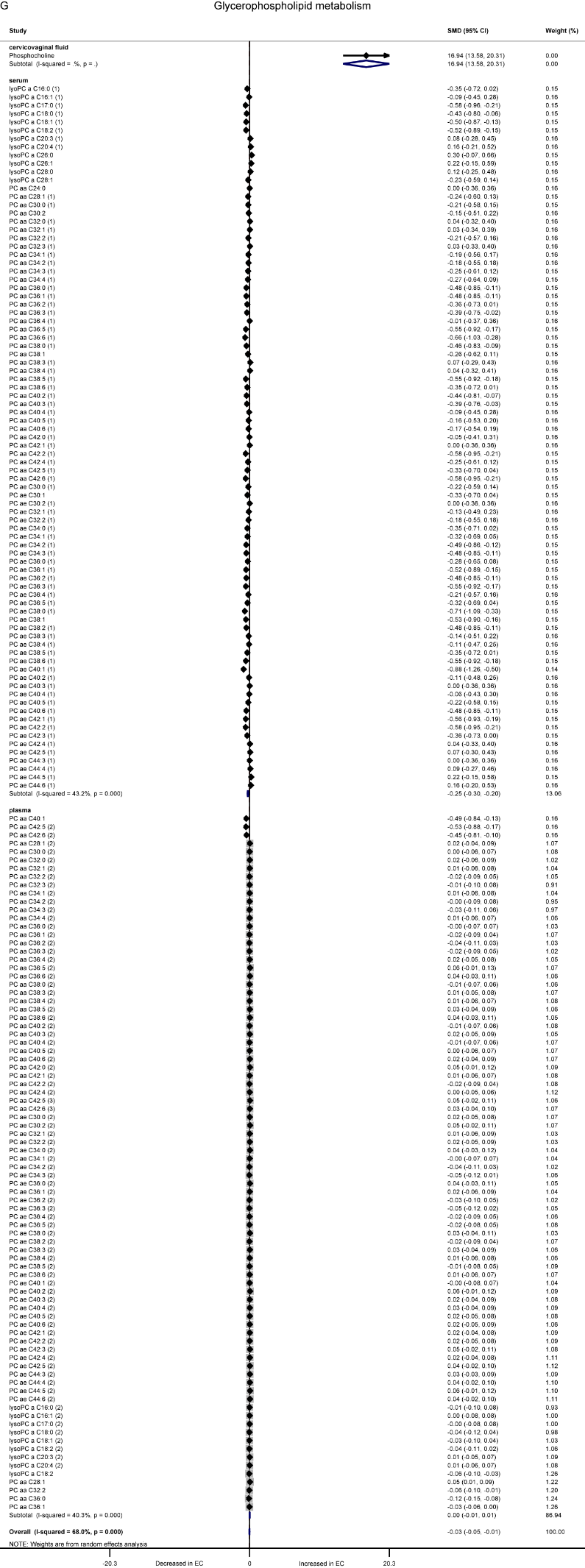


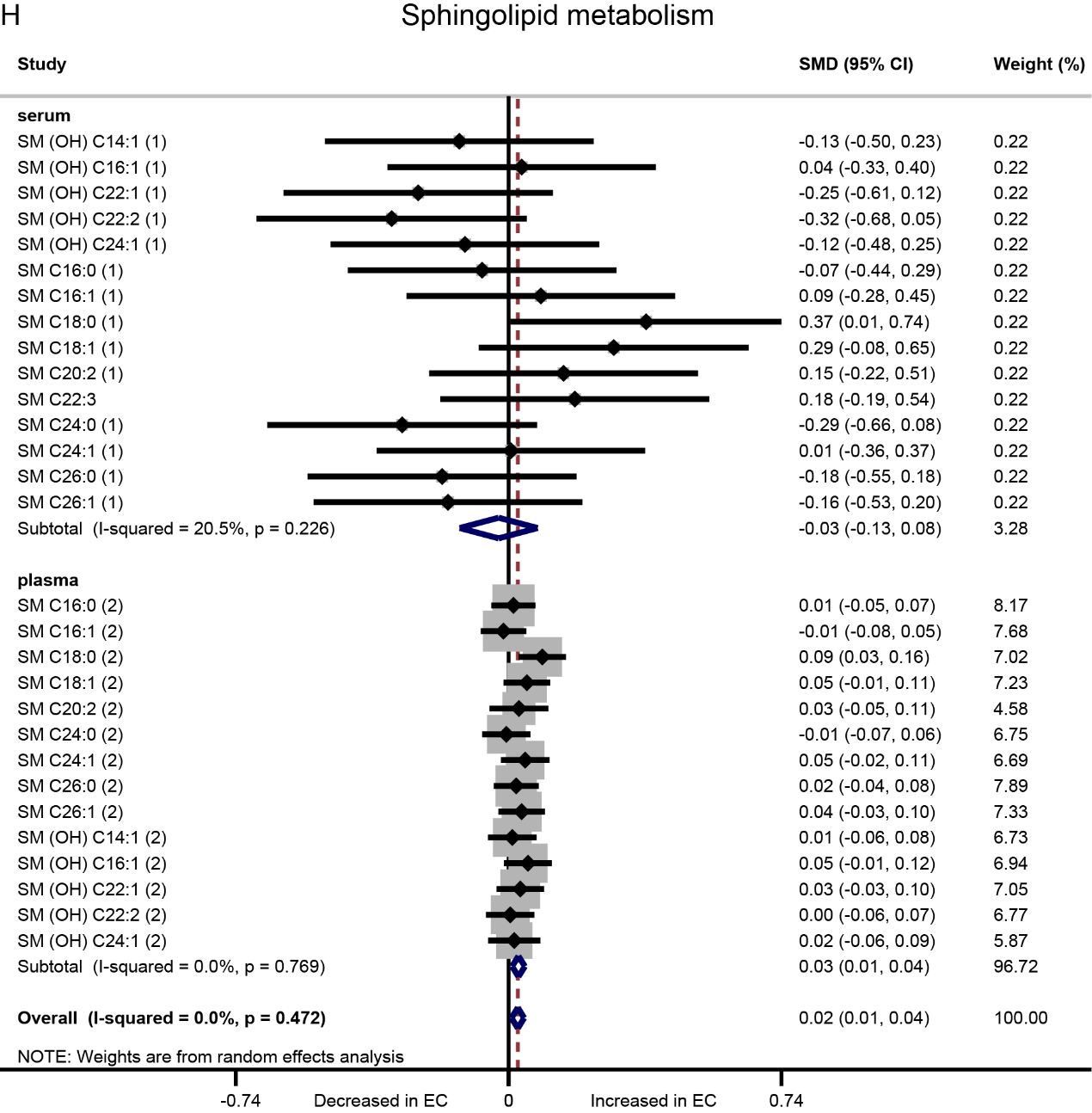


**Figure S4: Forest plots of biological sample type subgroup analysis of endometrial cancer-associated metabolic pathways. (A) Carbohydrate metabolism; (B) Alanine, aspartate, and glutamate metabolism; (C) BCAA metabolism; (D) Arginine and proline metabolism, Urea cycle; (E) Glycine, serine, and threonine metabolism; (F) Acylcarnitine metabolism; (G) Glycerophospholipid metabolism; (H) Sphingolipid metabolism.** BCAA, branched-chain amino acid; C0, carnitine; C10, decanoylcarnitine; C14:1, tetradecenoylcarnitine; C14:2, tetradecadienoylcarnitine; C16, hexadecenoylcarnitine; C18, 3-hydroxylhexadecanoylcarnitine; C18:1, octadecenoylcarnitine; C18:2, octadecadienoylcarnitine; C2, acetylcarnitine; C3, propionylcarnitine; C3-DC, malonylcarnitine; C4, malonylcarnitine; C5, methylmalonylcarnitine; C5-DC, 3-hydroxyl-isovalerylcarnitine; C6, glutarylcarnitine; C7-DC, pimelylcarnitine; C8, 3-methylglutarylcarnitine; LysoPC, lysophosphatidylcholine; PC, Phosphatidylcholine; SM, sphingomyelin; SMD, standardized mean difference.


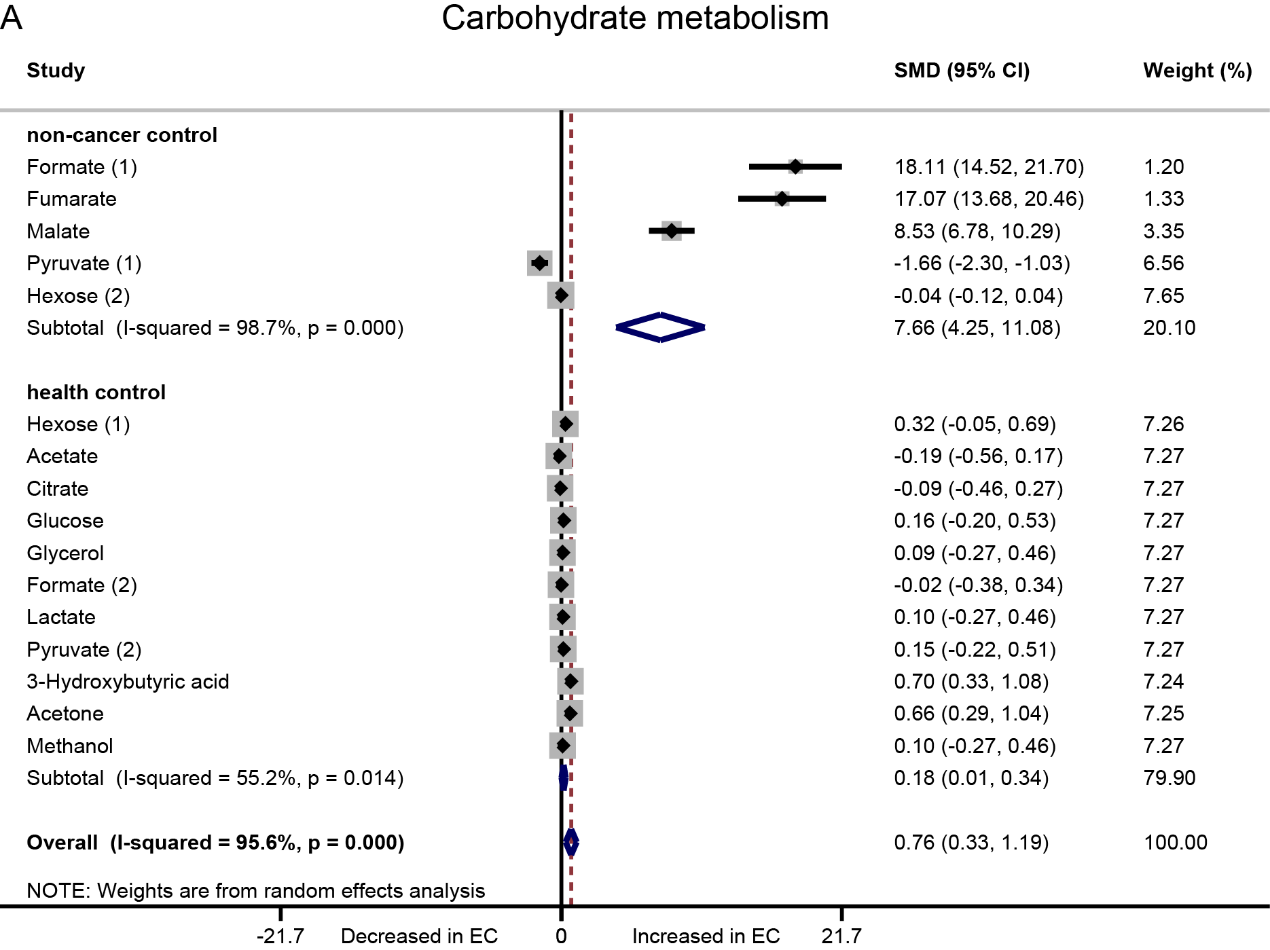


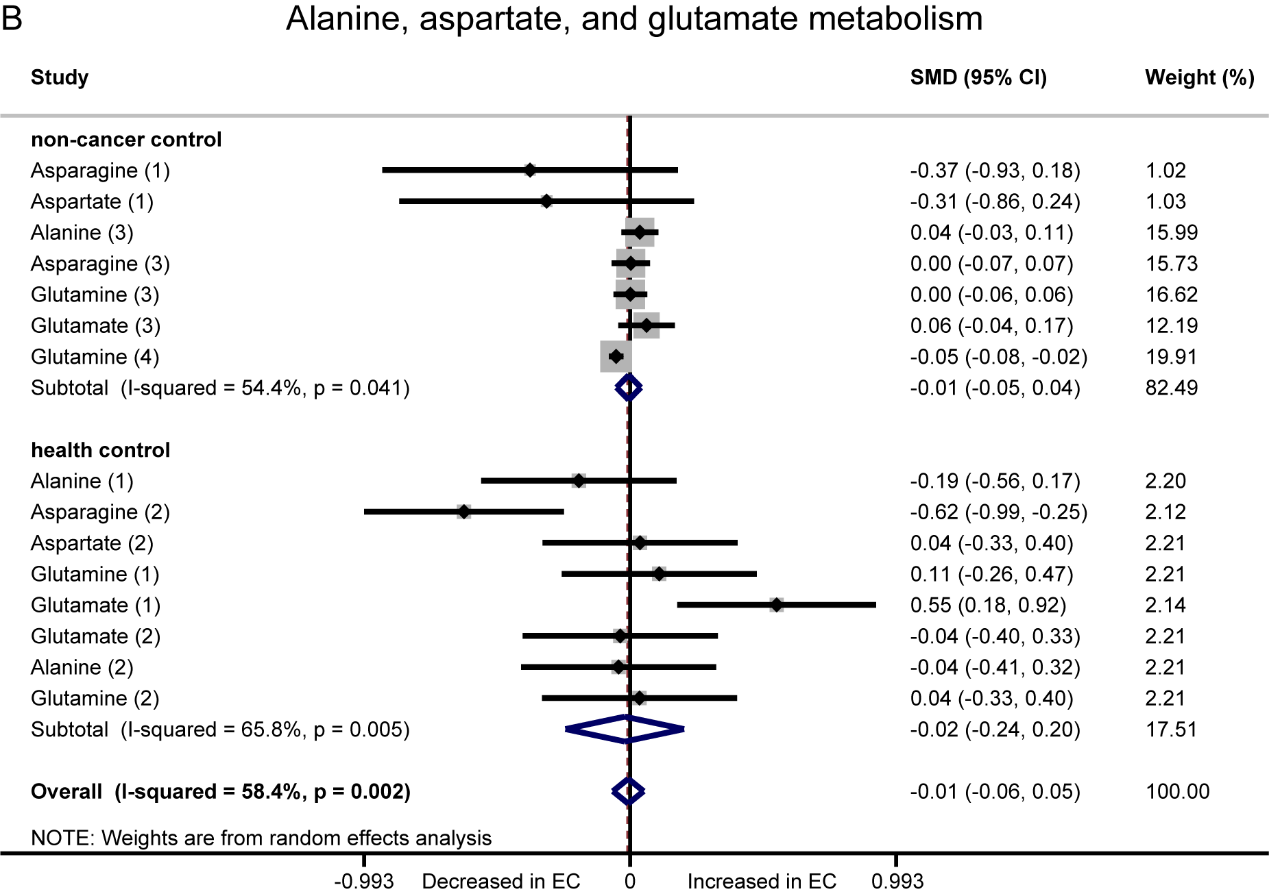


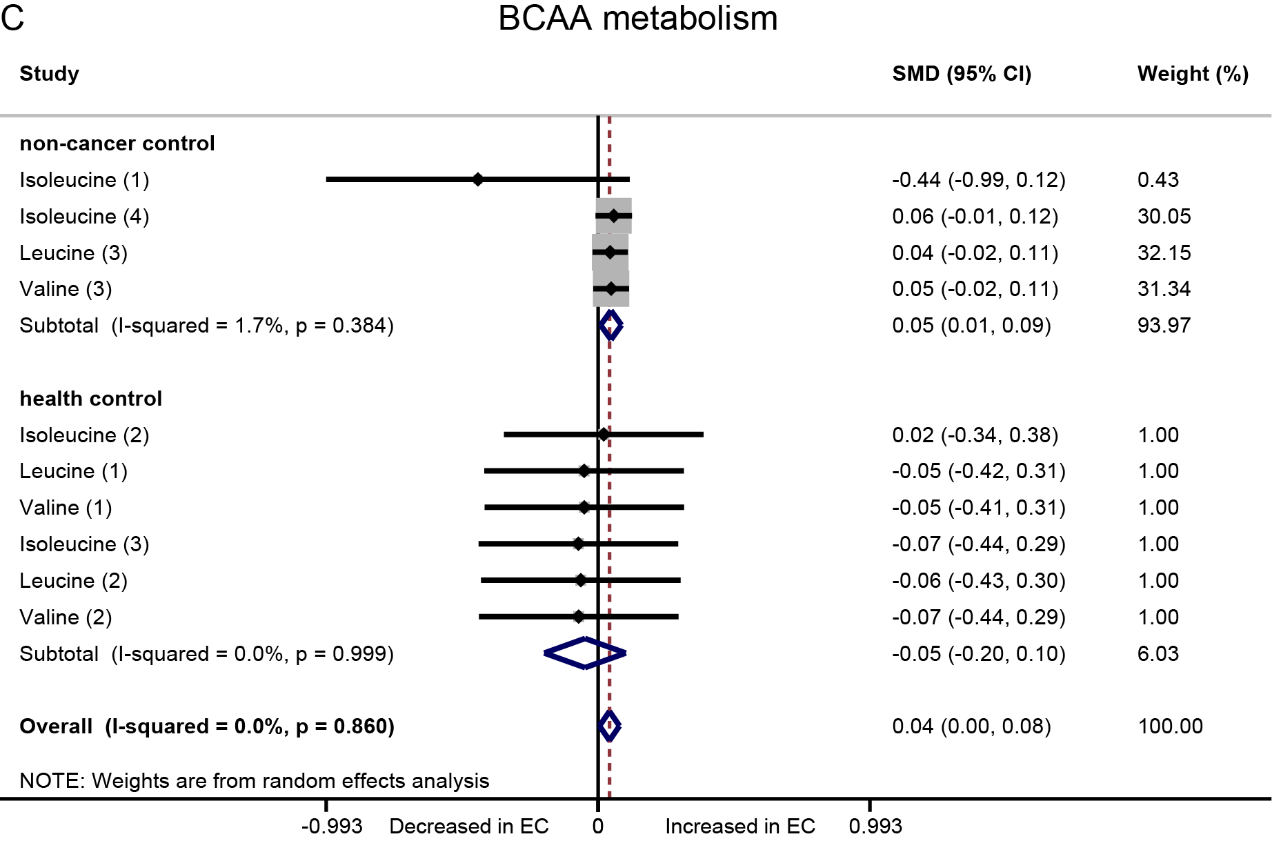


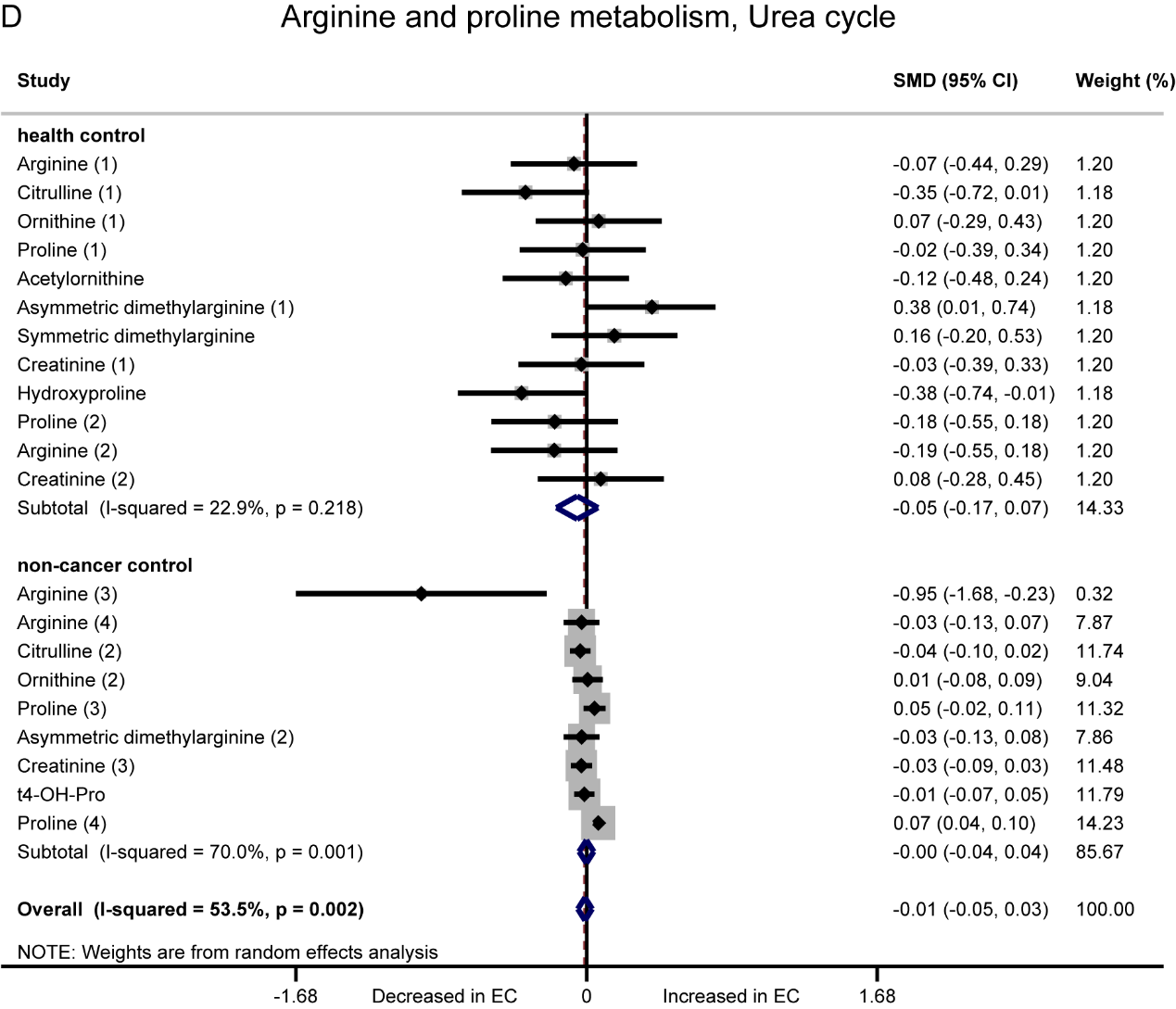


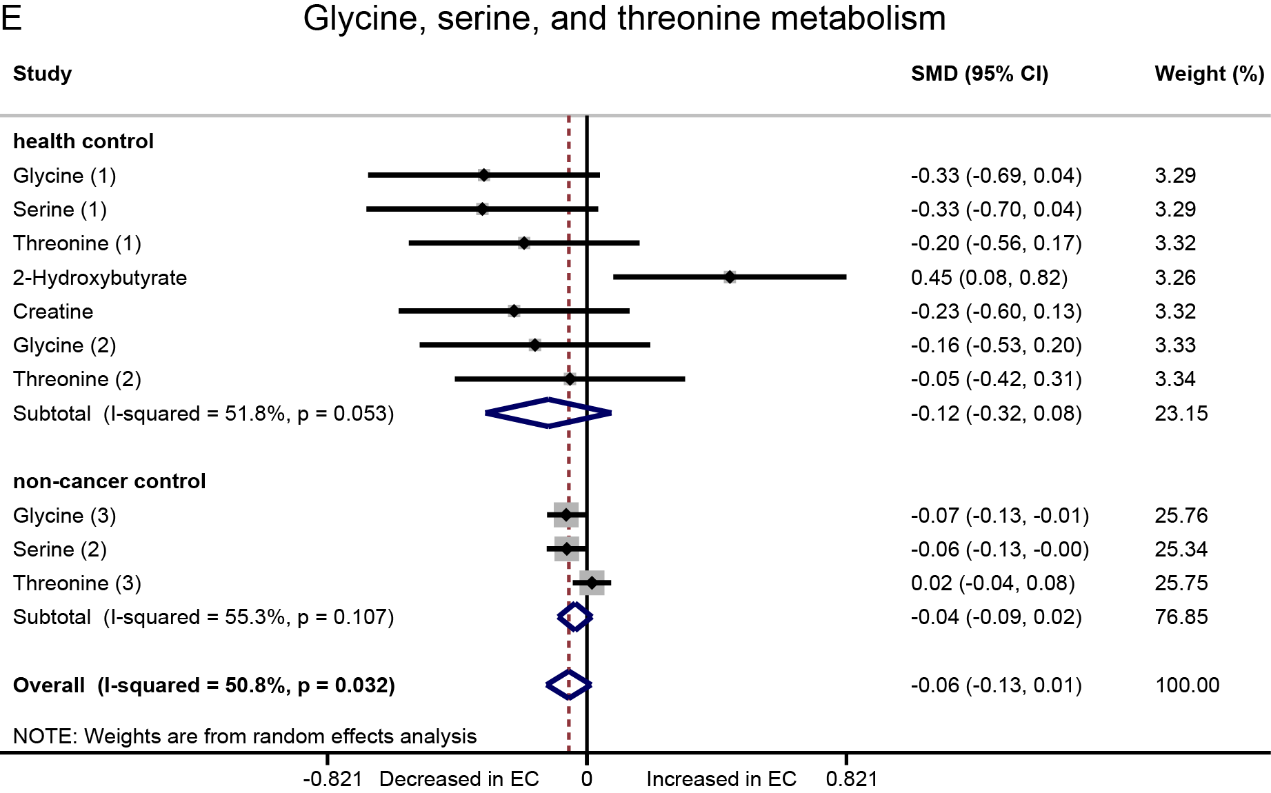


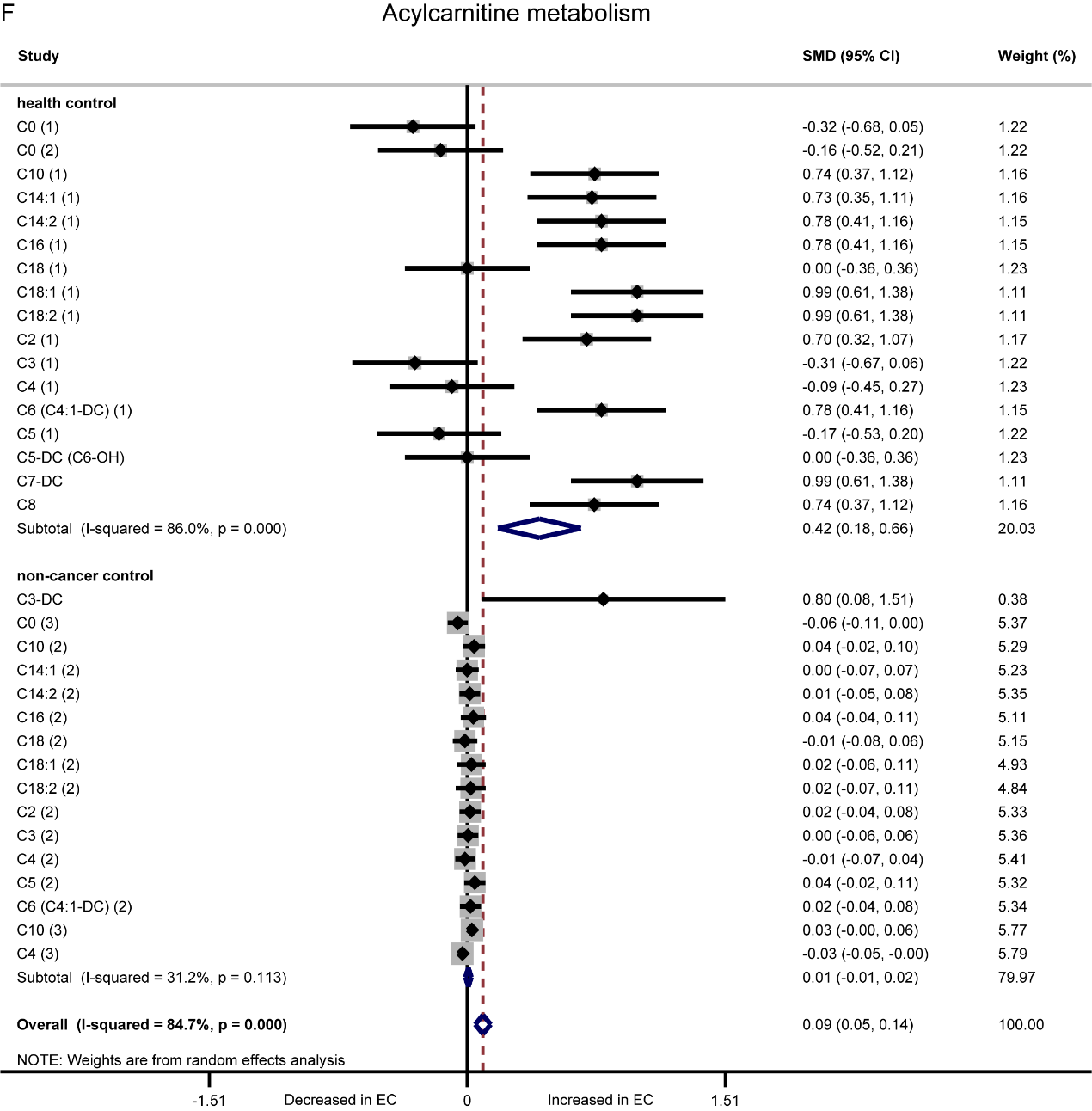


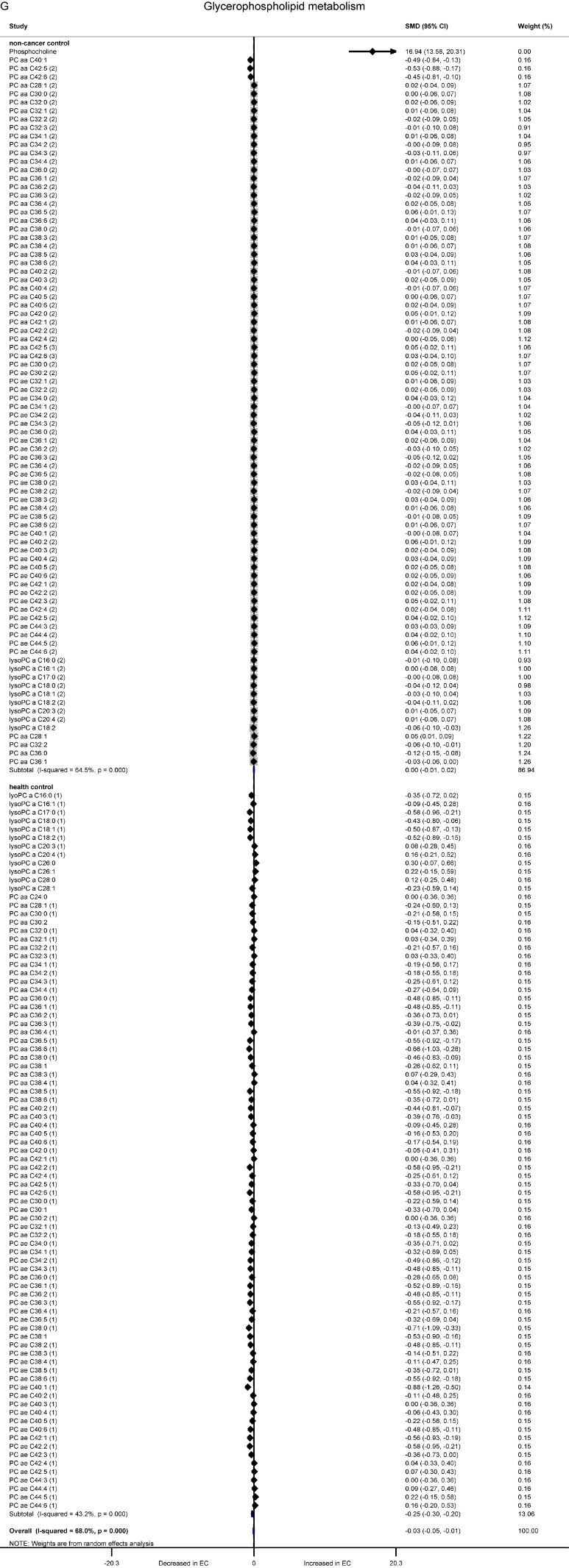


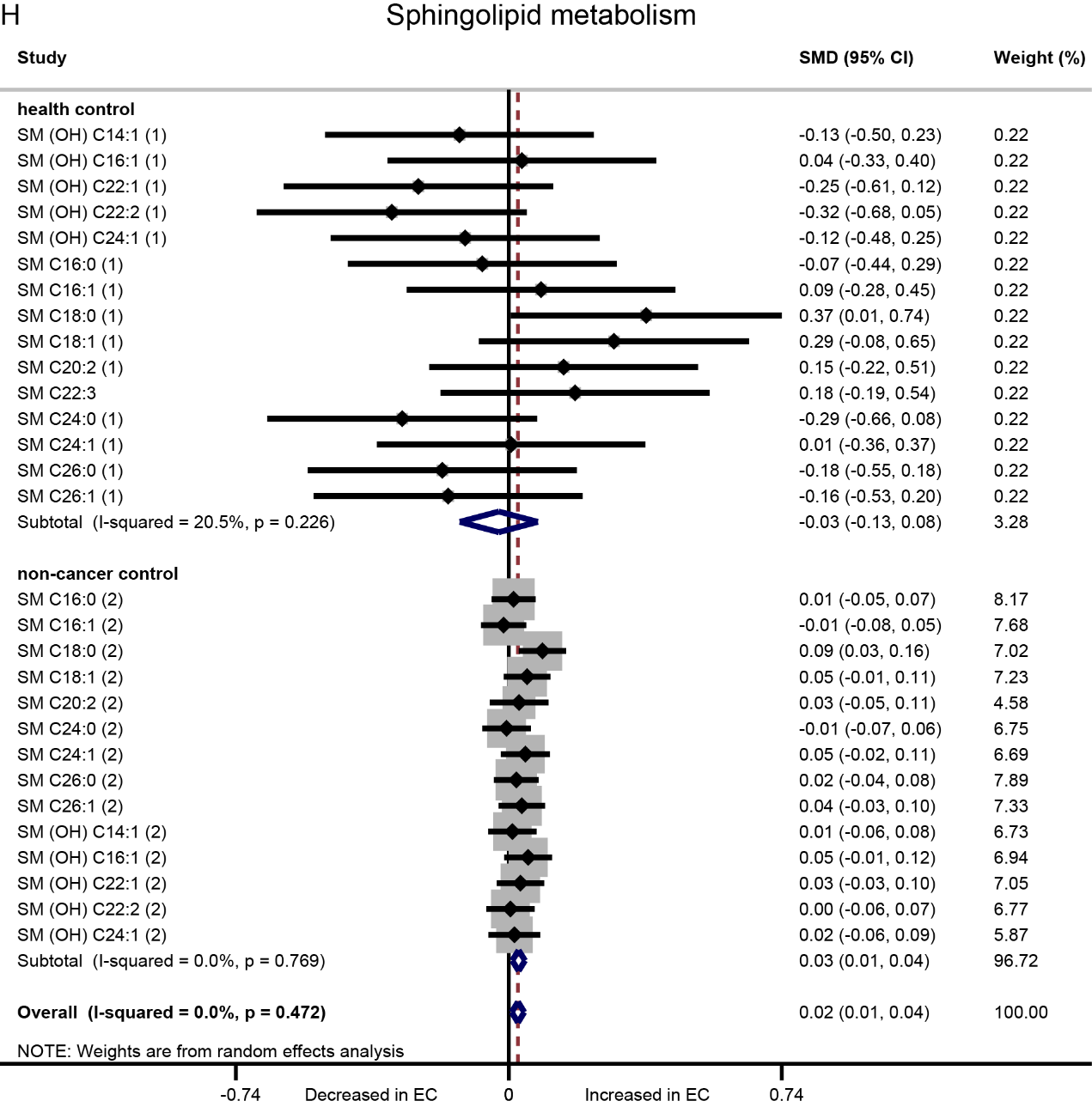


**Figure S5: Forest plots of control group type subgroup analysis of endometrial cancer-associated metabolic pathways. (A) Carbohydrate metabolism; (B) Alanine, aspartate, and glutamate metabolism; (C) BCAA metabolism; (D) Arginine and proline metabolism, Urea cycle; (E) Glycine, serine, and threonine metabolism; (F) Acylcarnitine metabolism; (G) Glycerophospholipid metabolism; (H) Sphingolipid metabolism.** BCAA, branched-chain amino acid; C0, carnitine; C10, decanoylcarnitine; C14:1, tetradecenoylcarnitine; C14:2, tetradecadienoylcarnitine; C16, hexadecenoylcarnitine; C18, 3-hydroxylhexadecanoylcarnitine; C18:1, octadecenoylcarnitine; C18:2, octadecadienoylcarnitine; C2, acetylcarnitine; C3, propionylcarnitine; C3-DC, malonylcarnitine; C4, malonylcarnitine; C5, methylmalonylcarnitine; C5-DC, 3-hydroxyl-isovalerylcarnitine; C6, glutarylcarnitine; C7-DC, pimelylcarnitine; C8, 3-methylglutarylcarnitine; LysoPC, lysophosphatidylcholine; PC, Phosphatidylcholine; SM, sphingomyelin; SMD, standardized mean difference.

**Supplementary References: supplementary references for included articles.**

[1] Bufa A, Bíró I, Poór V, et al. Altered urinary profiles of endogenous steroids in postmenopausal women with adenocarcinoma endometrii. *Gynecol Endocrinol.* 2010;26(1):10-15. doi:10.3109/09513590903159581

[2] Audet-Delage Y, Grégoire J, Caron P, et al. Estradiol metabolites as biomarkers of endometrial cancer prognosis after surgery. *J Steroid Biochem Mol Biol.* 2018;178:45-54. doi:10.1016/j.jsbmb.2017.10.021

[3] Skorupa A, Poński M, Ciszek M, et al. Grading of endometrial cancer using 1H HR-MAS NMR-based metabolomics. *Sci Rep.* 2021;11(1):18160. doi:10.1038/s41598-021-97505-y

[4] Shi K, Wang Q, Su Y, et al. Identification and functional analyses of differentially expressed metabolites in early stage endometrial carcinoma. *Cancer Sci.* 2018;109(4):1032-1043. doi:10.1111/cas.13532

[5] Audet-Delage Y, Villeneuve L, Grégoire J, Plante M, Guillemette C. Identification of Metabolomic Biomarkers for Endometrial Cancer and Its Recurrence after Surgery in Postmenopausal Women. *Front Endocrinol (Lausanne).* 2018;9:87. doi:10.3389/fendo.2018.00087

[6] Kozar N, Kruusmaa K, Dovnik A, et al. Identification of novel diagnostic biomarkers in endometrial cancer using targeted metabolomic profiling. *Adv Med Sci.* 2021;66(1):46-51. doi:10.1016/j.advms.2020.12.001

[7] Cummings M, Massey KA, Mappa G, et al. Integrated eicosanoid lipidomics and gene expression reveal decreased prostaglandin catabolism and increased 5-lipoxygenase expression in aggressive subtypes of endometrial cancer. *J Pathol.* 2019;247(1):21-34. doi:10.1002/path.5160

[8] Shafiee MN, Ortori CA, Barrett DA, Mongan NP, Abu J, Atiomo W. Lipidomic Biomarkers in Polycystic Ovary Syndrome and Endometrial Cancer. *Int J Mol Sci.* 2020;21(13):4753. doi:10.3390/ijms21134753

[9] Altadill T, Dowdy TM, Gill K, et al. Metabolomic and Lipidomic Profiling Identifies The Role of the RNA Editing Pathway in Endometrial Carcinogenesis. *Sci Rep.* 2017;7(1):8803. doi:10.1038/s41598-017-09169-2

[10] Njoku K, Campbell AE, Geary B, et al. Metabolomic Biomarkers for the Detection of Obesity-Driven Endometrial Cancer. *Cancers (Basel).* 2021;13(4):718. doi:10.3390/cancers13040718

[11] Cheng SC, Chen K, Chiu CY, et al. Metabolomic biomarkers in cervicovaginal fluid for detecting endometrial cancer through nuclear magnetic resonance spectroscopy. *Metabolomics.* 2019;15(11):146. doi:10.1007/s11306-019-1609-z

[12] Bahado-Singh RO, Lugade A, Field J, et al. Metabolomic prediction of endometrial cancer. *Metabolomics.* 2017;14(1):6. doi:10.1007/s11306-017-1290-z

[13] Troisi J, Sarno L, Landolfi A, et al. Metabolomic Signature of Endometrial Cancer. *J Proteome Res.* 2018;17(2):804-812. doi:10.1021/acs.jproteome.7b00503

[14] Jové M, Gatius S, Yeramian A, et al. Metabotyping human endometrioid endometrial adenocarcinoma reveals an implication of endocannabinoid metabolism. *Oncotarget.* 2016;7(32):52364-52374. doi:10.18632/oncotarget.10564

[15] Knific T, Vouk K, Smrkolj Š, Prehn C, Adamski J, Rižner TL. Models including plasma levels of sphingomyelins and phosphatidylcholines as diagnostic and prognostic biomarkers of endometrial cancer. *J Steroid Biochem Mol Biol.* 2018;178:312-321. doi:10.1016/j.jsbmb.2018.01.012

[16] Schuhn A, Tobar TW, Gahlawat AW, et al. Potential of blood-based biomarker approaches in endometrium and breast cancer: a case-control comparison study. *Arch Gynecol Obstet.* 2022;306(5):1623-1632. doi:10.1007/s00404-022-06482-8

[17] Dossus L, Kouloura E, Biessy C, et al. Prospective analysis of circulating metabolites and endometrial cancer risk. *Gynecol Oncol.* 2021;162(2):475-481. doi:10.1016/j.ygyno.2021.06.001

[18] Shao X, Wang K, Liu X, et al. Screening and verifying endometrial carcinoma diagnostic biomarkers based on a urine metabolomic profiling study using UPLC-Q-TOF/MS. *Clin Chim Acta.* 2016;463:200-206. doi:10.1016/j.cca.2016.10.027

[19] Yan X, Zhao W, Wei J, et al. A serum lipidomics study for the identification of specific biomarkers for endometrial polyps to distinguish them from endometrial cancer or hyperplasia. *Int J Cancer.* 2022;150(9):1549-1559. doi:10.1002/ijc.33943

[20] Yi R, Xie L, Wang X, Shen C, Chen X, Qiao L. Multi-Omic Profiling of Multi-Biosamples Reveals the Role of Amino Acid and Nucleotide Metabolism in Endometrial Cancer. *Front Oncol.* 2022;12:861142. doi:10.3389/fonc.2022.861142

[21] Lépine J, Audet-Walsh E, Grégoire J, et al. Circulating estrogens in endometrial cancer cases and their relationship with tissular expression of key estrogen biosynthesis and metabolic pathways. *J Clin Endocrinol Metab.* 2010;95(6):2689-2698. doi:10.1210/jc.2010-2648

[22] Zhao SS, Chen L, Yang J, et al. Altered Gut Microbial Profile Accompanied by Abnormal Fatty Acid Metabolism Activity Exacerbates Endometrial Cancer Progression. *Microbiol Spectr.* 2022;10(6):e0261222. doi:10.1128/spectrum.02612-22

[23] Hishinuma E, Shimada M, Matsukawa N, et al. Identification of predictive biomarkers for endometrial cancer diagnosis and treatment response monitoring using plasma metabolome profiling. *Cancer Metab.* 2023;11(1):16. doi:10.1186/s40170-023-00317-z

[24] Boyd AE, Grizzard PJ, Hylton Rorie K, Lima S. Lipidomic Profiling Reveals Biological Differences between Tumors of Self-Identified African Americans and Non-Hispanic Whites with Cancer. *Cancers (Basel).* 2023;15(8):2238. doi:10.3390/cancers15082238

[25] Arda Düz S, Mumcu A, Doğan B, et al. Metabolomic analysis of endometrial cancer by high-resolution magic angle spinning NMR spectroscopy. *Arch Gynecol Obstet.* 2022;306(6):2155-2166. doi:10.1007/s00404-022-06587-0

[26] Troisi J, Mollo A, Lombardi M, et al. The Metabolomic Approach for the Screening of Endometrial Cancer: Validation from a Large Cohort of Women Scheduled for Gynecological Surgery. *Biomolecules.* 2022;12(9):1229. doi:10.3390/biom12091229

[27] Gu M, Chen X, Sun Y, Wang L, Shu H, Qian C. A metabolomic signature of FIGO stage I and II endometrial cancer. *Neoplasma.* 2021;68(6):1283-1291. doi:10.4149/neo_2021_210306N288

[28] Breeur M, Ferrari P, Dossus L, et al. Pan-cancer analysis of pre-diagnostic blood metabolite concentrations in the European Prospective Investigation into Cancer and Nutrition. *BMC Med.* 2022;20(1):351. doi:10.1186/s12916-022-02553-4

[29] Hao C, Lin S, Liu P, Liang W, Li Z, Li Y. Potential serum metabolites and long-chain noncoding RNA biomarkers for endometrial cancer tissue. *J Obstet Gynaecol Res.* 2023;49(2):725-743. doi:10.1111/jog.15494

[30] Cheng F, Fan W, Gui L, et al. Serum lipidomic profiling by UHPLC-MS/MS may be able to detect early-stage endometrial cancer. *Anal Bioanal Chem.* 2023;415(10):1841-1854. doi:10.1007/s00216-023-04586-x
